# Gold-catalyzed regioselective oxidation of propargylic carboxylates: a reliable access to α-carboxy-α,β-unsaturated ketones/aldehydes

**DOI:** 10.3762/bjoc.9.227

**Published:** 2013-09-24

**Authors:** Kegong Ji, Jonathan Nelson, Liming Zhang

**Affiliations:** 1 Department of Chemistry and Biochemistry, University of California, Santa Barbara, California, 93106, USA

**Keywords:** enone, gold catalysis, oxidation, propargyl carboxylate

## Abstract

Gold-catalyzed intermolecular oxidation of carboxylates of primary or secondary propargylic alcohols are realized with excellent regioselectivity, which is ascribed to inductive polarization of the C–C triple bond by the electron-withdrawing carboxy group. The gold carbene intermediates thus generated undergo selective 1,2-acyloxy migration over a 1,2-C–H insertion, and the selectivities could be dramatically improved by the use of a *P,S*-bidentate ligand, which is proposed to enable the formation of tris-coordinated and hence less electrophilic gold carbene species. α-Carboxy α,β-unsaturated ketones/aldehydes can be obtained with fair to excellent yields.

## Introduction

**Scheme 1:**
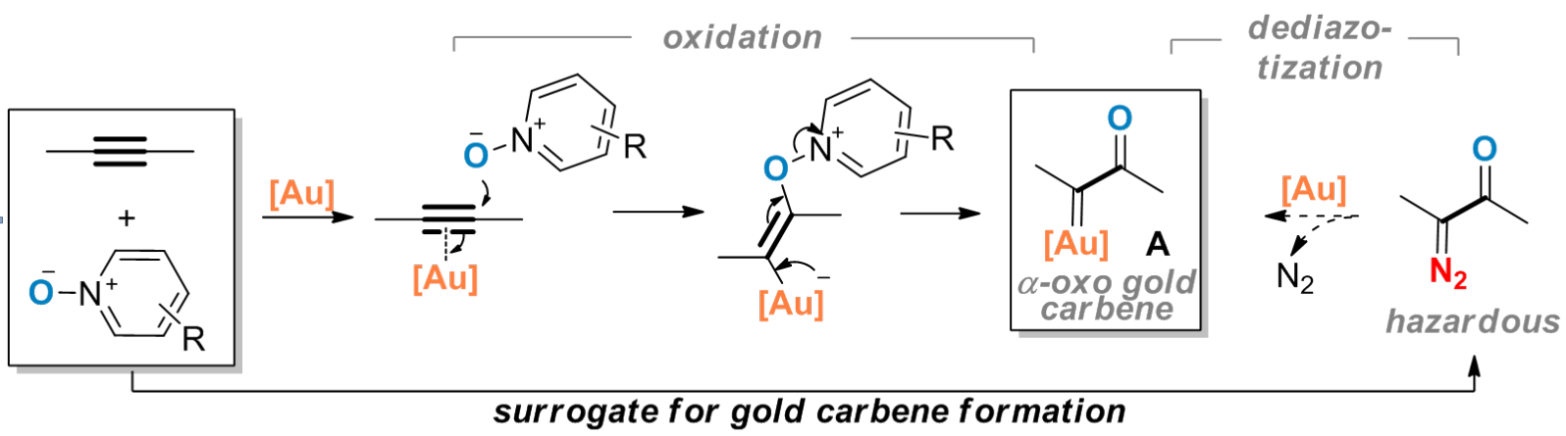
Generation of α-oxo gold carbenes via intermolecular oxidation of alkynes: a non-diazo approach.

We reported in 2010 [1] that α-oxo gold carbenes could be conveniently generated as reactive intermediates in gold-catalyzed intermolecular oxidation of alkynes. By using pyridine *N-*oxides [1] and later 8-substituted quinoline *N*-oxides [2] as the external oxidants, this approach permits a safe and efficient access to α-oxo gold carbenes without resorting to the dediazotization strategy [3–5] using hazardous and potentially explosive diazo substrates (Scheme 1). Since then an array of versatile synthetic methods has been developed based on the general approach by us [2,6–12] and other researchers [13–20], thus making it an exciting area for further advancing gold chemistry.

**Scheme 2:**
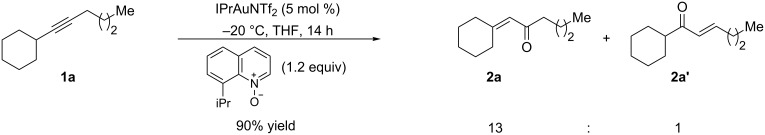
Gold-catalyzed regioselective oxidation of a sterically biased internal alkyne.

Among various types of alkynes examined, internal alkynes, while without incident in the generation of the gold carbene intermediates, present an additional challenge, namely how to control the regioselectivity of the oxidation. We reported previously that synthetically useful regioselectivity could be achieved if the two ends of the C–C triple bond are biased by a steric bulk and/or via conjugation (Scheme 2). In our continued effort to reveal regioselectivities of this oxidation with different types of internal alkynes, we examined propargylic carboxylates, which have served as a versatile platform for the development of a diverse range of gold catalysis [21]. Herein we report our findings and the development of a reliable synthesis of α-carboxy α,β-unsaturated ketones/aldehydes.

## Results and Discussion

**Scheme 3:**
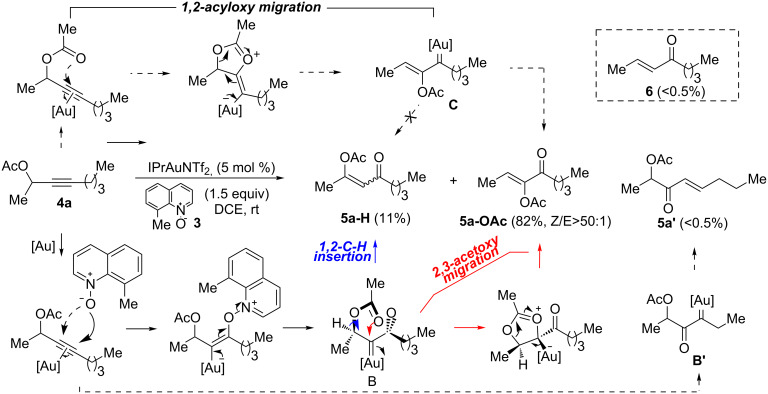
Gold-catalyzed oxidation of the propargylic acetate **4a** and the mechanistic rationale.

We began by subjecting the propargylic acetate **4a** to the suitable conditions developed in our previous study, namely IPrAuNTf_2_ (5 mol %) and 8-methylquinoline *N*-oxide (**3**, 1.5 equiv) in 1,2-dichloroethane at ambient temperature. To our delight, the reaction proceeded efficiently, yielding the α-acetoxyenone **5a-OAc** (*Z*/*E* >50:1) and the isomeric β-acetoxyenone **5a-H** in an excellent combined 92% yield along with a minute amount of the enone **6** (<0.5%, Scheme 3); moreover, **5a-OAc** is favored over **5a-H** by a ratio of ~7:1. Of particular importance is that the anticipated isomer **5a’**, accessible via the gold carbene **B’** from a regioisomeric alkyne oxidation, was not positively detected due to the trace amount (<0.5%), thereby revealing an exceptional level of regioselectivity in the oxidation of this type of internal alkynes. The formations of **5a-OAc** and **5a-H** are rationalized as the results of divergent transformations of the α-oxo gold carbene **B**: the former is formed via a two-step 2,3-acetoxy migration [22–23], and the latter most likely via a concerted carbene 1,2-C–H insertion[2]. The selective formation of the *Z* isomer of **5a**-**OAc** can be attributed to that **B** adopts a preferred conformation, as detailed in Scheme 3, in order to avoid steric interaction between Me and the acyl moiety. It needs to be noted that a related intramolecular version of this reaction has been previously reported [24].

**Scheme 4:**
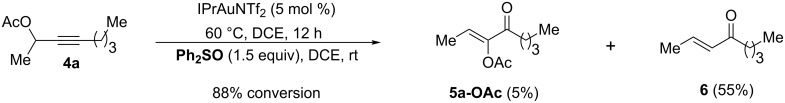
A drastically different outcome by using diphenyl sulfoxide as the oxidant.

An alternative mechanism for the formation of **5a-OAc** is also shown in Scheme 3 (the top half). Instead of initially undergoing oxidative gold carbene formation, a gold-promoted 1,2-acetoxy migration [25] would generate a vinyl gold carbene intermediate (i.e., **C**), which can then be oxidized by **3** to yield the product. However, this scenario is deemed unlikely by the following observations and considerations: a) propargylic carboxylates of type **4a** with an internal C–C triple bond typically undergo facile 3,3-rearrangements [26–30] instead of 1,2-acyloxy migrations. The former process would eventually lead to the formation of the enones **6** [31]. Due to hydrolysis, only under thermal and anhydrous conditions products derived from the latter processes can be predominantly formed [32]; under our conditions (at ambient temperature and without exclusion of moisture), the enone **6** was indeed detected but in a minute amount, suggesting that the 1,2-acyloxy migration might be an even less meaningful event in the reaction; b) it is known that the gold carbenes of type **B** can be readily oxidized by Ph_2_S=O [33], which, however, is an inefficient oxidant for generating α-oxo gold carbenes of type **A** via alkyne oxidation [34–35]; when the *N*-oxide **3** is replaced by the sulfoxide, **5a-OAc** was formed in only 5% yield even at 60 °C after 12 h (Scheme 4); moreover, the major product in the reaction was the expected enone **6** (56% yield, 88% conversion) due to a dominant gold-catalyzed 3,3-rearrangement; c) this alternative could not rationalize the formation of **5a-H**.

The fact that in the presence of the oxidant **3** the previously observed facile transformations of propargylic carboxylates (i.e., 3,3-rearrangement and 1,2-acyloxy migration) are no longer competitive with the oxidative catalysis is surprising and suggests that this oxidation process could divert substrates from other well established, facile gold catalysis to the formation of distinctively different functional products in the presence of oxidants.

**Figure 1:**
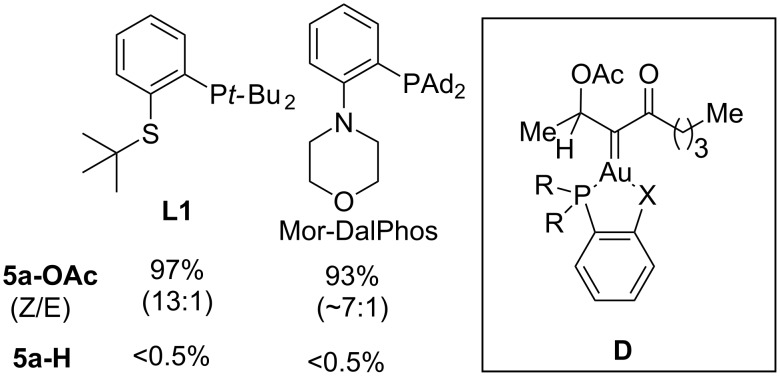
The impact of ligands on the ratio of **5a-OAc** and **5a-H** in the gold-catalyzed oxidation of **4a** (reaction conditions: 5 mol % gold catalyst, 1.5 equiv of **3**, DCE, rt, 3 h).

The relatively low selectivity (i.e., ~7:1) of **5a-OAc** over **5a-H** was drastically improved upon catalyst screening. It was eventually found that the ratio could reach >200:1 by using the gold(I) catalyst derived from our previously developed bulky *P,S*-bidentate ligand **L1** (Figure 1) [11]. A similarly high selectivity was also achieved by using the *P,N*-bidentate ligand Mor-DalPhos [36–37]. However, the *Z*/*E* ratios of **5a-OAc** in the former case is ~13:1, better than ~7:1 in the latter case, albeit both lower than that by IPrAuNTf_2_ (>50:1, see Scheme 3). The enhanced preference of AcO migration en route to the formation of **5a-OAc** over the 1,2-C-H insertion is attributed to attenuation of the electrophilicity of the gold carbene moiety via the formation of a tris-coordinated gold complex (i.e., **D**) [11].

The scope of this alkyne oxidation/acetoxy migration reaction is outlined in Table 1. Acetates derived from primary/secondary propargylic alcohols with various substitution patterns and containing different functional groups were all allowed, although the tertiary counterpart underwent gold-catalyzed 3,3-rearrangement preferentially [21] and hence was not a viable substrate. Except entry 7, the gold-catalyzed oxidations proceeded with excellent regioselectivities (>25:1), and the desired α-acyloxy α,β-unsaturated ketones/aldehyde were isolated with fair to excellent yields. While the bulky catalyst Me_4_*t*-BuXPhosAuNTf_2_ [38] was used in entry 1 to obtain a better oxidation regioselectivity (28/1), it did not lead to a good ratio in the case of **4h** (entry 7), where the oxidation regioisomer of type **5a’** was formed in 23% yield. This outcome is rationalized in the next paragraph. In the case of pivalate **3c** with a terminal alkyne (entry 2), the use of this bulky acyl group instead of acetyl is to curtail the hydrolytic formation of the corresponding α-ketoaldehyde. In many cases the ratios of **5-OAc** and **5-H** were high with IPrAuNTf_2_ as the catalyst; for the ones with low selectivities, **L1**AuNTf_2_ offered again dramatic improvements (entries 5, 8 and 9) although at the expense of the geometric selectivities of the major product.

**Table 1:** Reaction scope studies for the formation of α-acetoxyenones from propargyl acetates.^a^

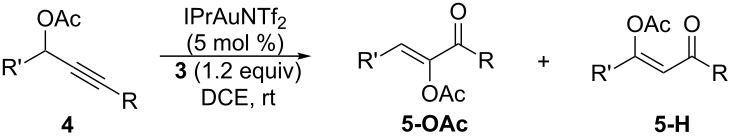							

entry	**4**	**5**	yield^b^ ratio*^c^* time	entry	**4**	**5**	yield^b^ ratio^c^

1^d^	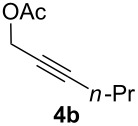	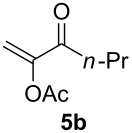	80% 14/1 3 h	7^d^	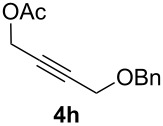	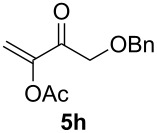	62% 21/1^h^ 5 h
2	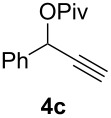	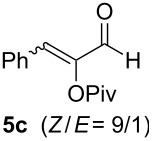	74%^e^ >100:1 2.5 h	8^f^	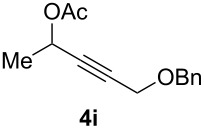	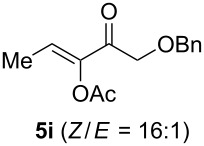	60% >200/1 6.5 h
3	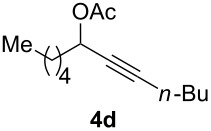	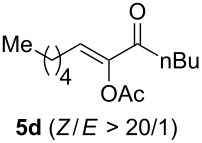	86% >50/1 12 h	9^f^	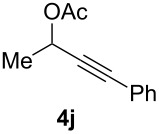	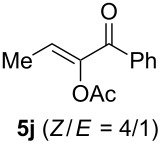	75% >100/1 2.5 h
4	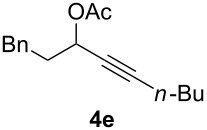	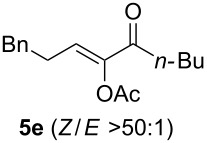	85% >50/1 9 h	10	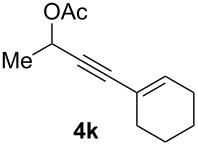	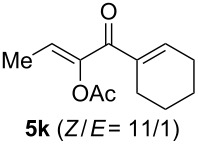	90% >20/1 10 h
5^f,g^	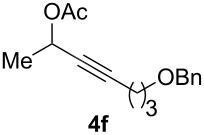	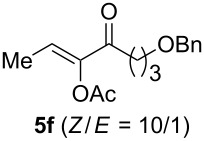	75% >200/1 2.5 h	11	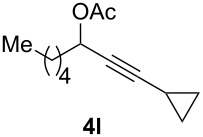	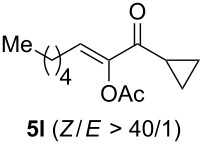	84% >35/1 5 h
6	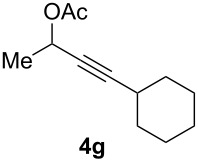	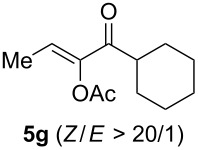	76% 33/1 7 h	12	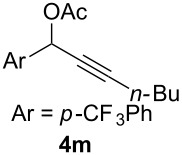	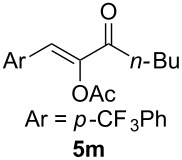	85% >20/1 10 h

^a^[**4**] = 0.05 M. ^b^Isolated yield of **5-OAc**. ^c^**5-OAc**/**5-H**. ^d^Me_4_*t-*BuXPhosAuNTf_2_ was used as the catalyst. ^e^IPrAuCl/AgSbF_6_ as the catalyst, 3,5-dicholoropyridine *N*-oxide (2 equiv) as the oxidant, and DCM as the solvent. ^f^**L1**AuCl/AgNTf_2_ used as catalyst. ^g^10 mol % catalyst. ^h^The oxidation regioisomer of type **5a’** was formed in a 23% yield.

**Figure 2:**
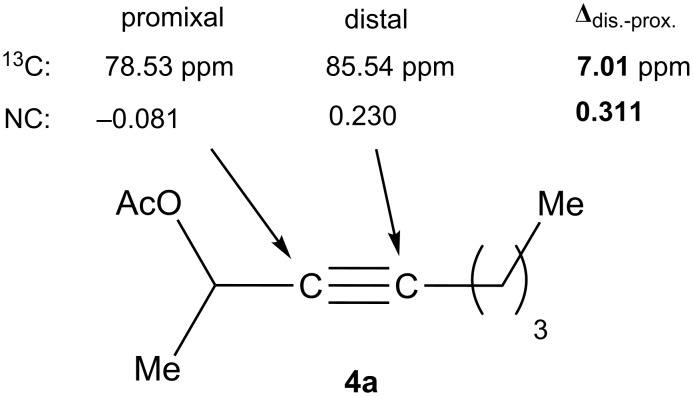
Natural charges at and the ^13^C chemical shifts of the alkynyl carbons in **4a**.

The excellent regioselectivities of gold-catalyzed oxidations of propargylic carboxylates, albeit unexpected, could be readily rationalized by invoking inductive polarization of the C–C triple bond by the electronegative carboxy group. Such polarization could be revealed by calculated natural charges via natural population analysis [39] and corroborated by experimentally detectable properties such as p*K*_a_ [40] and ^13^C NMR [41]. We calculated the natural charges at the sp-hybridized carbons in **4a** using the Density Functional Theory (B3LYP/6-31G*, Spartan06). The NC is 0.230 for the C(sp) distal to the carboxy group and −0.081 for the proximal C(sp), revealing a strong inductive effect that leads to a more electron-defficient distal alkyne end (Figure 2). This revelation is consistent with the ^13^C NMR chemical shifts of the alkynyl carbons. The observed regioselectivity can be ascribed to a selective attack of the nucleophilic oxidant to the more electrophilic distal C(sp). Notably, a recently published Pt-catalyzed hydrosilylation on a similar substrate showed a 3.7:1 regioselectivity [42]. This unexpectedly high selectivity with gold catalysis is attributed to the augmentation of the electronic bias of the C–C triple bond via the gold activation. The decreased regioselectivity with **4h** (entry 7) is due to the counter polarization of the C–C triple bond by the propargylic BnO group. The better result with **4i** also containing a similarly positioned BnO group (entry 8) is attributed to the synergistic effect of the steric bias [2].

While a previous Pd catalysis [43] could also accomplish this transformation, the demonstrated scope is much limited, and the catalyst loading is 20%. With this oxidative gold catalysis, the propargyl esters, except those derived from tertiary alcohols, can be reliably converted into α-acyloxy α,β-unsaturated ketones/aldehydes.

## Conclusion

We have realized a gold-catalyzed, highly regioselective oxidation of carboxylates of primary and secondary propargylic alcohols by utilizing inductive polarization of the C-C triple bond by the electron-withdrawing carboxy moiety. The α-oxo gold carbene intermediates generated can selectively undergo 1,2-acyloxy migrations over 1,2-C–H insertion. This inherent selectivity can be much enhanced by the use of our previously developed *P,S*-bidentate ligand, which enables the generation of tri-coordinated and less electrophilic gold carbene species. α-Acyloxy α,β-unsaturated ketones/aldehydes can be obtained with fair to excellent yields.

## Supporting Information

File 1 Experimental procedure, compound characterization, and NMR spectra.

## References

[1] Ye L, Cui L, Zhang G, Zhang L (2010). J Am Chem Soc.

[2] Lu B, Li C, Zhang L (2010). J Am Chem Soc.

[3] Doyle M P, McKevey M A, Ye T (1998). Modern Catalytic Methods for Organic Synthesis with Diazo Compounds: From Cyclopropanes to Ylides.

[4] Davies H M L, Beckwith R E J (2003). Chem Rev.

[5] Taber D F, Pattenden G (1991). Carbon-Carbon Σ-Bond Formation.

[6] Ye L, He W, Zhang L (2010). J Am Chem Soc.

[7] He W, Li C, Zhang L (2011). J Am Chem Soc.

[8] Ye L, He W, Zhang L (2011). Angew Chem, Int Ed.

[9] Wang Y, Ji K, Lan S, Zhang L (2012). Angew Chem, Int Ed.

[10] Ji K, Zhao Y, Zhang L (2013). Angew Chem, Int Ed.

[11] Luo Y, Ji K, Li Y, Zhang L (2012). J Am Chem Soc.

[12] He W, Xie L, Xu Y, Xiang J, Zhang L (2012). Org Biomol Chem.

[13] Bhunia S, Ghorpade S, Huple D B, Liu R-S (2012). Angew Chem, Int Ed.

[14] Vasu D, Hung H-H, Bhunia S, Gawade S A, Das A, Liu R-S (2011). Angew Chem, Int Ed.

[15] Henrion G, Chava T E J, Le Goff X, Gagosz F (2013). Angew Chem, Int Ed.

[16] Hashmi A S K, Wang T, Shi S, Rudolph M (2012). J Org Chem.

[17] Xu M, Ren T-T, Li C-Y (2012). Org Lett.

[18] Dateer R B, Pati K, Liu R-S (2012). Chem Commun.

[19] Qian D, Zhang J (2011). Chem Commun.

[20] Davies P W, Cremonesi A, Martin N (2011). Chem Commun.

[21] Wang S, Zhang G, Zhang L (2010). Synlett.

[22] Mamane V, Gress T, Krause H, Fuerstner A (2004). J Am Chem Soc.

[23] Harrak Y, Blaszykowski C, Bernard M, Cariou K, Mainetti E, Mouriès V, Dhimane A-L, Fensterbank L, Malacria M (2004). J Am Chem Soc.

[24] Murai M, Kitabata S, Okamoto K, Ohe K (2012). Chem Commun.

[25] Johansson M J, Gorin D J, Staben S T, Toste F D (2005). J Am Chem Soc.

[26] Zhang L (2005). J Am Chem Soc.

[27] Wang S, Zhang L (2006). J Am Chem Soc.

[28] Wang S, Zhang L M (2006). Org Lett.

[29] Zhang L, Wang S (2006). J Am Chem Soc.

[30] Marion N, Díez-González S, de Fremont P, Noble A R, Nolan S P (2006). Angew Chem, Int Ed.

[31] Yu M, Li G, Wang S, Zhang L (2007). Adv Synth Catal.

[32] Li G, Zhang G, Zhang L (2008). J Am Chem Soc.

[33] Witham C A, Mauleon P, Shapiro N D, Sherry B D, Toste F D (2007). J Am Chem Soc.

[34] Cuenca A B, Montserrai S, Hossain K M, Mancha G, Lledós A, Medio-Simón M, Ujaque G, Asensio G (2009). Org Lett.

[35] Li C-W, Pati K, Lin G-Y, Sohel S M A, Hung H-H, Liu R-S (2010). Angew Chem, Int Ed.

[36] Lundgren R J, Peters B D, Alsabeh P G, Stradiotto M (2010). Angew Chem, Int Ed.

[37] Hesp K D, Stradiotto M (2010). J Am Chem Soc.

[38] Wang Y, Ji K, Lan S, Zhang L (2012). Angew Chem, Int Ed.

[39] Reed A E, Weinstock R B, Weinhold F (1985). J Chem Phys.

[40] Gross K C, Seybold P G, Hadad C M (2002). Int J Quant Chem.

[41] Levy J B (1999). Struct Chem.

[42] Rooke D A, Ferreira E M (2012). Angew Chem, Int Ed.

[43] Bartels A, Mahrwald R, Müller K (2004). Adv Synth Catal.

