# Supplementary material for: Gold-catalyzed regioselective oxidation of propargylic carboxylates: a reliable access to α-carboxy-α,β-unsaturated ketones/aldehydes
Source: Beilstein J Org Chem. 2013 Sep 24;9:1925–30. doi: 10.3762/bjoc.9.227 (PMC3817591; doi:10.3762/bjoc.9.227)

Supporting Information

for

Gold-catalyzed regioselective oxidation of propargylic carboxylates: a reliable access to α-carboxy-α,β-unsaturated ketones/aldehydes

Kegong Ji, Jonathan Nelson and Liming Zhang*

Address: Department of Chemistry and Biochemistry, University of California, Santa Barbara, California, 93106, USA

Email: Liming Zhang - zhang@chem.ucsb.edu

* Corresponding author

Experimental procedure, compound characterization and NMR spectra

Content	Page number
**General**	s2
**General procedure A: Preparation of propargylic acetate.**	s2
**General procedure B: Gold-catalyzed oxidation/acetoxy migration reaction of propargyl acetates to α-acetoxyenones**	s7
**^{1}H and ^{13}C NMR spectra**	s13

General. 1,2-Dichloroethane (HPLC grade), ethyl acetate (ACS grade), hexanes (ACS grade) and diethyl ether (ACS grade) were purchased from Fisher Scientific and used without further purification. Anhydrous tetrahydrofuran in Pure-Pac™ from Aldrich was used directly without further purification. Commercially available reagents were used without further purification. Reactions were monitored by thin layer chromatography (TLC) using silicycle pre-coated silica gel plates. Flash column chromatography was performed over silicycle silica gel (230–400 mesh). ^{1}H NMR and ^{13}C NMR spectra were recorded on a Varian 500 MHz Unity plus spectrometer and a Varian 600 MHz spectrometer using residue solvent peaks as internal standards. Infrared spectra were recorded with a Perkin Elmer FT-IR spectrum 2000 spectrometer and are reported in reciprocal centimeter (cm^{-1}). Mass spectra were recorded with Micromass QTOF2 Quadrupole/Time-of-Flight Tandem mass spectrometer using electron spray ionization.

General Procedure A: Preparation of propargylic acetate

To a solution of the propargylic alcohol (2.0 mmol), pyridine (1.65 mL, 20.0 mmol) and catalytic amount of DMAP in anhydrous CH_2Cl_2 (6.0 mL) at 0 °C, was slowly added acetyl chloride (0.29 mL, 4.0 mmol). The reaction was stirred at the same temperature for 30 min before being diluted with hexanes (30 mL). The solid precipitates were filtered off and the filtrate obtained was concentrated. The residue was purified through silica gel flash column chromatography (hexanes/ethyl acetate = 20/1) to yield the desired acetate **4**.

Oct-3-yn-2-yl acetate (4a)

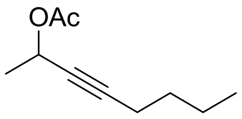

4a

This known compound **4a** was prepared in 90% yield through the General Procedure A and its spectroscopic data were in accordance with the literature data. [1] ^{1}H NMR (500 MHz, $CDCl_3$) δ 5.43 (qt, *J* = 6.6, 2.0 Hz, 1H), 2.19 (td, *J* = 7.1, 2.0 Hz, 2H), 2.06 (s, 3H), 1.51 – 1.43 (m, 5H), 1.40-1.36 (m, 2H), 0.90 (t, *J* = 7.3 Hz, 3H). ^{13}C NMR (126 MHz, $CDCl_3$) δ 169.96, 85.54, 78.53, 60.84, 30.53, 21.88, 21.81, 21.15, 18.33, 13.55.

Hex-2-ynyl acetate (4b)

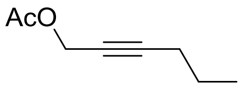

4b

This compound **4b** was prepared in 88% yield through the General Procedure A. ^{1}H NMR (500 MHz, $CDCl_3$) δ 4.66 (dt, *J* = 2.2, 1.1 Hz, 2H), 2.21-2.09 (m, 2H), 2.09 (s, 3H), 1.53 (h, *J* = 7.3 Hz, 2H), 0.97 (t, *J* = 7.3Hz, 3H). ^{13}C NMR (126 MHz, $CDCl_3$) δ 170.37, 87.54, 74.00, 52.86, 21.84, 20.82, 20.71, 13.43. IR(neat): 3392, 2969, 1749, 1380, 1226, 1033. ESI ($M+Na^+$): 163.01.

1-Phenylprop-2-ynyl pivalate (4c)

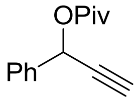

4c

This known compound **4c** was prepared in 90% yield through the General Procedure A and its spectroscopic data were in accordance with the literature data. [2] ^{1}H NMR (600 MHz, $CDCl_3$) δ 7.56 – 7.47 (m, 2H), 7.41 – 7.33 (m, 3H), 6.43 (d, *J* = 2.2 Hz, 1H), 2.62 (d, *J* = 2.3 Hz, 1H), 1.23 (s, 9H). ^{13}C NMR (151 MHz, $cdcl_3$) δ 177.10, 136.77, 128.78, 128.61, 127.24, 80.43, 75.05, 65.03, 38.72, 26.94.

Dodec-7-yn-6-yl acetate (4d)

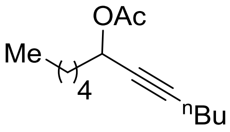

4d

This known compound **4d** was prepared in 92% yield through the General Procedure A and its spectroscopic data were in accordance with the literature data. [3] ^{1}H NMR (600 MHz, $CDCl_3$) δ 5.34 (tt, *J* = 6.7, 1.9 Hz, 1H), 2.20 (td, *J* = 7.1, 2.0 Hz, 2H), 2.06 (s, 3H), 1.73-1.67 (m, 2H), 1.51 – 1.44 (m, 2H), 1.44 – 1.37 (m, 4H), 1.31-1.29 (m, 4H), 0.90 (t, *J* = 6.9 Hz, 6H). ^{13}C NMR (151 MHz, $CDCl_3$) δ 170.12, 86.15, 77.64, 64.65, 35.07, 31.28, 30.57, 24.69, 22.48, 21.88, 21.15, 18.37, 13.94, 13.55.

1-Phenylnon-4-yn-3-yl acetate (4e)

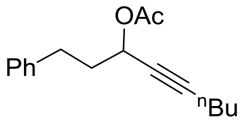

4e

This compound **4e** was prepared in 90% yield through the General Procedure A. ^{1}H NMR (500 MHz, $CDCl_3$) δ 7.31 – 7.26 (m, 2H), 7.20 (dd, *J* = 7.3, 5.0 Hz, 3H), 5.37 (tt, *J* = 6.5, 2.0 Hz, 1H), 2.77 (t, *J* = 8.0 Hz, 2H), 2.24 (td, *J* = 7.1, 2.0 Hz, 2H), 2.12 – 1.96 (m, 2H), 2.06(s, 3H), 1.53 – 1.47 (m, 2H), 1.46 – 1.38 (m, 2H), 0.92 (t, *J* = 7.3 Hz, 3H). ^{13}C NMR (126 MHz, $CDCl_3$) δ 170.00, 140.98, 128.42, 128.36, 126.02, 86.71, 77.29, 64.11, 36.66, 31.36, 30.56, 21.91, 21.07, 18.39, 13.56. IR(neat):2934, 2864, 1742, 1371, 1232, 1021, 748. ESI ($M+Na^+$):281.16.

7-(Benzyloxy)hept-3-yn-2-yl acetate (4f)

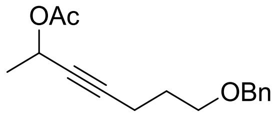

4f

This compound **4f** was prepared in 90% yield through the General Procedure A.^{1}H NMR (600 MHz, $CDCl_3$) δ 7.39 – 7.22 (m, 5H), 5.42 (dt, *J* = 8.6, 4.7Hz, 1H), 4.51 (s, 2H), 3.55 (t, *J* = 6.2 Hz, 2H), 2.34 (td, *J* = 7.1, 1.9 Hz, 2H), 2.06 (s, 3H), 1.86 – 1.74 (m, 2H), 1.44 (d, *J* = 6.6 Hz, 3H). ^{13}C NMR (151 MHz, $CDCl_3$) δ 169.95, 138.44, 128.33, 127.56, 127.52, 84.77, 78.92, 72.92, 68.66, 60.75, 28.59, 21.75, 21.14, 15.53. IR(neat): 2989, 2937, 2859, 1740, 1371, 1235, 1106, 1060, 1019, 944, 737. ESI ($M+Na^+$): 283.15.

4-Cyclohexylbut-3-yn-2-yl acetate (4g)

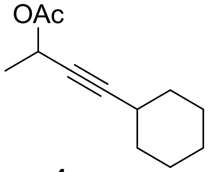

4g

This known compound **4g** was prepared in 90% yield through the General Procedure A and its spectroscopic data were in accordance with the literature data. [4] ^{1}H NMR (500 MHz, $CDCl_3$) δ 5.46 (qd, *J* = 6.6, 1.8 Hz, 1H), 2.47 – 2.33 (m, 1H), 2.06 (s, 3H), 1.82 – 1.73 (m, 2H), 1.69-1.67

(m, 2H), 1.54 – 1.36 (m, 3H), 1.45 (d, *J* = 6.6 Hz, 3H), 1.34 – 1.23 (m, 3H). ^{13}C NMR (126 MHz, $CDCl_3$) δ 169.96, 89.52, 78.54, 60.87, 32.45, 28.93, 25.82, 24.79, 21.94, 21.20.

4-(Benzyloxy)but-2-ynyl acetate (4h)

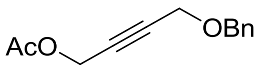

4h

This compound **4h** was prepared in 88% yield through the General Procedure A. ^{1}H NMR (500 MHz, $CDCl_3$) δ 7.41 – 7.26 (m, 5H), 4.74 (t, *J* = 1.8 Hz, 2H), 4.59 (s, 2H), 4.21 (t, *J* = 1.8 Hz, 2H), 2.11 (s, 3H). ^{13}C NMR (126 MHz, $CDCl_3$) δ 170.19, 137.22, 128.42, 128.05, 127.89, 82.74, 80.49, 71.70, 57.28, 52.25, 20.71. IR(neat): 3032, 2944, 2858, 1749, 1357, 1224, 1074, 1028, 741. ESI ($M+Na^+$):241.10.

5-(Benzyloxy)pent-3-yn-2-yl acetate (4i)

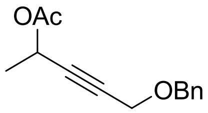

4i

This compound **4i** was prepared in 85% yield through the General Procedure A. ^{1}H NMR (500 MHz, $CDCl_3$) δ 7.40 – 7.27 (m, 5H), 5.50 (qt, *J* = 6.7, 1.6 Hz, 1H), 4.58 (s, 2H), 4.20 (d, *J* = 1.6 Hz, 2H), 2.08 (s, 3H), 1.51 (d, *J* = 6.7 Hz, 3H). ^{13}C NMR (126 MHz, $CDCl_3$) δ 169.87, 137.28, 128.41, 128.09, 127.87, 84.95, 80.66, 71.61, 60.30, 57.26, 21.32, 21.04. IR(neat): 2939, 1744, 1372, 1235, 1161, 1057, 1018, 741. ESI ($M+Na^+$):255.11.

4-Phenylbut-3-yn-2-yl acetate (4j)

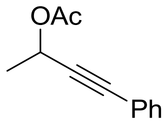

4j

This known compound **4j** was prepared in 95% yield through the General Procedure A and its spectroscopic data were in accordance with the literature data. [1] ^{1}H NMR (500 MHz, $CDCl_3$) δ 7.51 – 7.40 (m, 2H), 7.36 – 7.26 (m, 3H), 5.69 (q, *J* = 6.7 Hz, 1H), 2.11 (s, 3H), 1.58 (d, *J* = 6.7 Hz, 3H). ^{13}C NMR (126 MHz, $CDCl_3$) δ 131.84, 128.55, 128.21, 122.25, 87.39, 84.53, 60.79, 21.50, 21.10

4-Cyclohexenylbut-3-yn-2-yl acetate (4k)

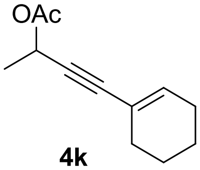

4k

This compound **4k** was prepared in 85% yield through the General Procedure A. ^{1}H NMR (600 MHz, $CDCl_3$) δ 6.16 – 6.09 (m, 1H), 5.57 (q, *J* = 6.6 Hz, 1H), 2.20 – 2.01 (m, 4H), 2.06 (s, 3H), 1.64 – 1.58 (m, 2H), 1.58 – 1.53 (m, 2H), 1.48 (d, *J* = 6.6 Hz, 3H). ^{13}C NMR (151 MHz, $CDCl_3$) δ 169.94, 135.98, 119.82, 86.37, 84.67, 60.94, 28.98, 25.59, 22.19, 21.66, 21.39, 21.16. IR(neat): 2937, 1745, 1372, 1235, 1213, 1083, 1029, 946. ESI ($M+Na^+$):215.11.

1-Cyclopropyloct-1-yn-3-yl acetate (4l)

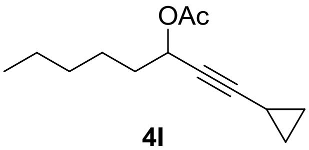

4l

This compound **4l** was prepared in 84% yield through the General Procedure A. ^{1}H NMR (600 MHz, $CDCl_3$) δ 5.30 (td, *J* = 6.7, 1.6 Hz, 1H), 2.04 (s, 3H), 1.75 – 1.59 (m, 2H), 1.38 (p, *J* = 7.6 Hz, 2H), 1.33 – 1.16 (m, 5H), 0.87 (t, *J* = 7.0 Hz, 3H), 0.79 – 0.71 (m, 2H), 0.70 – 0.61 (m, 2H). ^{13}C NMR (151 MHz, $CDCl_3$) δ 170.09, 89.16, 72.88, 64.60, 35.05, 31.26, 24.68, 22.45, 21.14, 13.94, 8.29, -0.59. IR(neat): 2957, 2933, 1744, 1371, 1236, 1019, 947, 890. ESI ($M+Na^+$):231.15.

1-(4-(Trifluoromethyl)phenyl)hept-2-ynyl acetate (4m)

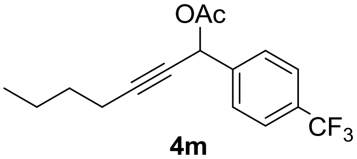

4m

This known compound **4m** was prepared in 86% yield through the General Procedure A and its spectroscopic data were in accordance with the literature data. [3] ^{1}H NMR (600 MHz, $CDCl_3$) δ 7.63 (s, 4H), 6.48 (d, *J* = 1.8 Hz, 1H), 2.27 (td, *J* = 7.2, 2.0 Hz, 2H), 2.11 (s, 3H), 1.60 – 1.47 (m, 2H), 1.41 (dt, *J* = 14.8, 7.3 Hz, 2H), 0.91 (t, *J* = 7.3 Hz, 3H). ^{13}C NMR (151 MHz, $CDCl_3$) δ 169.70, 141.55, 127.93, 125.56, 125.53, 89.16, 76.00, 65.29, 30.38, 21.95, 21.06, 18.49, 13.54.

The General Procedure B: Gold-catalyzed oxidation/acetoxy migration reaction of propargyl acetates to α-acetoxyenones

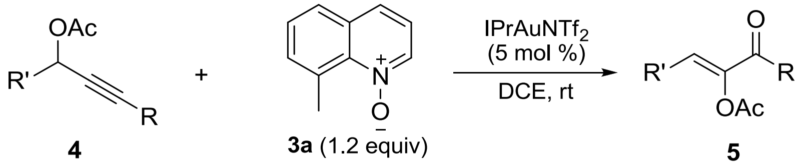

8-methylquinoline-*N*-oxide **3a** (0.36 mmol, 1.2 equiv) and IPrAuNTf2 (13.1 mg, 0.015 mmol, 5 mol %) were added in this order to a solution of the propargyl acetates **4** (0.3 mmol) in DCE (6 mL) at room temperature. The reaction mixture was stirred at the same temperature until the propargyl acetates was completely consumed. The reaction mixture was concentrated under vacuum. The residue was purified by chromatography on silica gel (eluent: hexanes/ethyl acetate) to afford the desired α-acetoxyenones **5**.

(Z)-4-Oxooct-2-en-3-yl acetate (5a)

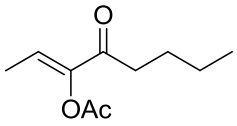

5a

The compound **5a** was prepared in 82 % yield according to the General Procedure B (eluents: ethyl acetate: hexanes = 1: 10).^{1}H NMR (500 MHz, $CDCl_3$) δ 6.53 (q, *J* = 7.0 Hz, 1H), 2.59 (t, *J* = 7.4 Hz, 2H), 2.24(s, 3H), 1.77 (d, *J* = 7.0 Hz, 3H), 1.58 (p, *J* = 7.4 Hz, 2H), 1.31 (h, *J* = 7.4 Hz, 2H), 0.89 (t, *J* = 7.4 Hz, 3H). ^{13}C NMR (126 MHz, $CDCl_3$) δ 193.71, 168.52, 147.05, 126.84, 36.76, 26.20, 22.24, 20.22, 13.79, 11.69. IR(neat):2961, 2875, 1764, 1688, 1371, 1207, 1033. ESI ($M+Na^+$):207.09.

3-Oxohex-1-en-2-yl acetate (5b)

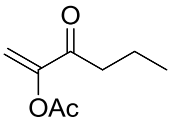

5b

The compound **5b** was prepared in 80 % yield according to the General Procedure B but with the exception that Me_4*t*-BuXPhosAuNTf$_2$ (5 mol %) was used as the catalyst using as the catalyst

(eluents: ethyl acetate: hexanes = 1: 10).^{1}H NMR (500 MHz, $CDCl_3$) δ 5.90 (d, *J* = 2.4 Hz, 1H), 5.58 (d, *J* = 2.4 Hz, 1H), 2.64 (t, *J* = 7.3 Hz, 2H), 2.23 (s, 3H), 1.67 (h, *J* = 7.4 Hz, 2H), 0.95 (t, *J* = 7.4 Hz, 3H). ^{13}C NMR (126 MHz, $CDCl_3$) δ 194.14, 168.89, 151.63, 113.05, 39.39, 20.42, 17.39, 13.61. IR(neat): 2967, 1729, 1687, 1374, 1207, 1044, 750. ESI ($M+H^+$):157.08.

(Z)-3-Oxo-1-phenylprop-1-en-2-yl pivalate (5c)

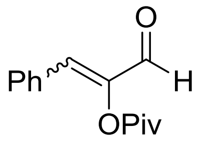

5c (Z/E= 9/1)

By following the General Procedure B except using the combination of IPrAuCl and $AgSbF_6$ (5 mol % each) as the catalyst, the known compound, (*Z*)-**5c**, was isolated in 67% yield along with 7% of the (*E*)-isomer and its spectroscopic data were in accordance with the literature data.[2] ^{1}H NMR (500 MHz, $CDCl_3$) δ 9.40 (s, 1H), 7.69 – 7.60 (m, 2H), 7.46 – 7.31 (m, 3H), 7.01 (s, 1H), 1.41 (s, 9H). ^{13}C NMR (126 MHz, $CDCl_3$) δ 185.44, 175.25, 146.39, 136.39, 131.73, 130.69, 130.31, 128.80, 39.08, 27.12.

(Z)-5-Oxododec-6-en-6-yl acetate (5d)

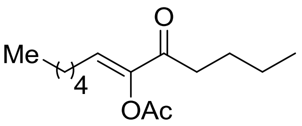

5d

The compound **5d** was prepared in 86% yield according to the General Procedure B (eluents: ethyl acetate: hexanes = 1: 10).^{1}H NMR (500 MHz, $CDCl_3$) δ 6.43 (t, *J* = 7.5 Hz, 1H), 2.59 (t, *J* = 7.4 Hz, 2H), 2.23 (s, 3H), 2.15 (q, *J* = 7.5 Hz, 2H), 1.67 – 1.52 (m, 2H), 1.44 (p, *J* = 7.3 Hz, 2H), 1.37 – 1.24 (m, 6H), 0.98 – 0.74 (m, 6H). ^{13}C NMR (126 MHz, $CDCl_3$) δ 193.89, 168.61, 146.05, 131.94, 36.79, 31.39, 27.81, 26.24, 26.07, 22.29, 22.24, 20.24, 13.85, 13.79. IR(neat): 2933, 2959, 1766, 1688, 1370, 1203, 1032, 784. ESI ($M+Na^+$):263.16.

(Z)-5-Oxo-1-phenylnon-3-en-4-yl acetate (5e)

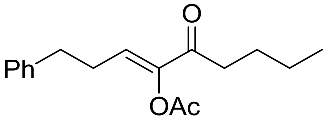

5e

The compound **5e** was prepared in 85% yield according to the General Procedure B (eluents: ethyl acetate: hexanes = 1: 10). ^{1}H NMR (500 MHz, $CDCl_3$) δ 7.31 (t, *J* = 7.6 Hz, 2H), 7.24-7.19 (m, 3H), 6.45 (t, *J* = 7.4 Hz, 1H), 2.78 (t, *J* = 7.7 Hz, 2H), 2.58 (t, *J* = 7.4 Hz, 2H), 2.53 – 2.48 (m, 2H), 2.25 (s, 3H), 1.59 (p, *J* = 7.5 Hz, 2H), 1.33 (h, *J* = 7.4 Hz, 2H), 0.91 (t, *J* = 7.4 Hz, 3H). ^{13}C NMR (126 MHz, $CDCl_3$) δ 193.87, 168.55, 146.25, 140.44, 130.47, 128.49, 128.23, 126.28, 36.82, 34.20, 27.77, 26.26, 22.21, 20.24, 13.78. IR(neat): 2933, 2872, 1763, 1687, 1371, 1204, 1031. ESI ($M+Na^+$): 297.15.

(Z)-7-(Benzyloxy)-4-oxohept-2-en-3-yl acetate (5f)

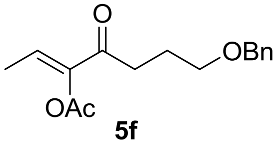

5f

The compound **5f** was prepared in 75% yield according to the General Procedure B with the exception that *t*-BuS-PhosAuCl/$AgNTf_2$ (10 mol %) was used as the catalyst (eluents: ethyl acetate: hexanes = 1: 10). ^{1}H NMR (500 MHz, $CDCl_3$) δ 7.38 – 7.24 (m, 5H), 6.56 (q, *J* = 7.1 Hz, 1H), 4.48 (s, 2H), 3.50 (t, *J* = 6.1 Hz, 2H), 2.74 (t, *J* = 7.2 Hz, 2H), 2.26 (s, 3H), 1.99 – 1.88 (m, 2H), 1.77 (d, *J* = 7.1 Hz, 3H). ^{13}C NMR (126 MHz, $CDCl_3$) δ 193.29, 168.50, 146.96, 138.37, 128.28, 127.55, 127.47, 127.16, 72.78, 69.06, 33.63, 24.14, 20.20, 11.69. IR(neat): 3346, 2859, 1760, 1683, 1369, 1201, 1027, 805, 735. ESI ($M+Na^+$):299.12.

(Z)-1-Cyclohexyl-1-oxobut-2-en-2-yl acetate (5g)

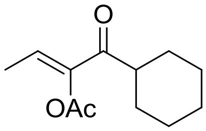

5g

The compound **5g** was prepared in 76% yield according to the General Procedure B (eluents: ethyl acetate: hexanes = 1: 10). ^{1}H NMR (500 MHz, $CDCl_3$) δ 6.54 (q, *J* = 7.1 Hz, 1H), 2.81 (tt, *J* = 11.5, 3.1 Hz, 1H), 2.25 (s, 3H), 1.86 – 1.73 (m, 4H),1.79 (d, *J* = 7.0 Hz, 3H), 1.73 – 1.62 (m, 1H), 1.48 – 1.35 (m, 2H), 1.33 – 1.14 (m, 3H). ^{13}C NMR (126 MHz, $CDCl_3$) δ 197.03, 168.54, 146.34, 126.58, 44.86, 29.28, 25.75, 25.70, 20.27, 11.77. IR(neat): 2934, 2857, 1764, 1683, 1370, 1204, 1018, 974. ESI ($M+Na^+$):233.11.

4-(Benzyloxy)-3-oxobut-1-en-2-yl acetate (5h)

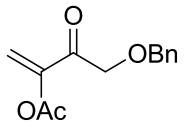

5h

The compound **5h** was prepared in 62% yield according to the General Procedure B with the exception that Me_4*t*BuXPhosAuNTf_2 (5 mol %) was used as the catalyst (eluents: ethyl acetate: hexanes = 1: 10). ^{1}H NMR (500 MHz, $CDCl_3$) δ 7.41 – 7.27 (m, 5H), 5.95 (d, *J* = 2.5 Hz, 1H), 5.63 (d, *J* = 2.5 Hz, 1H), 4.61 (s, 2H), 4.43 (s, 2H), 2.23 (s, 3H). ^{13}C NMR (126 MHz, $CDCl_3$) δ 191.11, 168.71, 149.51, 136.95, 128.46, 128.03, 128.01, 114.07, 73.26, 71.79, 20.34. IR(neat): 3089, 3033, 2869, 1766, 1715, 1372, 1208, 1028, 929,749. ESI ($M+Na^+$):257.07.

(Z)-5-(Benzyloxy)-4-oxopent-2-en-3-yl acetate (5i)

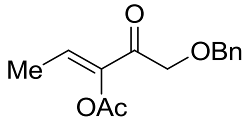

5i

The compound **5i** was prepared in 60% yield according to the General Procedure B with the exception that t-BuS-PhosAuCl/$AgNTf_2$ (5 mol %) was used as the catalyst (eluents: ethyl acetate: hexanes = 1: 10). ^{1}H NMR (600 MHz, $CDCl_3$) δ 7.36-7.26 (m, 5H), 6.60 (q, *J* = 7.1 Hz, 1H), 4.59 (s, 2H), 4.38 (s, 2H), 2.26 (s, 3H), 1.78 (d, *J* = 7.1 Hz, 3H). ^{13}C NMR (151 MHz, $CDCl_3$) δ 190.35, 168.45, 145.22, 137.10, 128.61, 128.46, 128.07, 127.98, 73.23, 71.64, 20.22, 11.72. IR(neat): 2924, 1762, 1703, 1370, 1205, 1028, 735. ESI ($M+Na^+$):271.09.

(Z)-1-Oxo-1-phenylbut-2-en-2-yl acetate (5j)

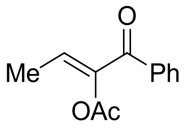

5j

The compound **5j** was prepared in 75% yield according to the General Procedure B with the exception that *t*-BuS-PhosAuCl/$AgNTf_2$ (5 mol %) was used as the catalyst (eluents: ethyl acetate: hexanes = 1: 10). ^{1}H NMR (600 MHz, $CDCl_3$) δ 7.75 (d, *J* = 7.5 Hz, 2H), 7.54 (t, *J* = 7.2 Hz, 1H), 7.43 (t, *J* = 7.5 Hz, 2H), 6.24 (q, *J* = 6.9 Hz, 1H), 2.27 (s, 3H), 1.85 (d, *J* = 7.0 Hz, 3H). ^{13}C NMR (151 MHz, $CDCl_3$) δ 189.65, 168.56, 146.63, 136.91, 132.19, 129.90, 129.17, 128.16, 20.31, 11.80. IR(neat): 3065, 2938, 1760, 1664, 1371, 1275, 1020, 846, 777, 709. ESI ($M+Na^+$):227.06.

(Z)-1-Cyclohexenyl-1-oxobut-2-en-2-yl acetate (5k)

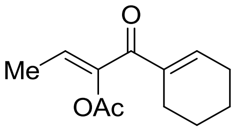

5k

The compound **5k** was prepared in 90% yield according to the General Procedure B (eluents: ethyl acetate: hexanes = 1: 10). ^{1}H NMR (500 MHz, $CDCl_3$) δ 6.73 – 6.67 (m, 1H), 6.14 (qd, *J* = 7.0, 0.7 Hz, 1H), 2.30 – 2.22 (m, 4H), 2.22 (s, 3H), 1.77 (d, *J* = 7.1 Hz, 3H), 1.66-1.60 (m, 4 H). ^{13}C NMR (126 MHz, $CDCl_3$) δ 190.70, 168.65, 146.20, 140.43, 137.43, 126.51, 25.72, 23.88, 21.83, 21.52, 20.32, 11.48. IR(neat): 3416, 2938, 1760, 1648, 1371, 1210, 1019, 930, 833, 777. ESI ($M+Na^+$): 231.10.

(Z)-1-Cyclopropyl-1-oxooct-2-en-2-yl acetate (5l)

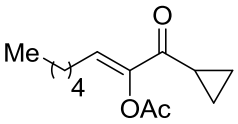

5l

The compound **5l** was prepared in 84% yield according to the General Procedure B (eluents: ethyl acetate: hexanes = 1: 10). ^{1}H NMR (500 MHz, $CDCl_3$) δ 6.57 (t, *J* = 7.5 Hz, 1H), 2.29 – 2.24 (m, 1H), 2.23 (s, 3H), 2.19 (q, *J* = 7.5 Hz, 2H), 1.51 – 1.42 (m, 2H), 1.34 – 1.27 (m, 4H),

1.11 – 1.06 (m, 2H), 0.95 – 0.90 (m, 2H), 0.88 (t, J = 7.4 Hz, 3H). ^{13}C NMR (126 MHz, $CDCl_3$) δ 193.62, 168.58, 146.49, 131.66, 31.39, 27.85, 26.14, 22.29, 20.23, 15.99, 13.83, 11.25. IR(neat): 3332, 2931, 1765, 1674, 1370, 1206, 1021, 916. ESI ($M+Na^+$):247.12.

(Z)-3-Oxo-1-(4-(trifluoromethyl)phenyl)hept-1-en-2-yl acetate (5m)

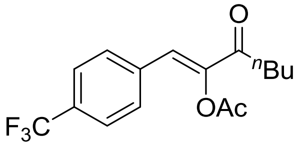

5m

The compound **5m** was prepared in 85% yield according to the General Procedure B (eluents: ethyl acetate: hexanes = 1: 10). ^{1}H NMR (600 MHz, $CDCl_3$) δ 7.69 (d, J = 8.3 Hz, 2H), 7.64 (d, J = 8.4 Hz, 2H), 7.17 (s, 1H), 2.75 (t, J = 7.4 Hz, 2H), 2.33 (s, 3H), 1.68 (p, J = 7.5 Hz, 2H), 1.38 (h, J = 7.4 Hz, 2H), 0.94 (t, J = 7.4 Hz, 3H). ^{13}C NMR (151 MHz, $CDCl_3$) δ 194.45, 168.29, 146.01, 135.58, 131.36, 131.14, 130.12, 125.67, 125.64, 125.62, 125.59, 124.95, 124.62, 37.07, 26.13, 22.25, 20.60, 13.82. IR(neat): 2961, 2875, 1767, 1688, 1373, 1325, 1188, 1069, 884, 838, 760. ESI ($M+Na^+$): 337.10.

References:

1. S. Wang, L. Zhang, *J. Am. Chem. Soc.* **2006** , *128*, 8414 – 8415.
2. C. A. Witham, P. Mauleón, N. D. Shapiro, B. D. Sherry, F. D. Toste, *J. Am. Chem. Soc.* **2007**, *129*, 5838-5839.
3. T. L. Macdonald, D. R. Reagan, *J. Org. Chem.* **1980**, *45*, 4740 – 4747.
4. M. Yu, G. Zhang, L. Zhang, *Org. Lett*, **2007**, *9*, 2147 – 2150.

Data File Name C:/ Users/ zhanglab1/ Desktop/ NMR/ jikg/ CASE-SM/ jkg-II-224B-P-H.fid/ fid
Title jkg-II-224B-P-H
Solvent CDCl3
Acquisition Date 2012-02-27T13:20:12
Spectrometer Frequency 499.86

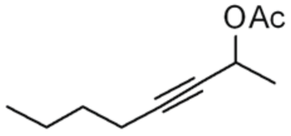

4a

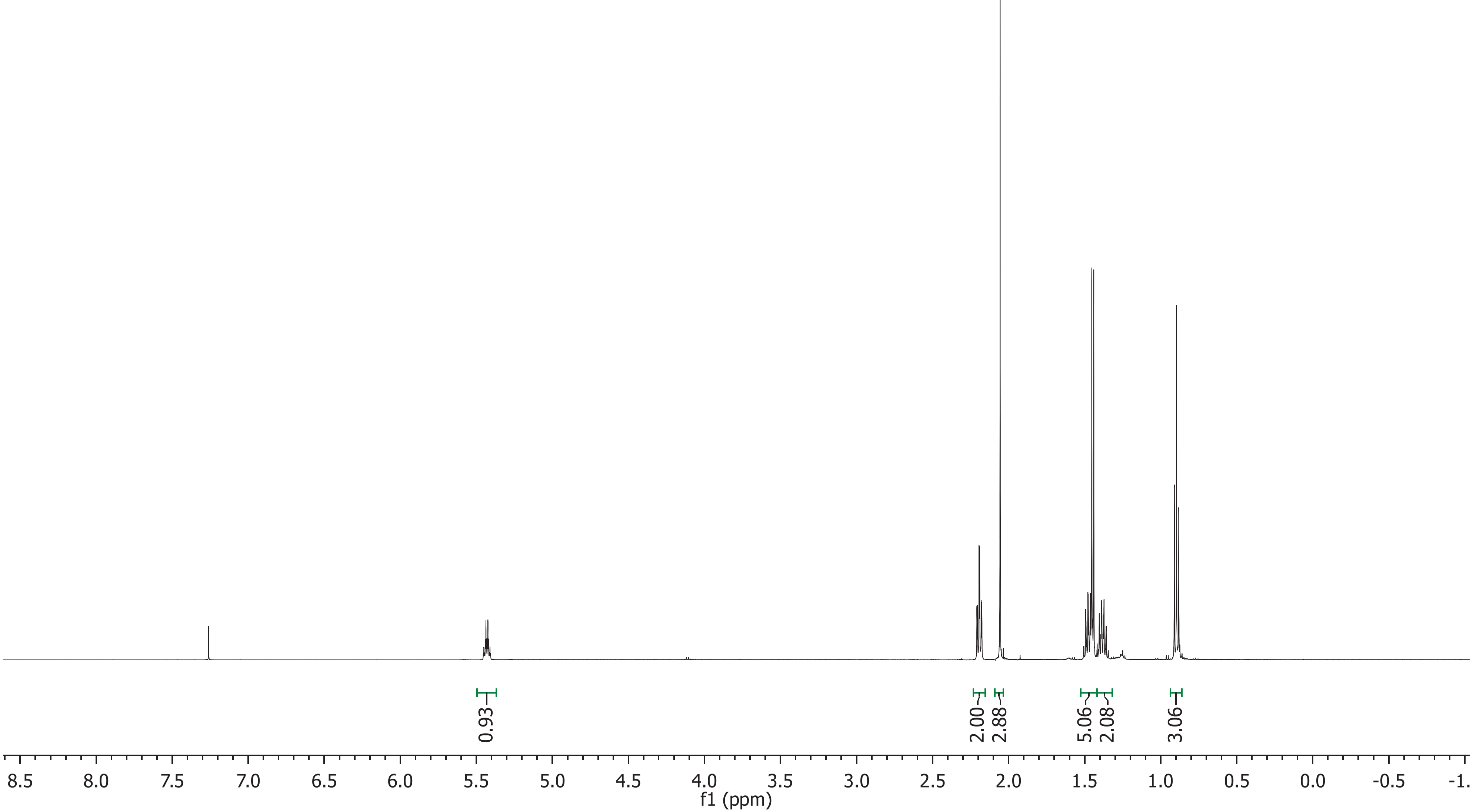

Data File Name	C:/ Users/ zhanglab1/ Desktop/ NMR/ jikg/ CASE-SM/ jkg-II-224B-P-C.fid/ fid
Title	jkg-II-224B-P-C
Solvent	CDCl3
Acquisition Date	2012-02-27T13:22:14
Spectrometer Frequency	125.70

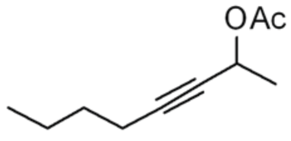

4a

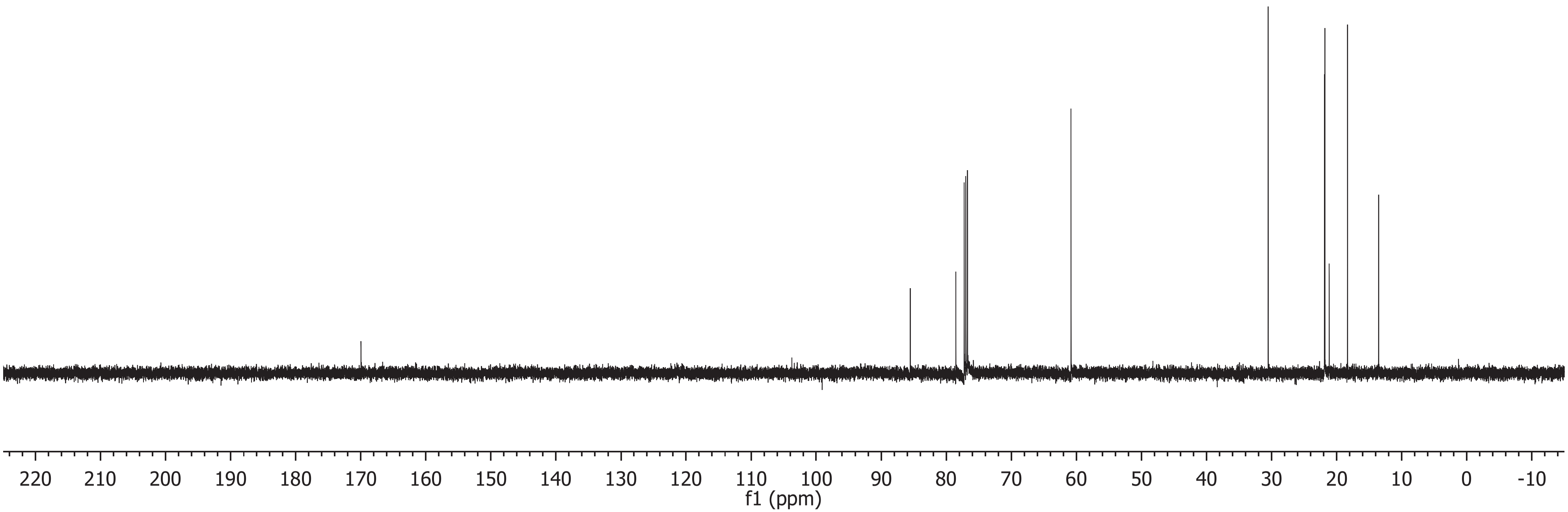

Data File Name C:/ Users/ zhanglab1/ Desktop/ NMR/ jikg/ CASE-SM/ jkg-II-226-SM-H.fid/ fid
Title jkg-II-226-SM-H
Solvent CDCl3
Acquisition Date 2012-02-27T17:10:33
Spectrometer Frequency 499.86

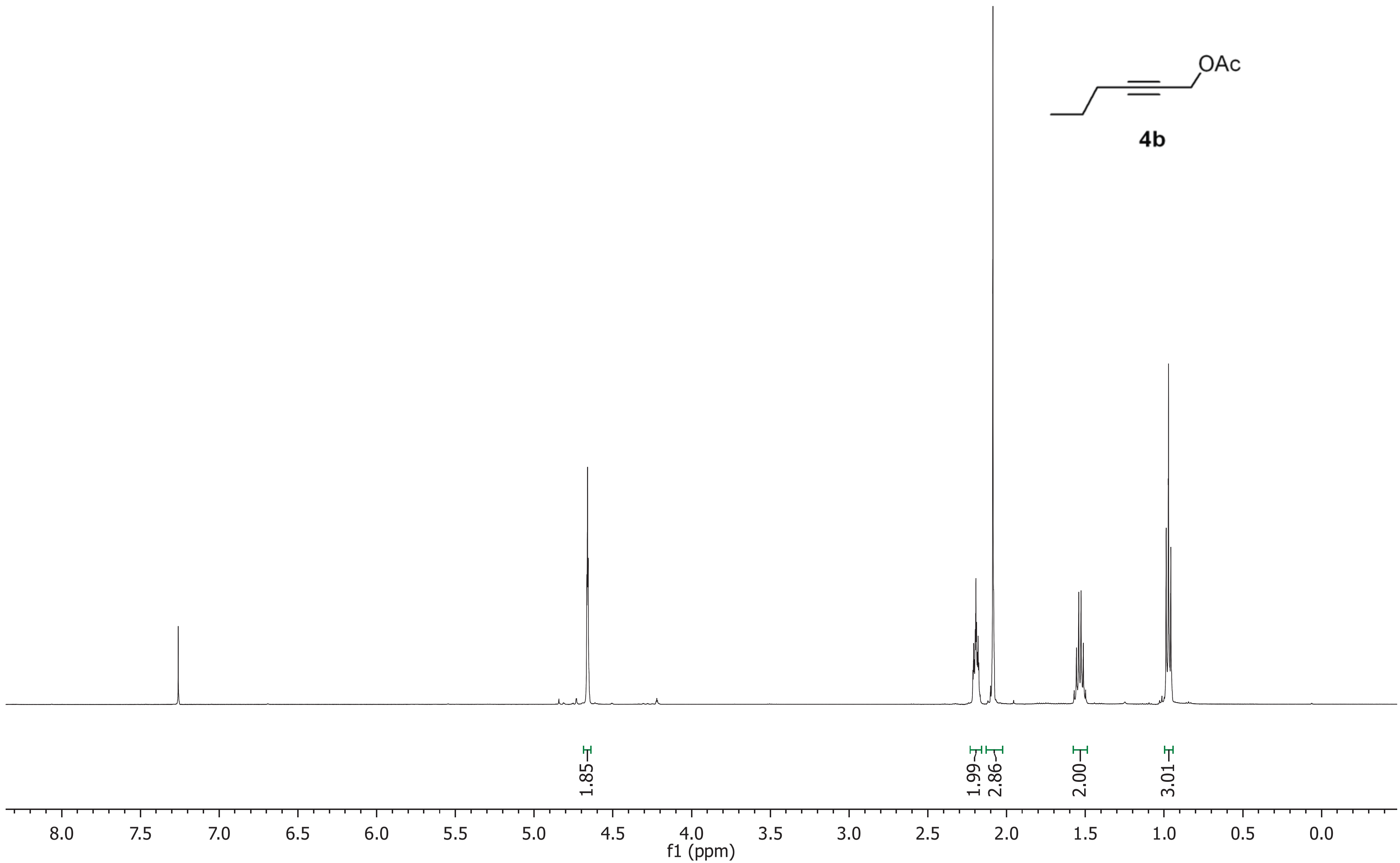

Data File Name	C:/ Users/ zhanglab1/ Desktop/ NMR/ jikg/ product/ jkg-III-69-SM-H.fid/ fid
Title	jkg-III-69-SM-H
Solvent	cdcl3
Acquisition Date	2012-05-15T16:59:29
Spectrometer Frequency	599.63

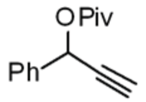

4c

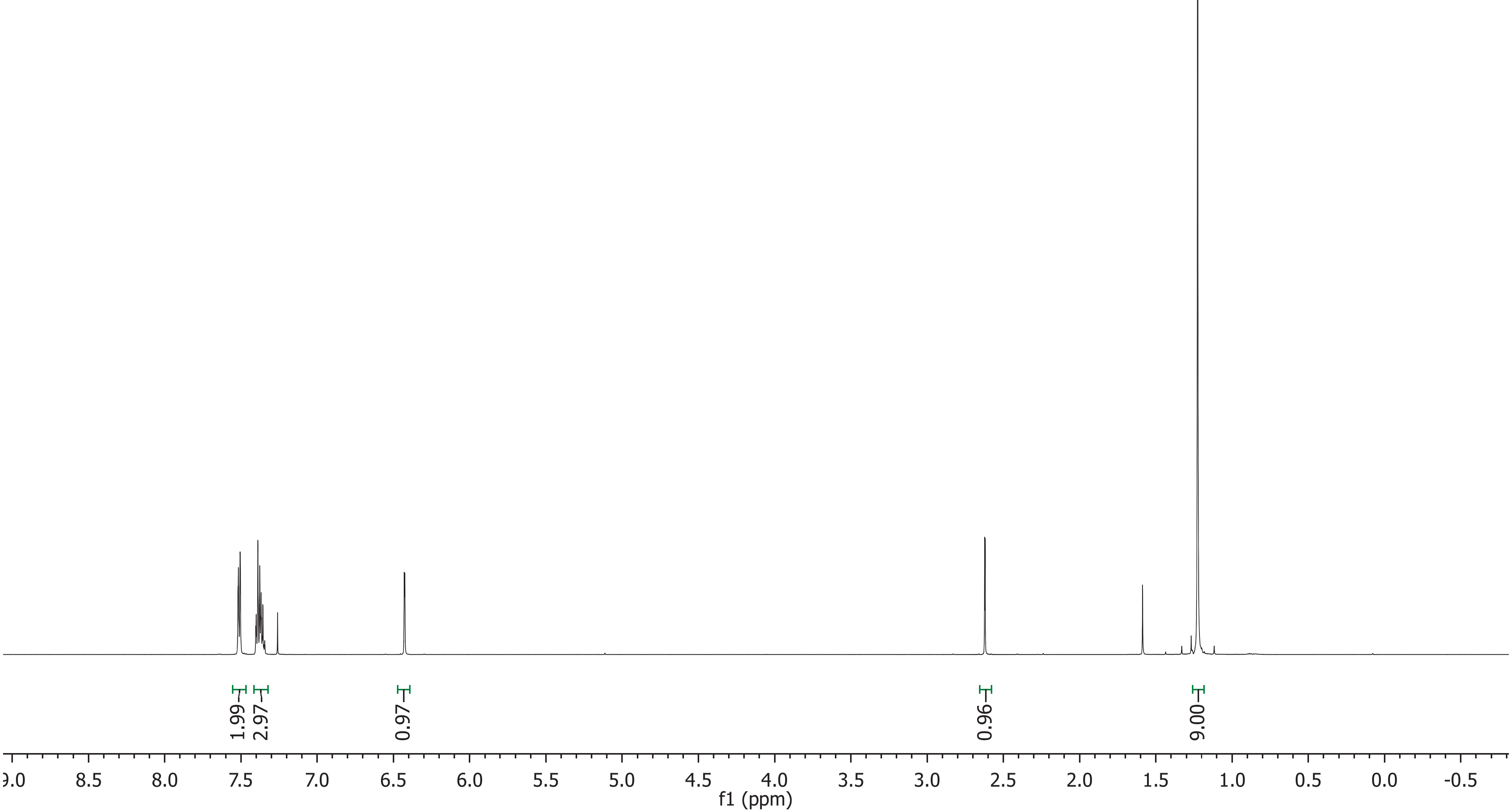

Data File Name	C:/ Users/ zhanglab1/ Desktop/ NMR/ jikg/ product/ jkg-III-69-SM-C.fid/ fid
Title	jkg-III-69-SM-C
Solvent	cdcl3
Acquisition Date	2012-05-15T17:02:18
Spectrometer Frequency	150.79

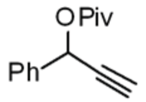

4c

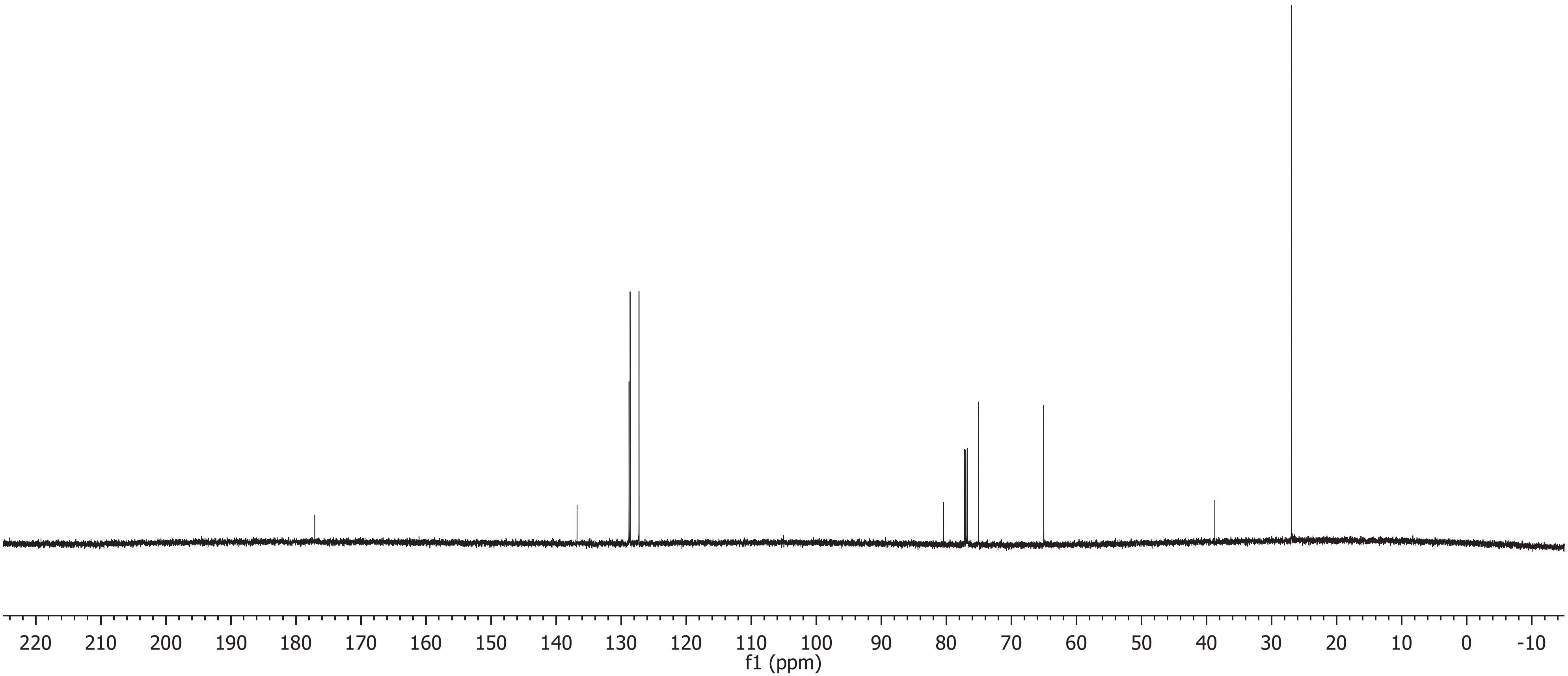

Data File Name	C:/ Users/ zhanglab1/ Desktop/ NMR/ jikg/ CASE-SM/ jkg-II-226-SM-C.fid/ fid
Title	jkg-II-226-SM-C
Solvent	CDCl3
Acquisition Date	2012-02-27T17:13:07
Spectrometer Frequency	125.70

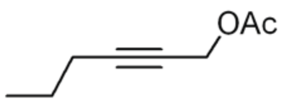

4b

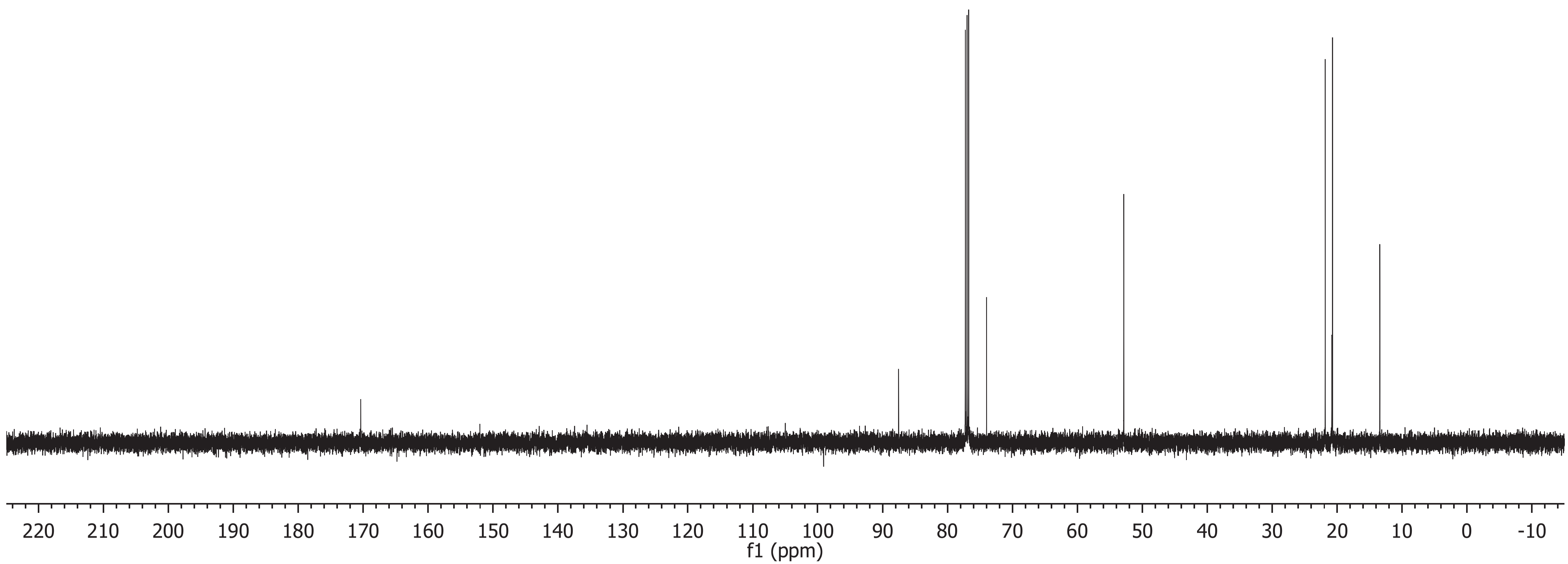

Data File Name C:/ Users/ zhanglab1/ Desktop/ NMR/ jikg/ CASE-SM/ jkg-II-234-SM-H.fid/ fid
Title jkg-II-234-SM-H
Solvent cdcl3
Acquisition Date 2012-04-22T16:11:28
Spectrometer Frequency 599.63

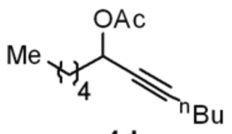

4d

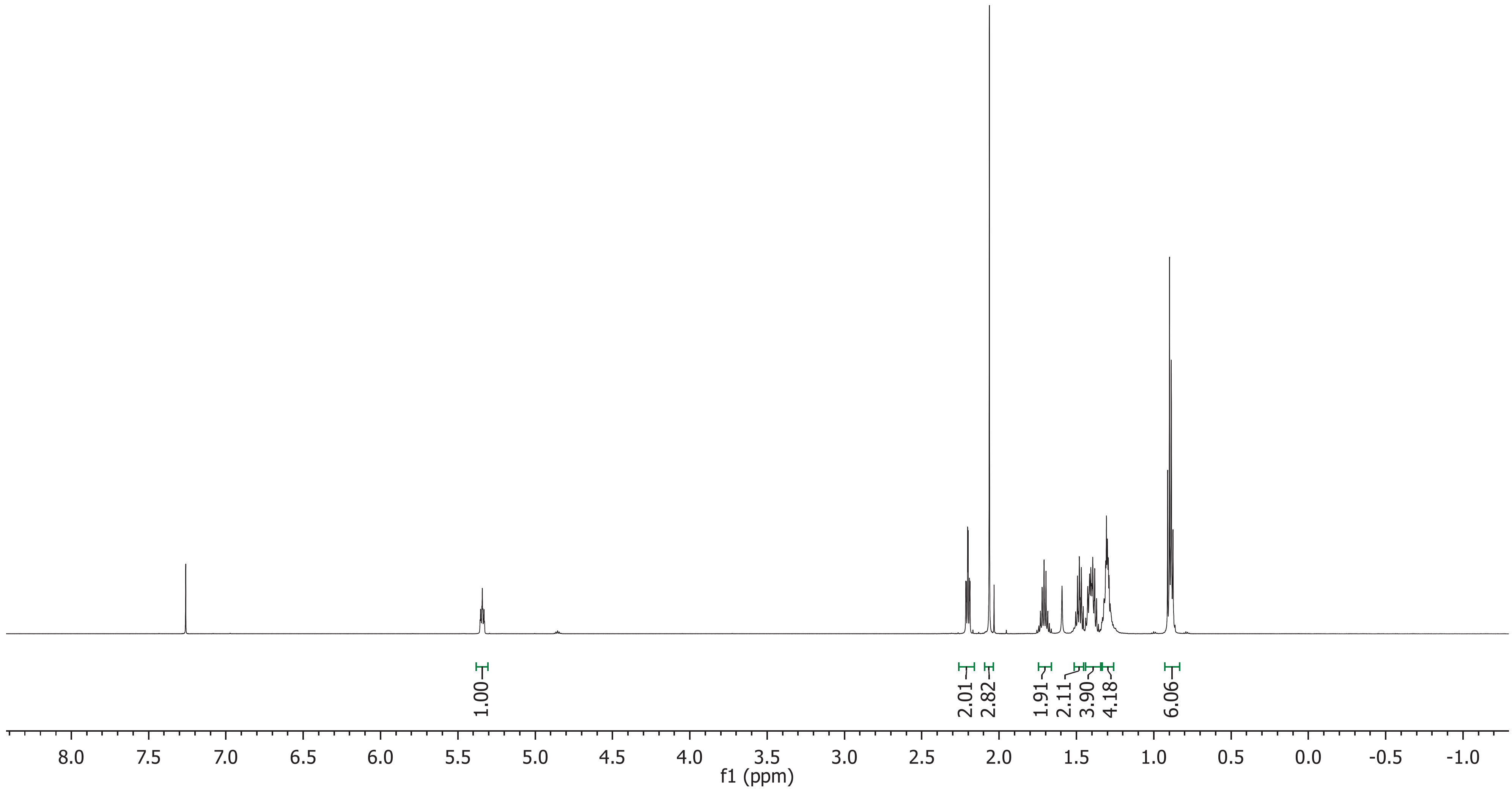

Data File Name	C:/ Users/ zhanglab1/ Desktop/ NMR/ jikg/ CASE-SM/ jkg-II-234-SM-C.fid/ fid
Title	jkg-II-234-SM-C
Solvent	cdcl3
Acquisition Date	2012-04-22T16:14:47
Spectrometer Frequency	150.79

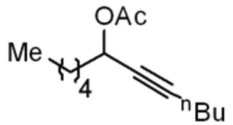

4d

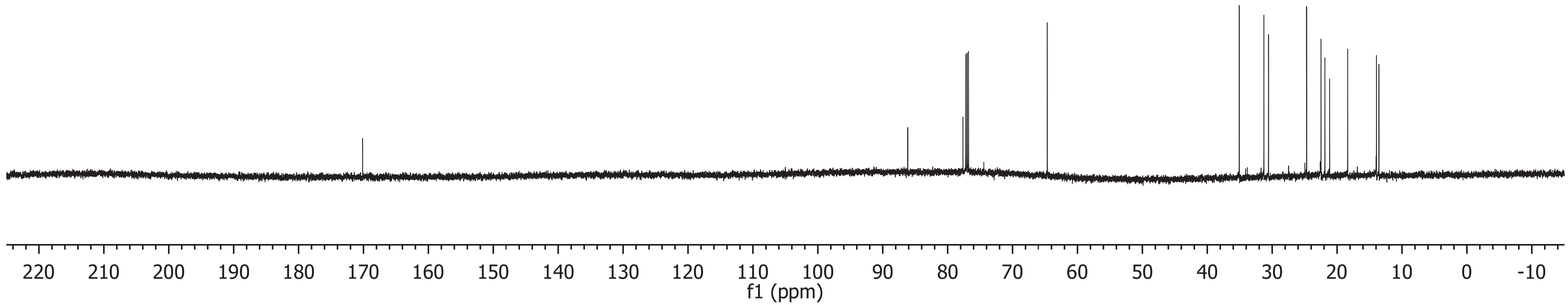

Data File Name	C:/ Users/ zhanglab1/ Desktop/ NMR/ jikg/ CASE-SM/ jkg-II-243B-1-P-H.fid/ fid
Title	jkg-II-243B-1-P-H
Solvent	CDCl3
Acquisition Date	2012-03-06T10:16:37
Spectrometer Frequency	499.86

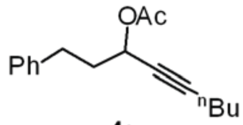

4e

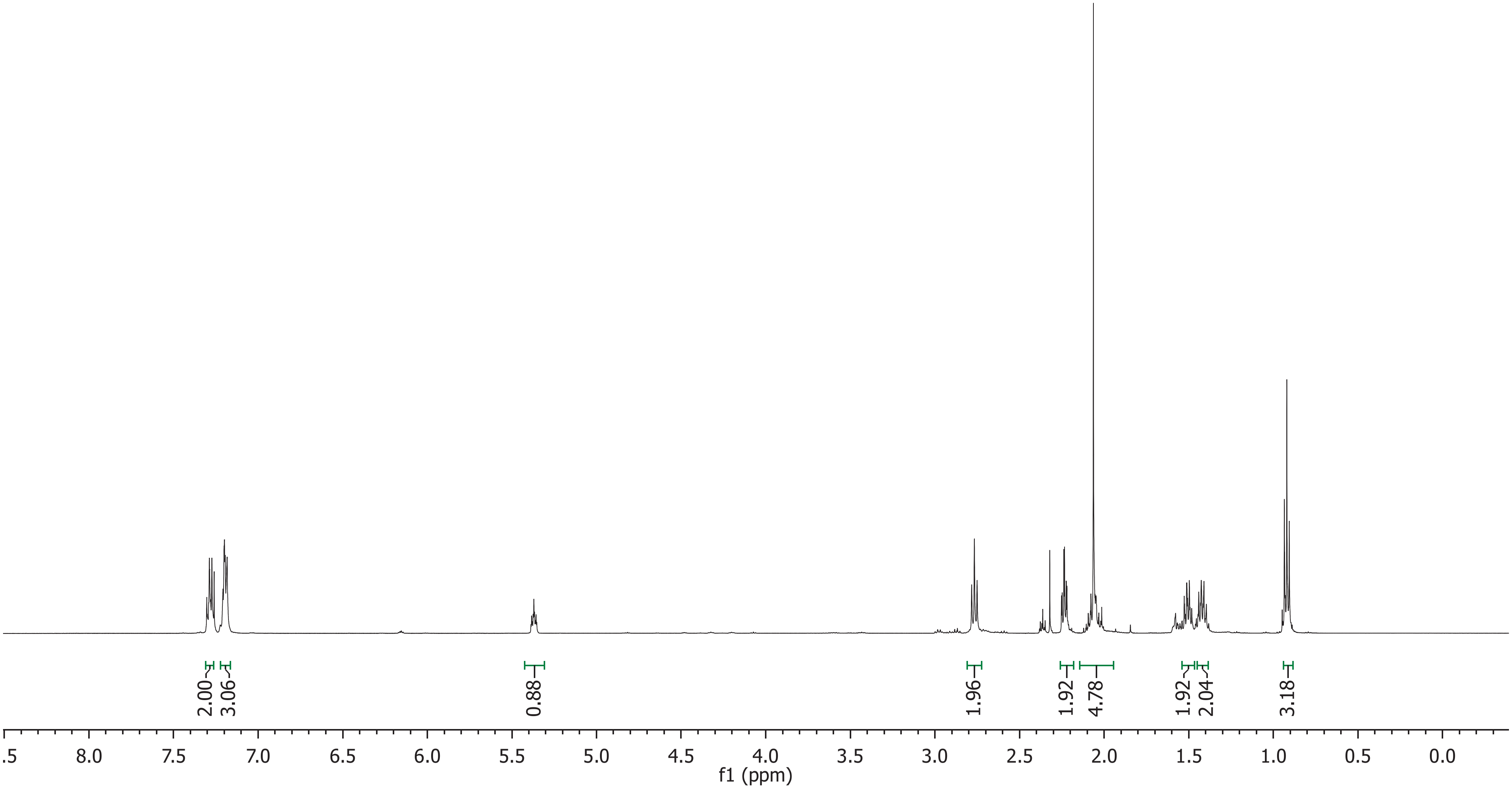

Data File Name	C:/ Users/ zhanglab1/ Desktop/ NMR/ jikg/ CASE-SM/ jkg-II-243B-1-P-C.fid/ fid
Title	jkg-II-243B-1-P-C
Solvent	CDCl3
Acquisition Date	2012-03-06T10:19:02
Spectrometer Frequency	125.70

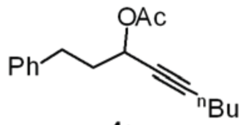

4e

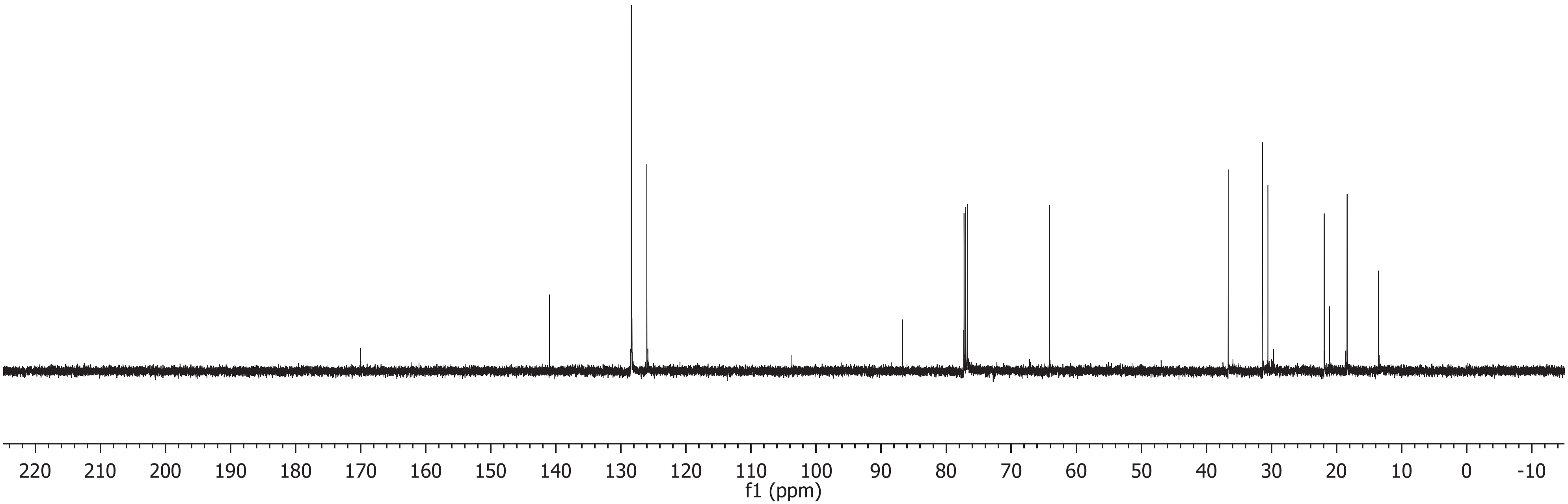

Data File Name	C:/ Users/ zhanglab1/ Desktop/ NMR/ jikg/ CASE-SM/ jkg-II-243-B-2-P-H.fid/ fid
Title	jkg-II-243-B-2-P-H
Solvent	cdcl3
Acquisition Date	2012-03-05T22:20:56
Spectrometer Frequency	599.63

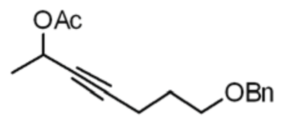

4f

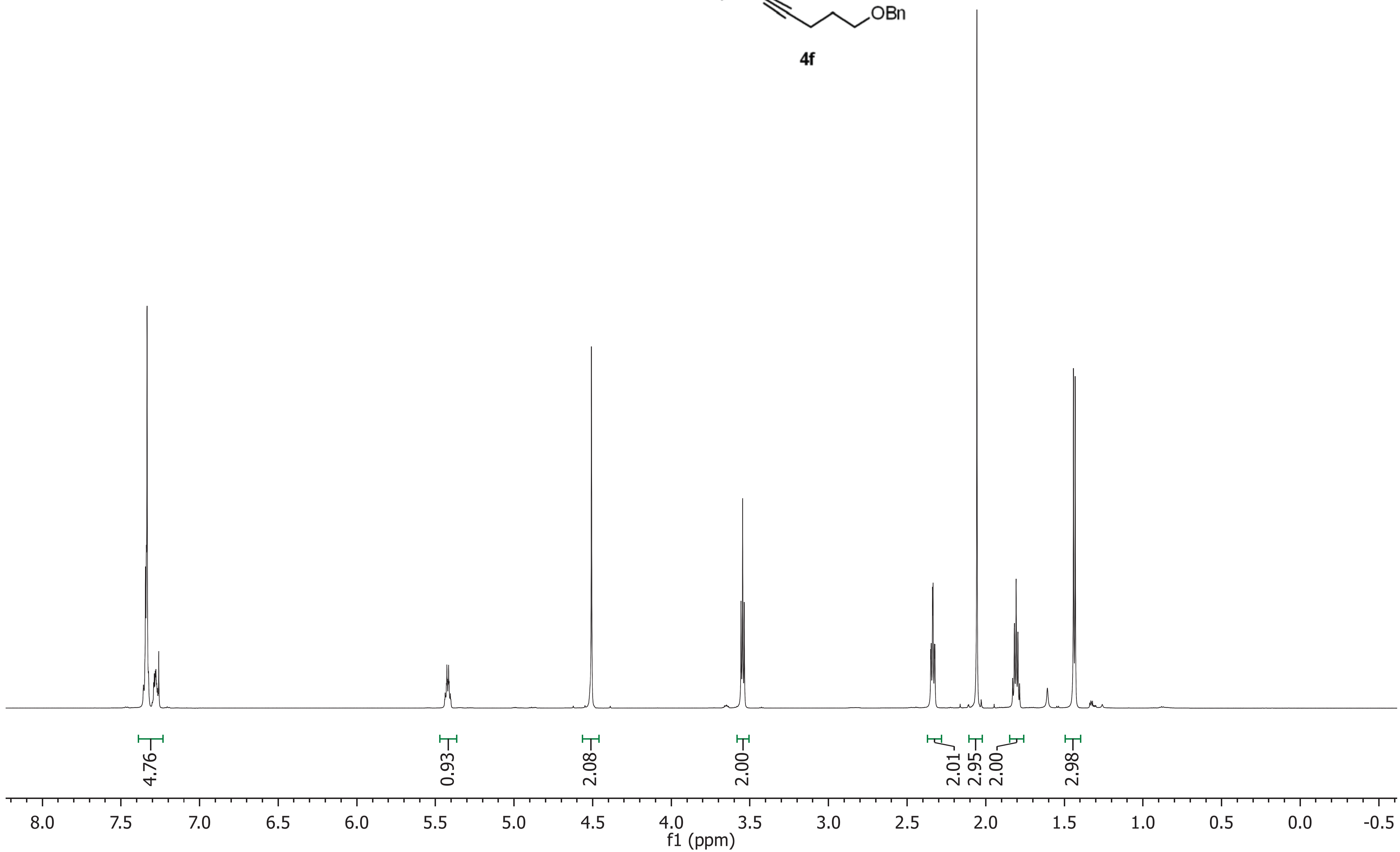

Data File Name	C:/ Users/ zhanglab1/ Desktop/ NMR/ jikg/ CASE-SM/ jkg-II-243-B-2-P-C.fid/ fid
Title	jkg-II-243-B-2-P-C
Solvent	cdcl3
Acquisition Date	2012-03-05T22:23:24
Spectrometer Frequency	150.79

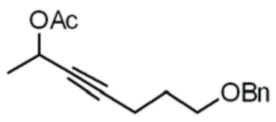

4f

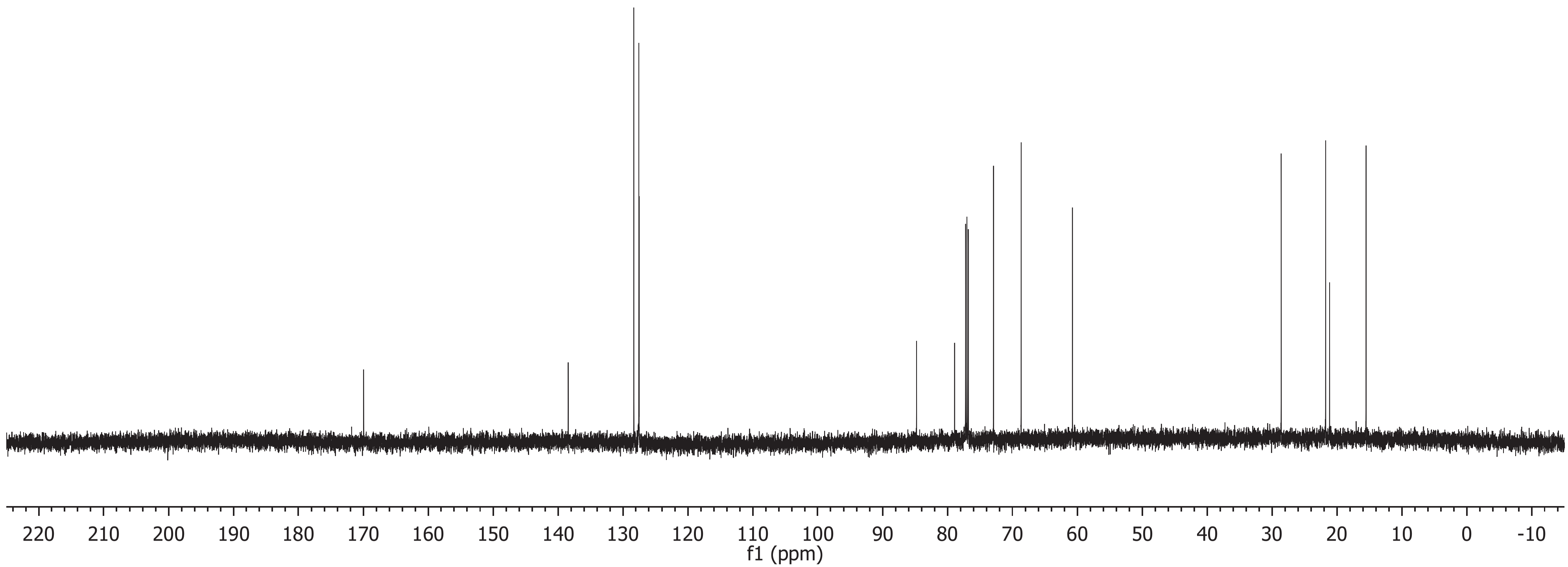

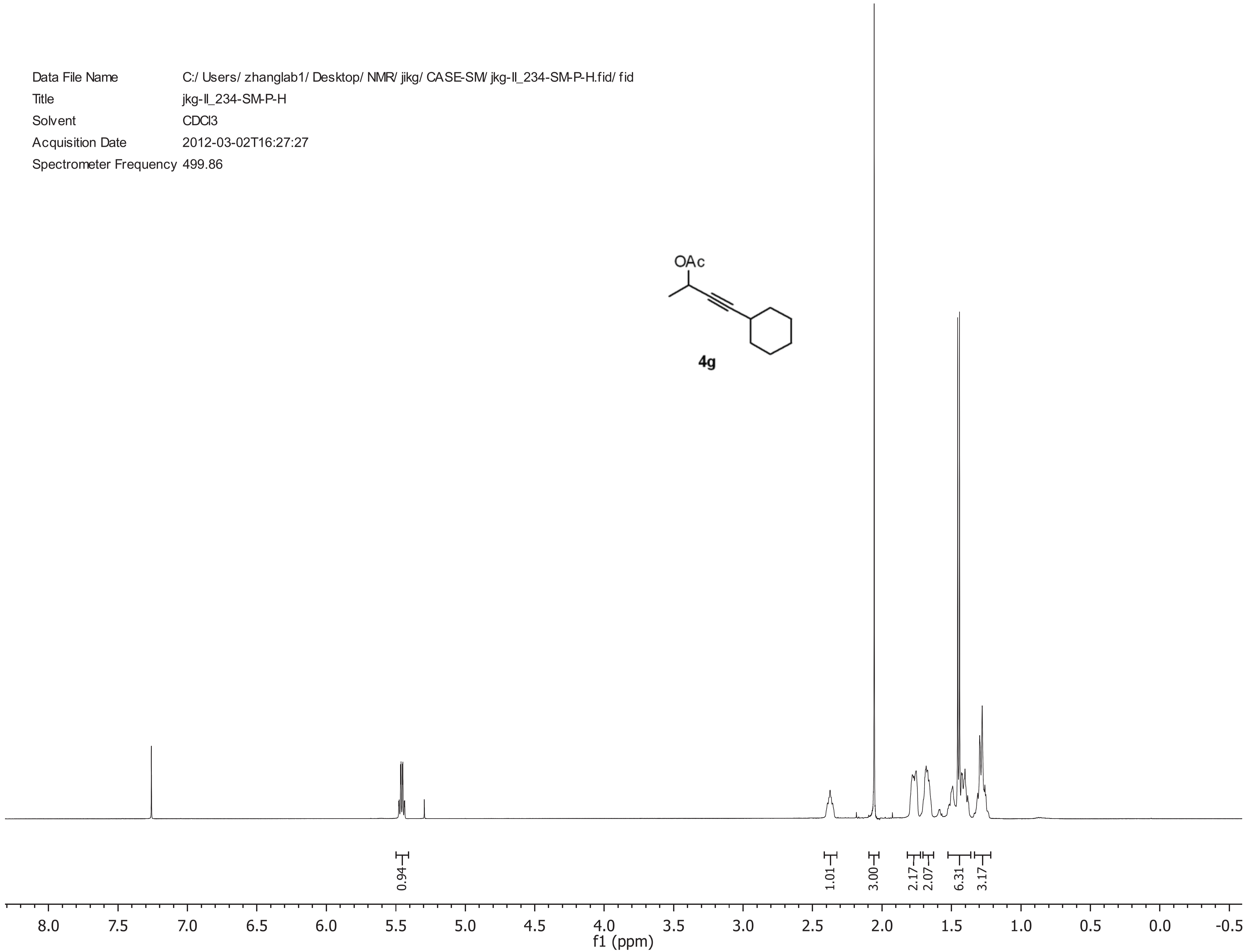
Data File Name C:/ Users/ zhanglab1/ Desktop/ NMR/ jikg/ CASE-SM/ jkg-II_234-SM-P-H.fid/ fid
Title jkg-II_234-SM-P-H
Solvent CDCl3
Acquisition Date 2012-03-02T16:27:27
Spectrometer Frequency 499.86
OAc
4g
0.94
1.01
3.00
2.17
2.07
6.31
3.17
8.0 7.5 7.0 6.5 6.0 5.5 5.0 4.5 4.0 3.5 3.0 2.5 2.0 1.5 1.0 0.5 0.0 -0.5
f1 (ppm)

Data File Name	C:/ Users/ zhanglab1/ Desktop/ NMR/ jikg/ CASE-SM/ jkg-II_234-SM-P-C.fid/ fid
Title	jkg-II_234-SM-P-C
Solvent	CDCl3
Acquisition Date	2012-03-02T16:29:42
Spectrometer Frequency	125.70

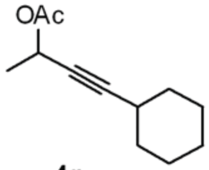

4g

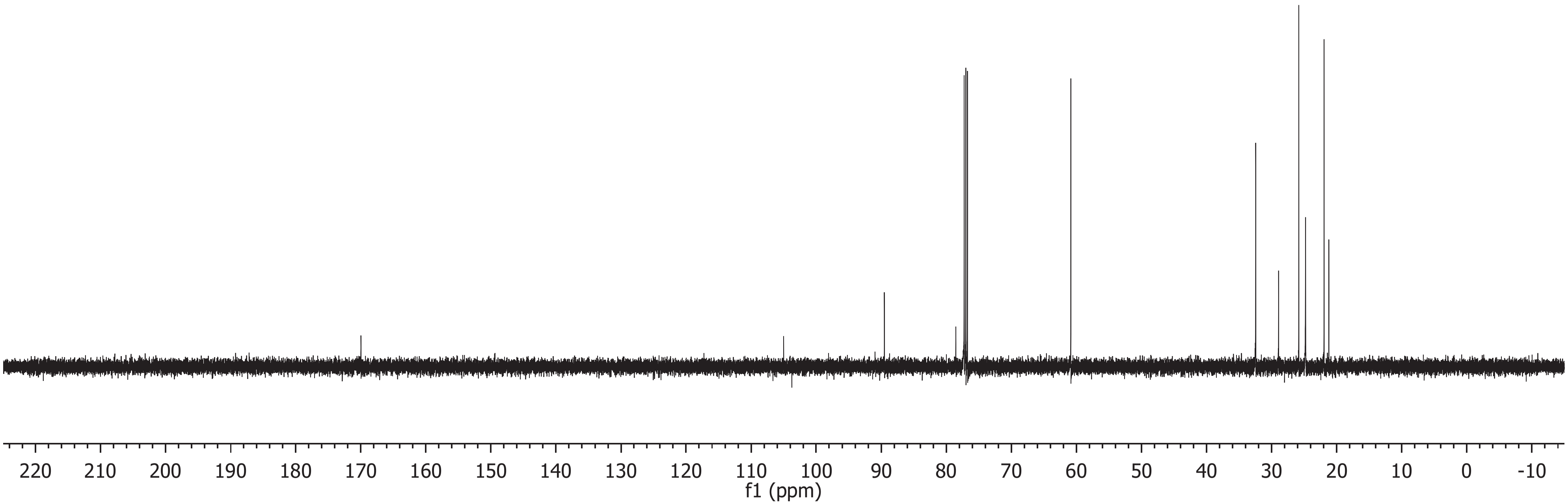

Data File Name	C:/ Users/ zhanglab1/ Desktop/ NMR/ jikg/ CASE-SM/ jkg-III-58-H.fid/ fid
Title	jkg-III-58-H
Solvent	CDCl3
Acquisition Date	2012-05-04T22:38:21
Spectrometer Frequency	499.86

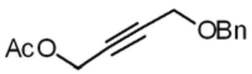

4h

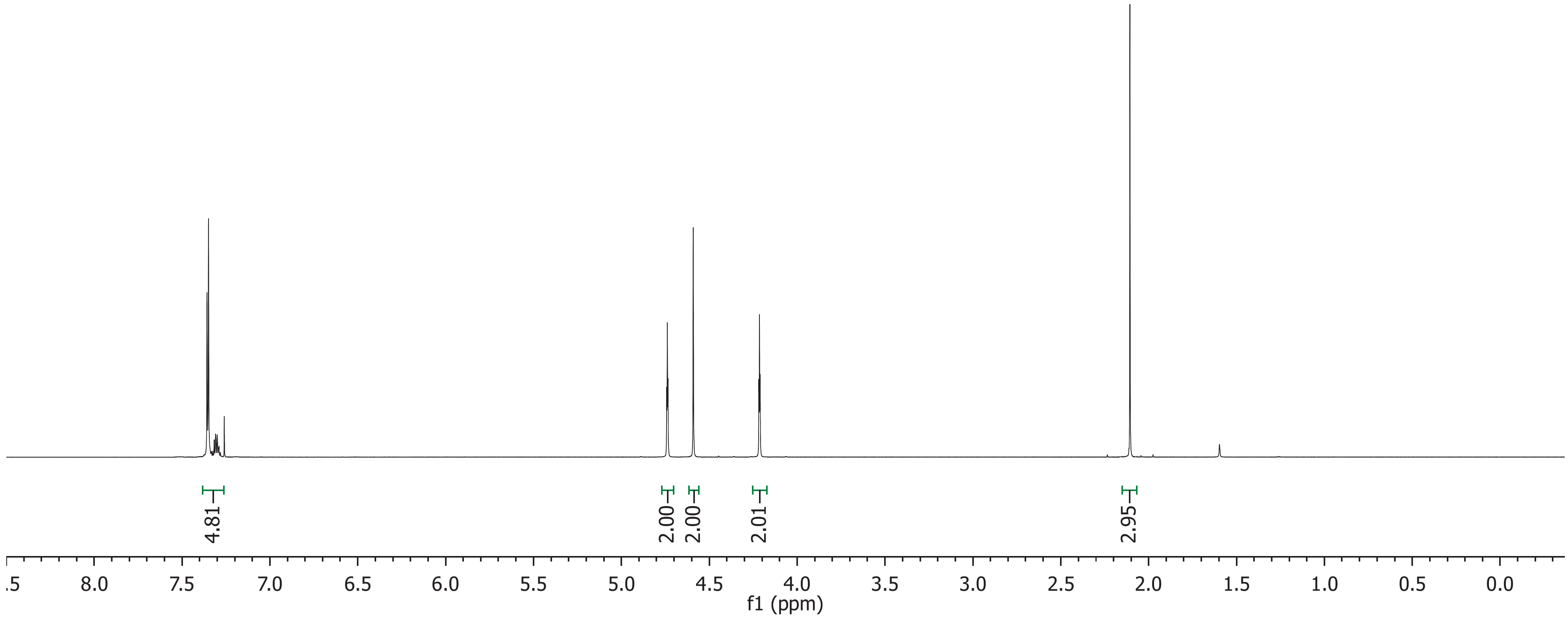

Data File Name	C:/ Users/ zhanglab1/ Desktop/ NMR/ jikg/ CASE-SM/ jkg-III-58-C.fid/ fid
Title	jkg-III-58-C
Solvent	CDCl3
Acquisition Date	2012-05-04T22:40:17
Spectrometer Frequency	125.70

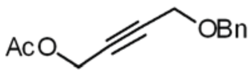

4h

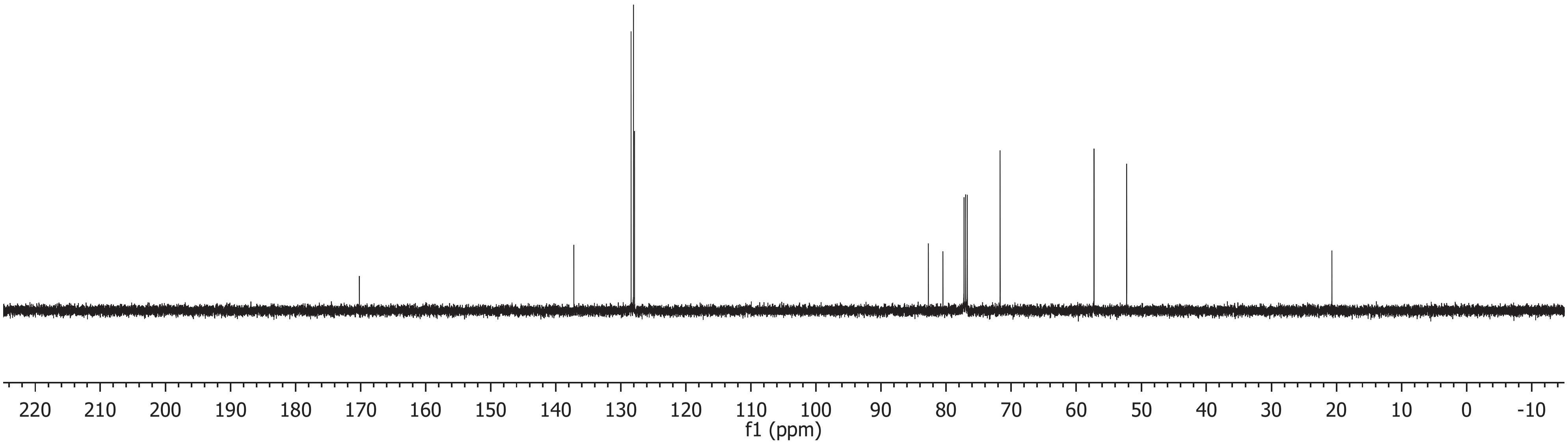

Data File Name	C:/ Users/ zhanglab1/ Desktop/ NMR/ jikg/ CASE-SM/ jkg-II-251-SM-H.fid/ fid
Title	jkg-II-251-SM-H
Solvent	CDCl3
Acquisition Date	2012-03-08T21:41:42
Spectrometer Frequency	499.86

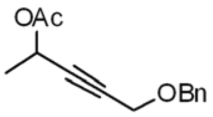

4i

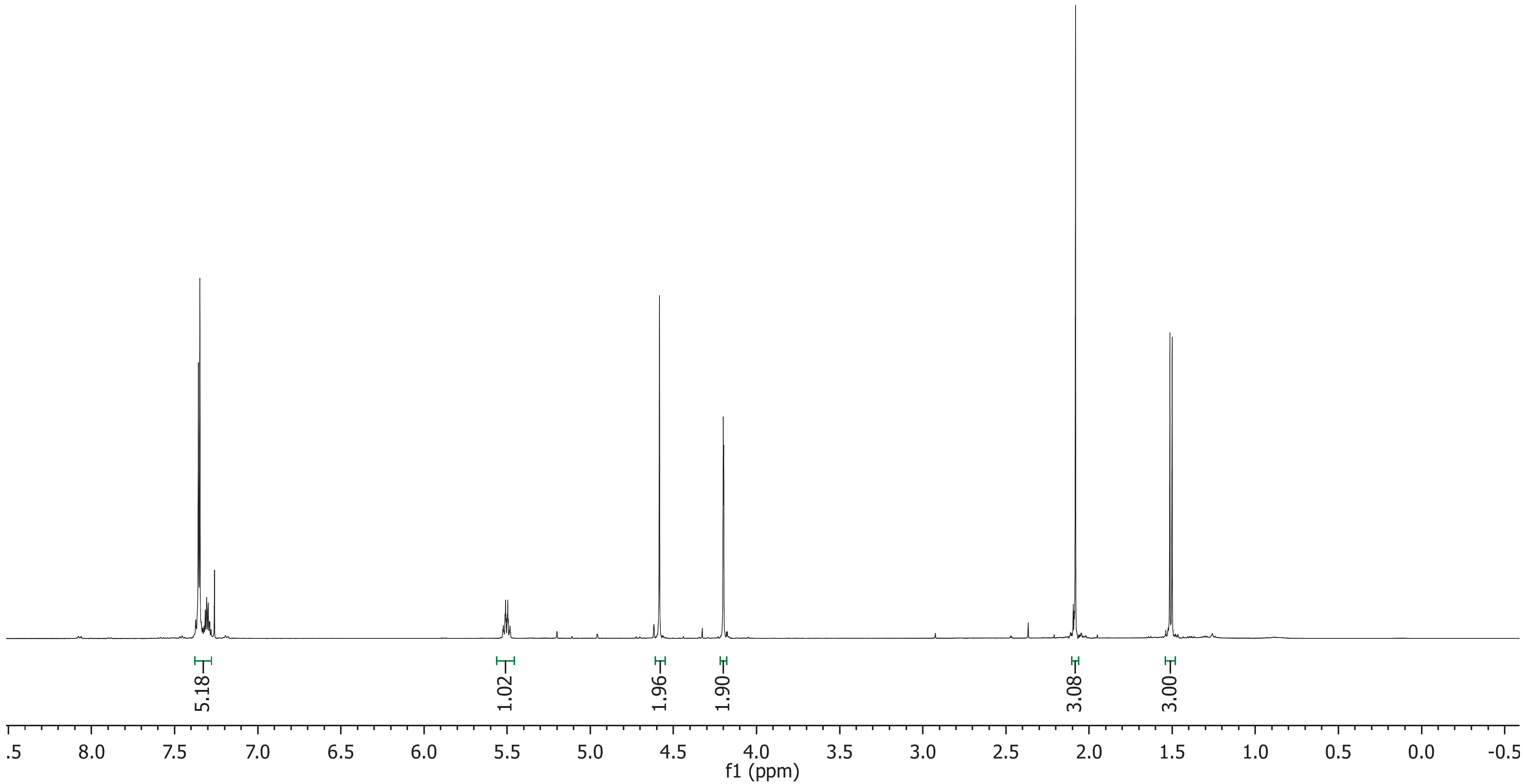

Data File Name	C:/ Users/ zhanglab1/ Desktop/ NMR/ jikg/ CASE-SM/ jkg-II-251-SM-C.fid/ fid
Title	jkg-II-251-SM-C
Solvent	CDCl3
Acquisition Date	2012-03-08T21:44:36
Spectrometer Frequency	125.70

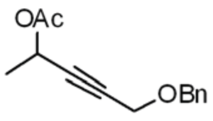

4i

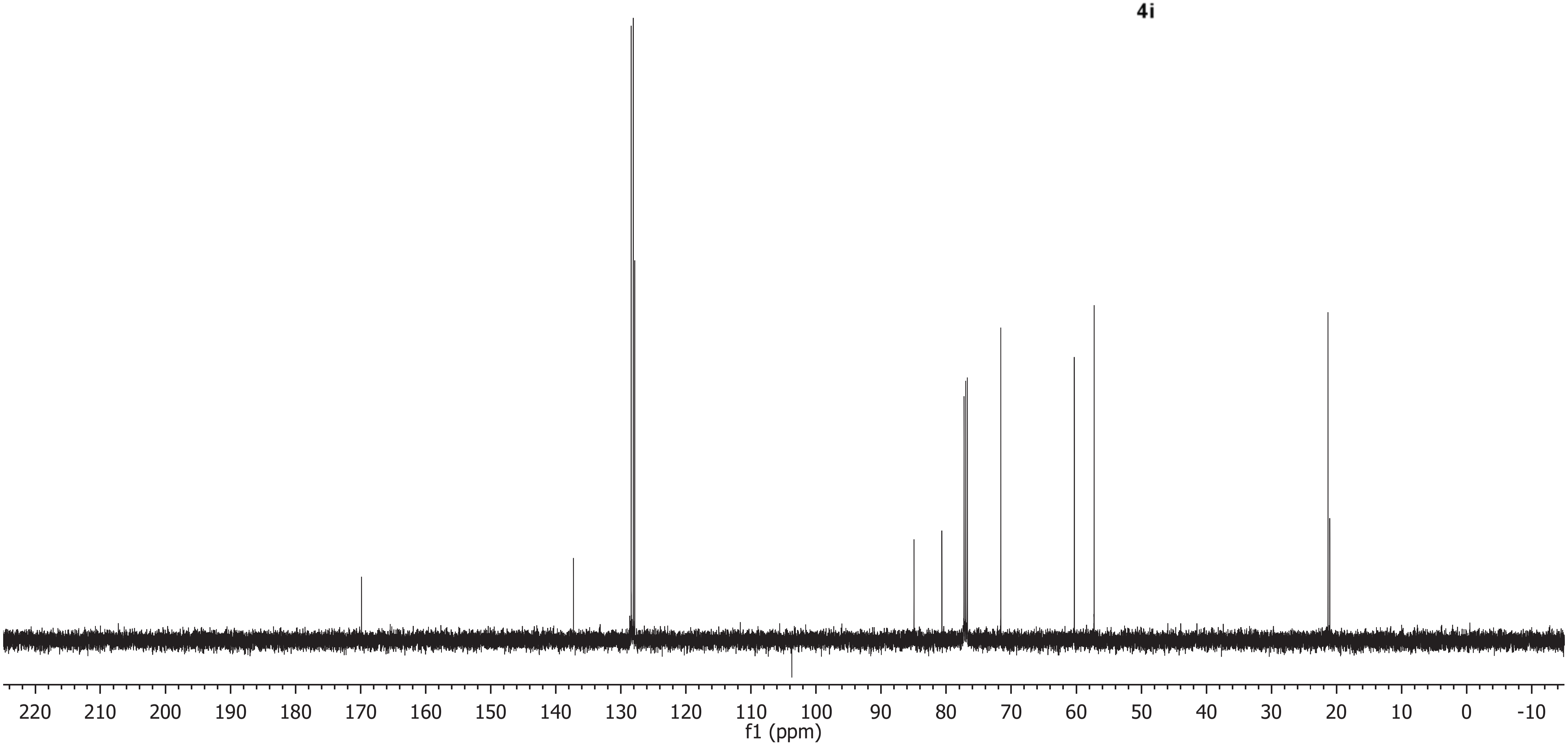

Data File Name	C:/ Users/ zhanglab1/ Desktop/ NMR/ jikg/ CASE-SM/ jkg-II-247B-SM-H.fid/ fid
Title	jkg-II-247B-SM-H
Solvent	CDCl3
Acquisition Date	2012-03-07T14:32:25
Spectrometer Frequency	499.86

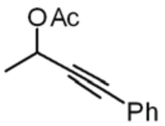

4j

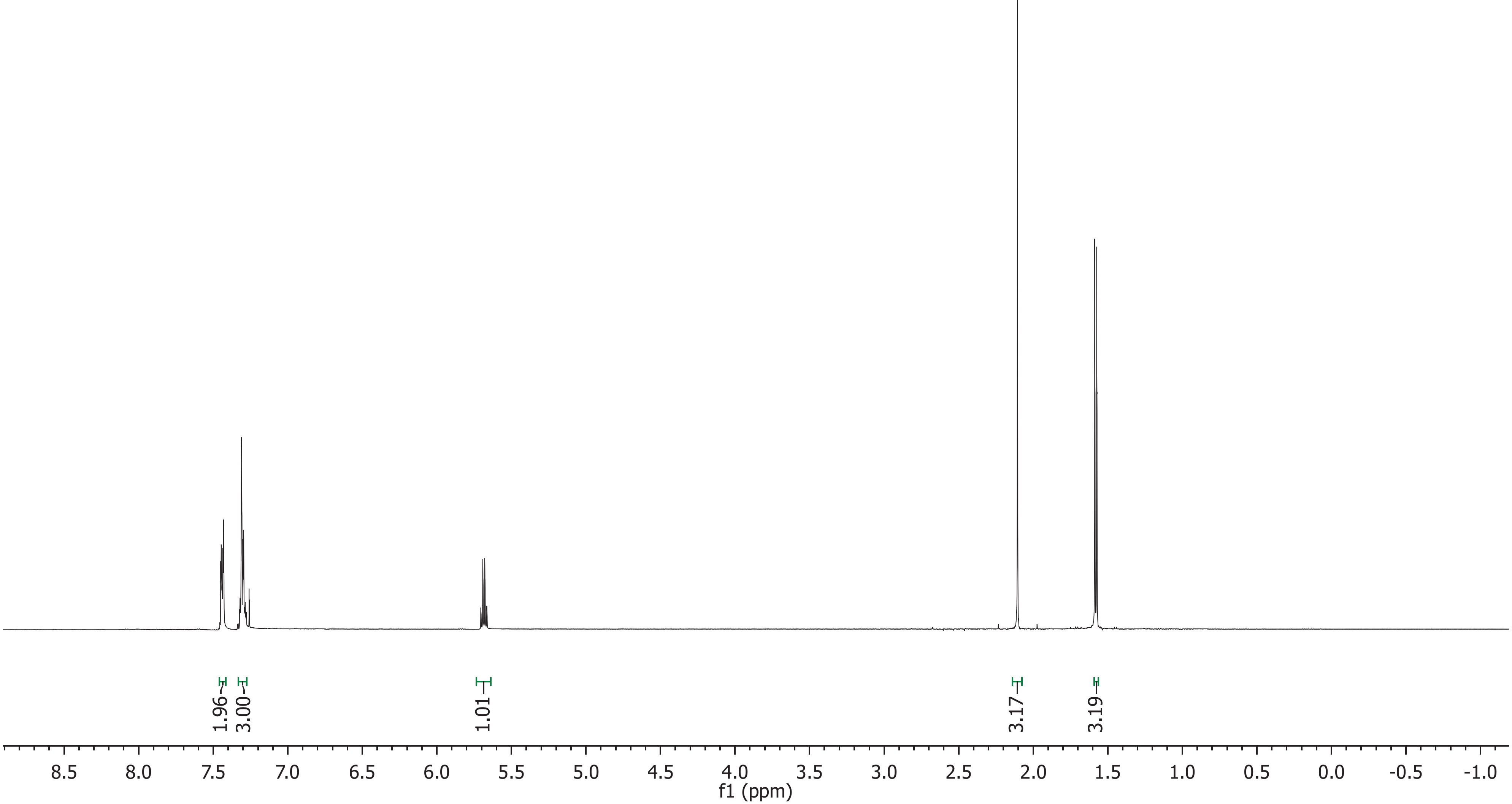

Data File Name	C:/ Users/ zhanglab1/ Desktop/ NMR/ jikg/ CASE-SM/ jkg-II-247B-SM-C.fid/ fid
Title	jkg-II-247B-SM-C
Solvent	CDCl3
Acquisition Date	2012-03-07T14:34:04
Spectrometer Frequency	125.70

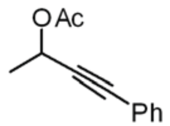

4j

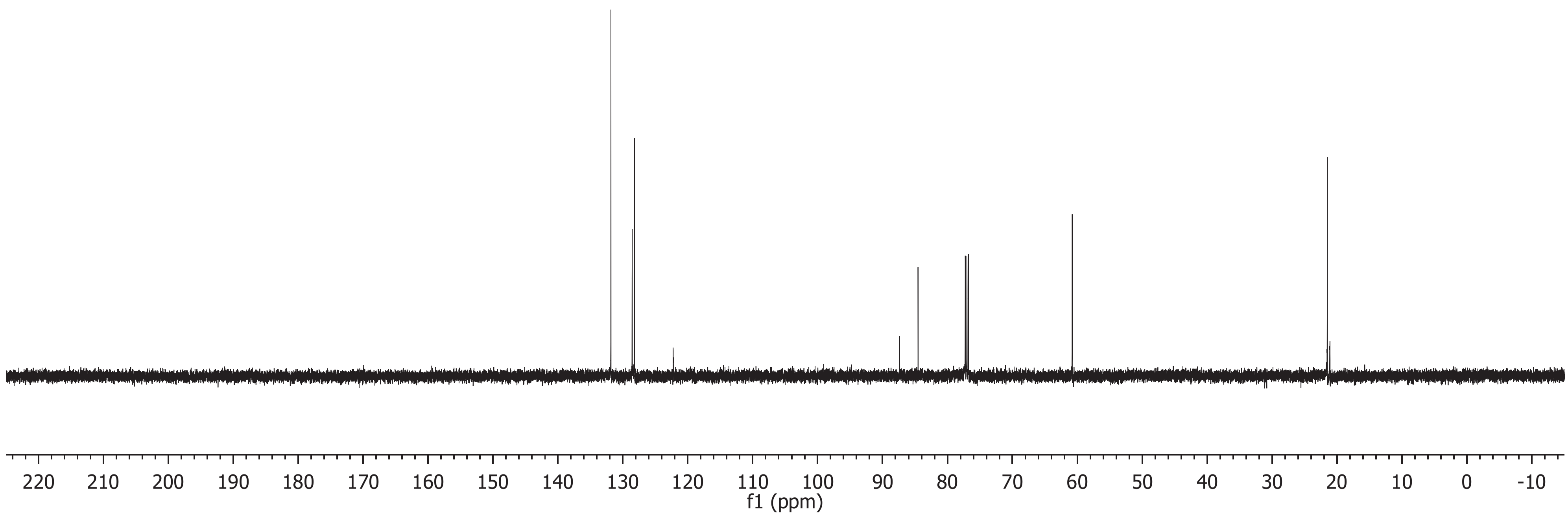

Data File Name	C:/ Users/ zhanglab1/ Desktop/ NMR/ jikg/ CASE-SM/ jkg-I-272-P-H.fid/ fid
Title	jkg-I-272-P-H
Solvent	cdcl3
Acquisition Date	2012-02-29T17:35:18
Spectrometer Frequency	599.63

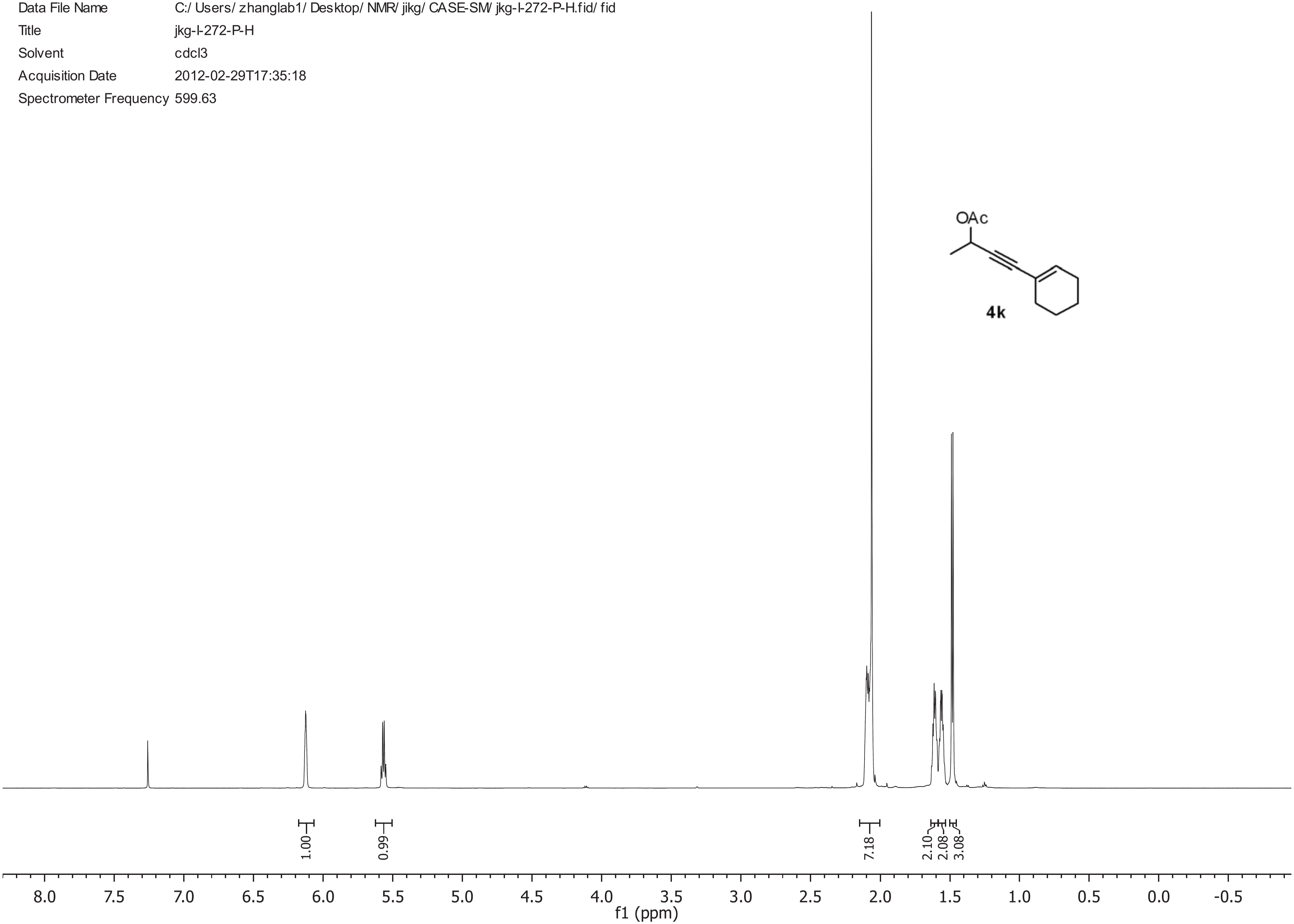

Data File Name	C:/ Users/ zhanglab1/ Desktop/ NMR/ jikg/ CASE-SM/ jkg-I-272-P-C.fid/ fid
Title	jkg-I-272-P-C
Solvent	cdcl3
Acquisition Date	2012-02-29T17:36:51
Spectrometer Frequency	150.79

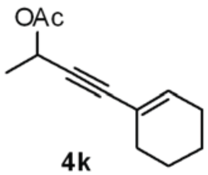

4k

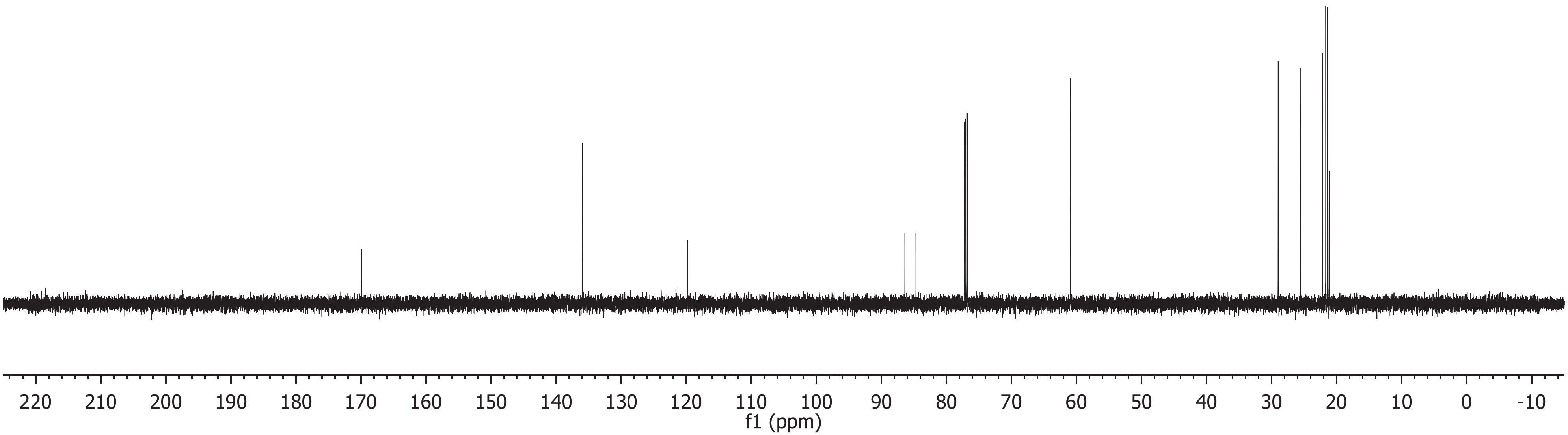

Data File Name	C:/ Users/ zhanglab1/ Desktop/ NMR/ jikg/ CASE-SM/ jkg-II-243-B-3-P-H.fid/ fid
Title	jkg-II-243-B-3-P-H
Solvent	cdcl3
Acquisition Date	2012-03-05T22:28:09
Spectrometer Frequency	599.63

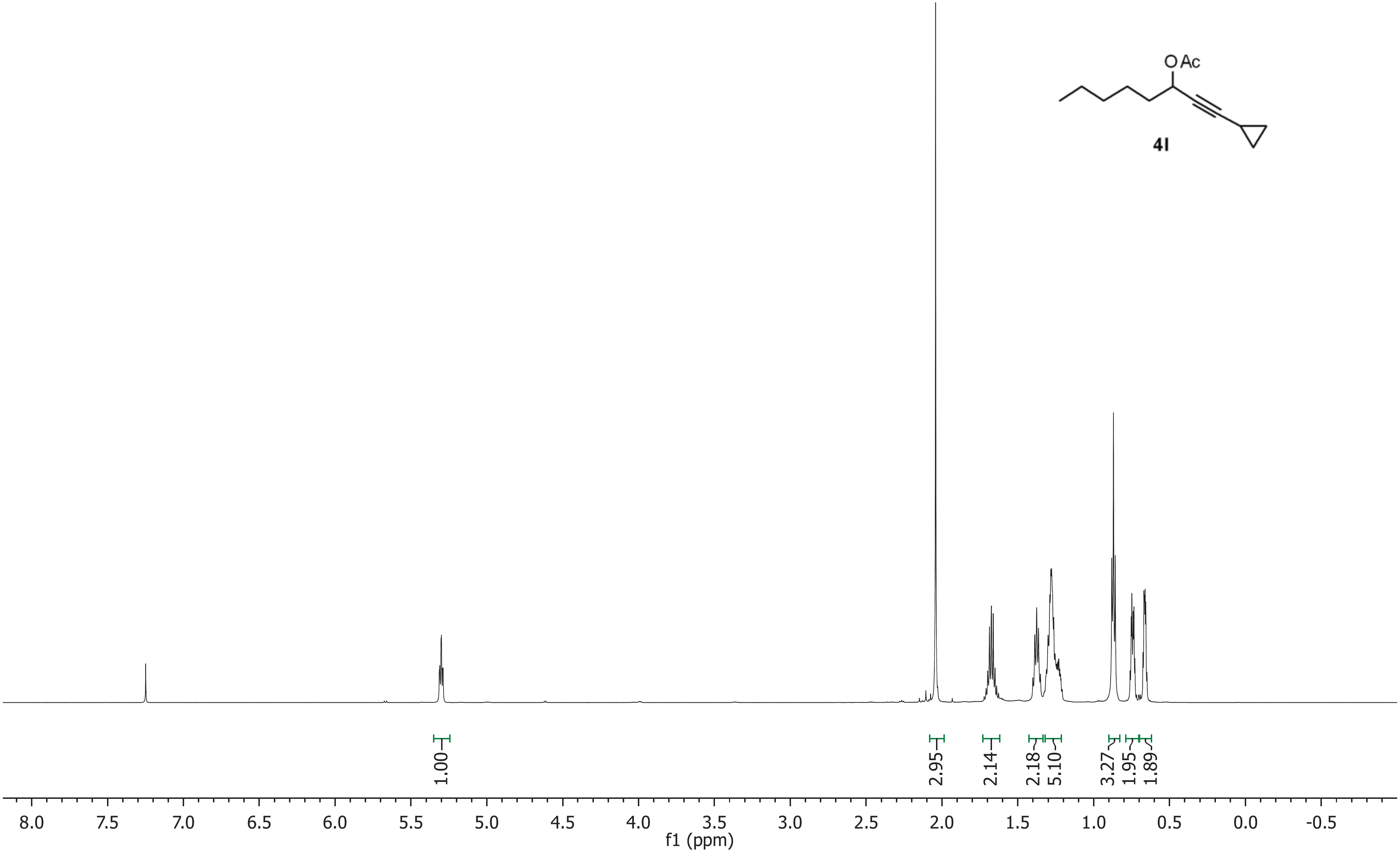

4l

Data File Name	C:/ Users/ zhanglab1/ Desktop/ NMR/ jikg/ CASE-SM/ jkg-II-243-B-3-P-C.fid/ fid
Title	jkg-II-243-B-3-P-C
Solvent	cdcl3
Acquisition Date	2012-03-05T22:30:57
Spectrometer Frequency	150.79

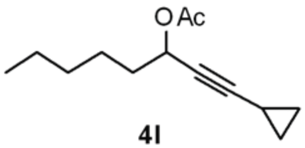

4l

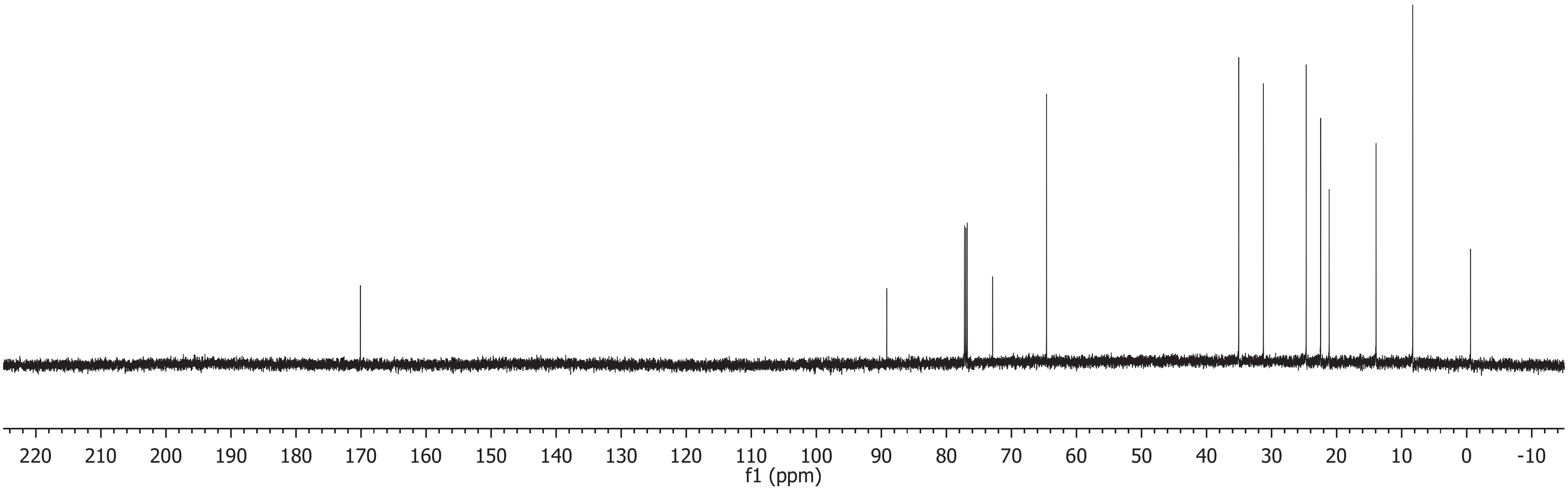

Data File Name	C:/ Users/ zhanglab1/ Desktop/ NMR/ jikg/ CASE-SM/ jkg-II-240B-SM-P-H.fid/ fid
Title	jkg-II-240B-SM-P-H
Solvent	cdcl3
Acquisition Date	2012-03-04T21:25:39
Spectrometer Frequency	599.63

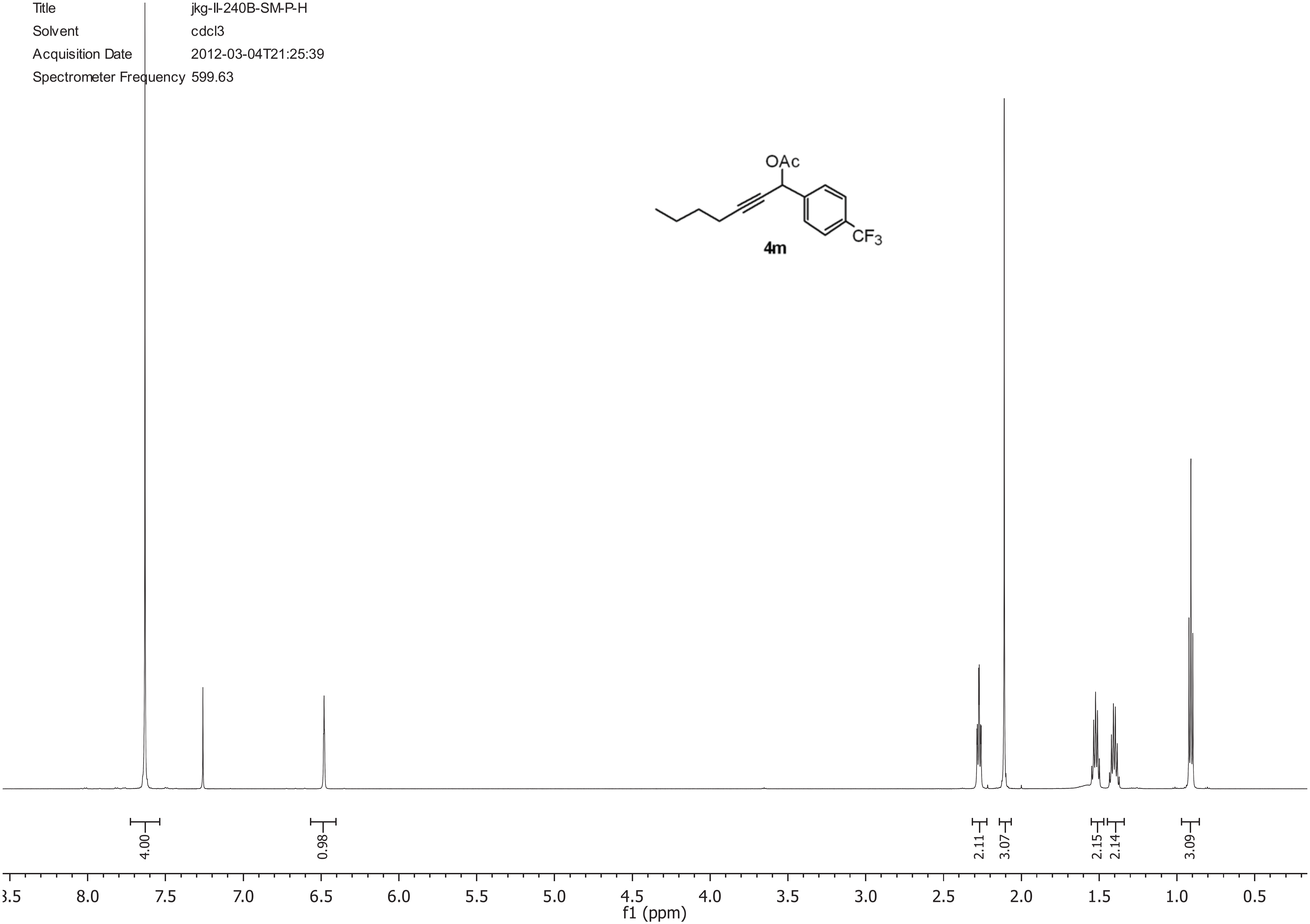

Data File Name	C:/ Users/ zhanglab1/ Desktop/ NMR/ jikg/ CASE-SM/ jkg-II-240B-SM-P-C.fid/ fid
Title	jkg-II-240B-SM-P-C
Solvent	cdcl3
Acquisition Date	2012-03-04T21:28:11
Spectrometer Frequency	150.79

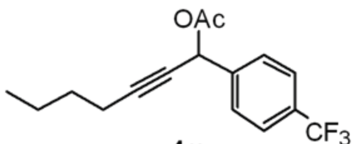

4m

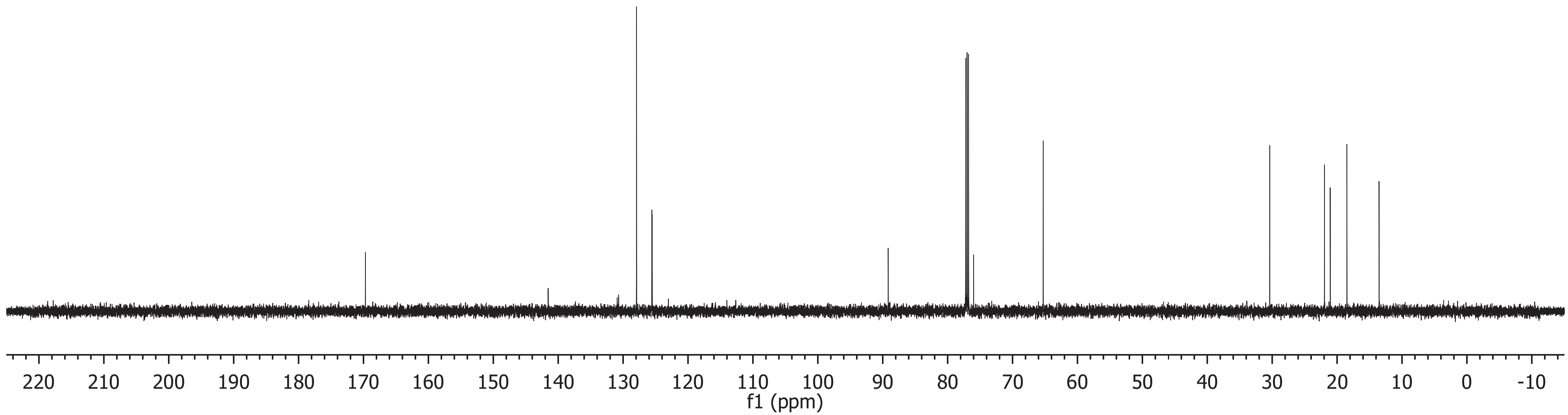

Data File Name	C:/ Users/ zhanglab1/ Desktop/ NMR/ jikg/ product/ jkg-II-246A-p-H.fid/ fid
Title	jkg-II-246A-p-H
Solvent	CDCl3
Acquisition Date	2012-03-07T14:26:59
Spectrometer Frequency	499.86

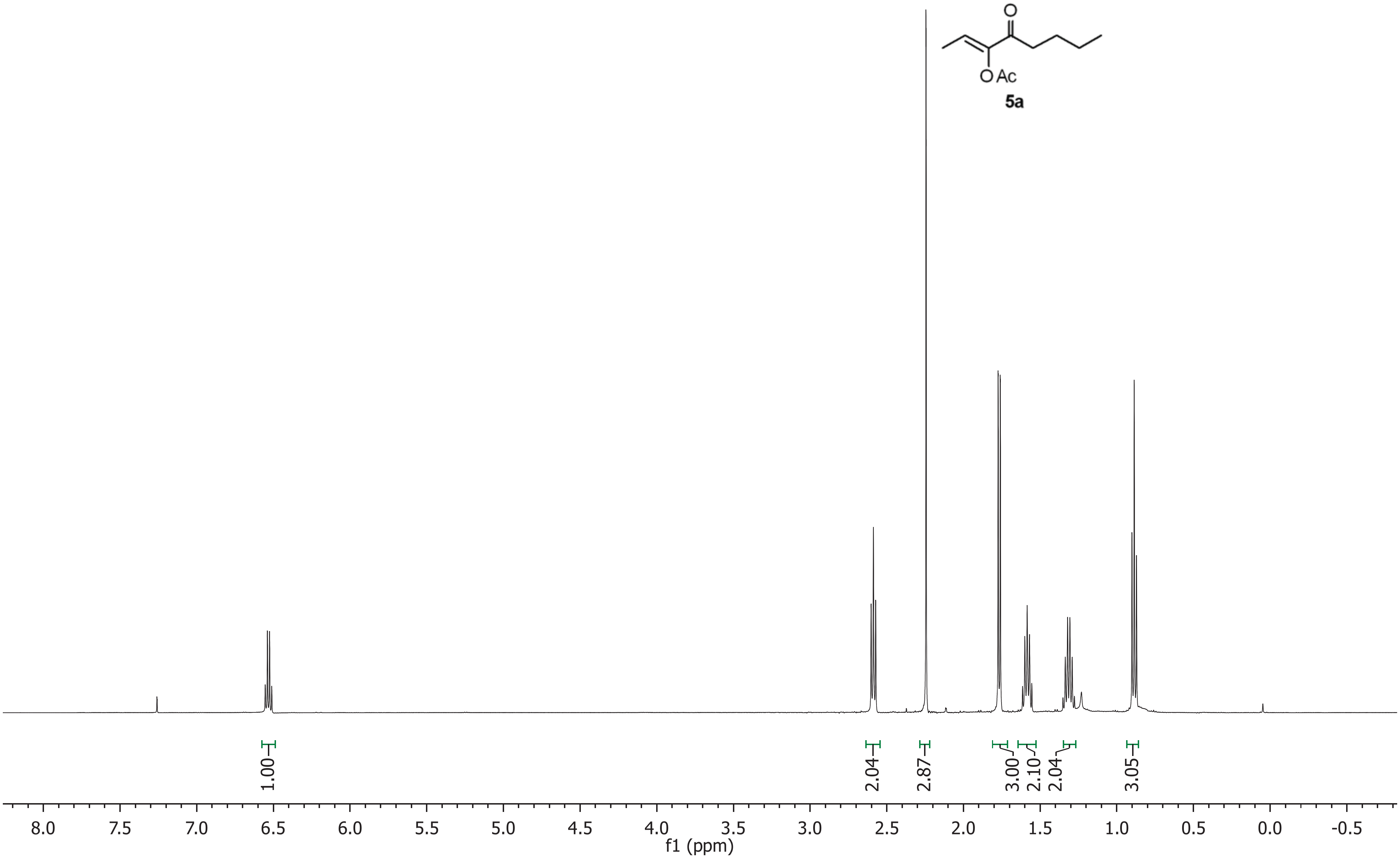

Data File Name	C:/ Users/ zhanglab1/ Desktop/ NMR/ jikg/ product/ jkg-II-246A-p-C.fid/ fid
Title	jkg-II-246A-p-C
Solvent	CDCl3
Acquisition Date	2012-03-07T14:28:26
Spectrometer Frequency	125.70

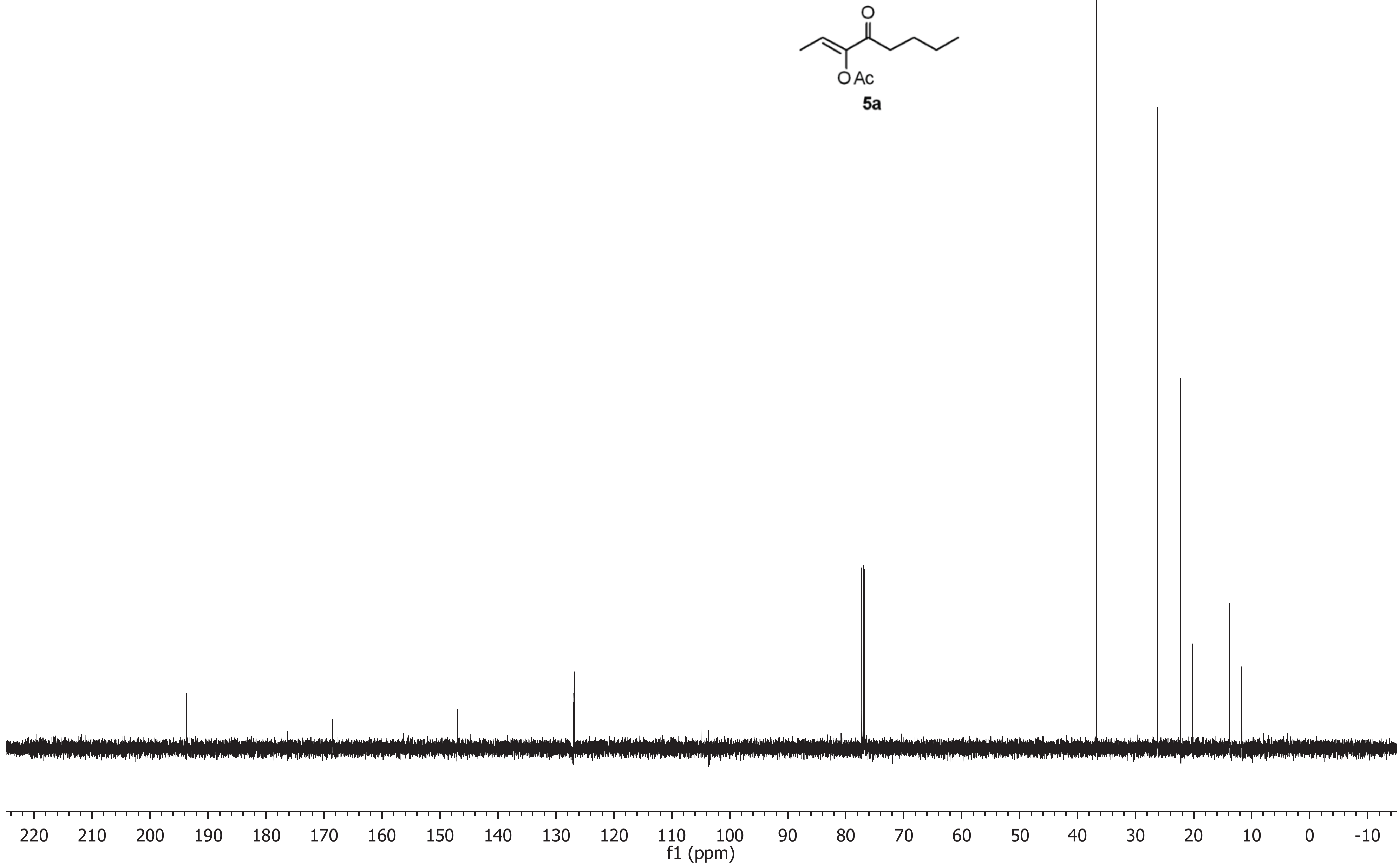

Data File Name	C:/ Users/ zhanglab1/ Desktop/ NMR/ jikg/ product/ jkg-II-228-P-H.fid/ fid
Title	jkg-II-228-P-H
Solvent	CDCl3
Acquisition Date	2012-02-27T22:26:10
Spectrometer Frequency	499.86

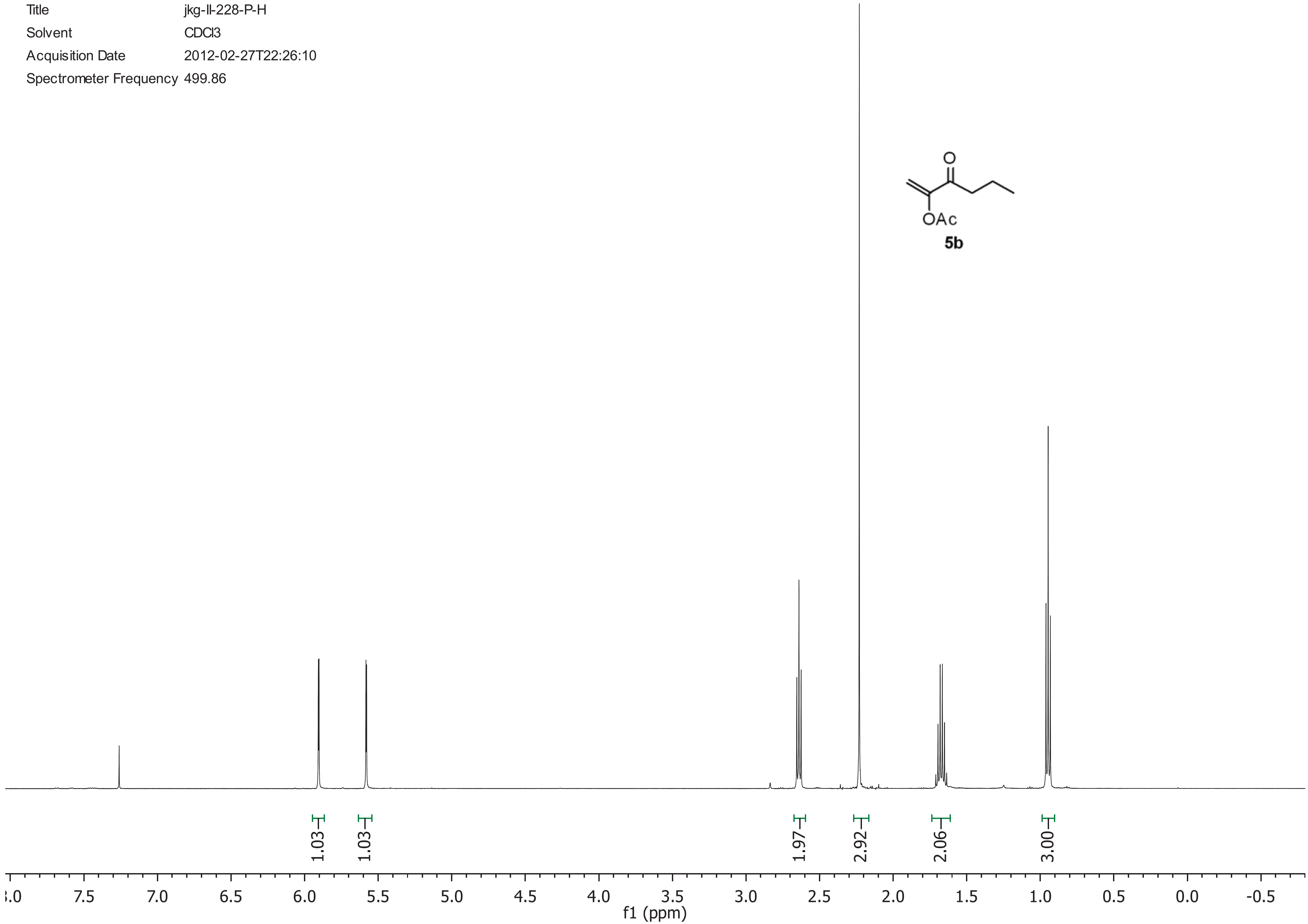

Data File Name	C:/ Users/ zhanglab1/ Desktop/ NMR/ jikg/ product/ jkg-II-228-P-C.fid/ fid
Title	jkg-II-228-P-C
Solvent	CDCl3
Acquisition Date	2012-02-27T22:27:39
Spectrometer Frequency	125.70

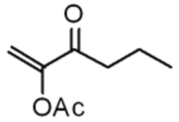

5b

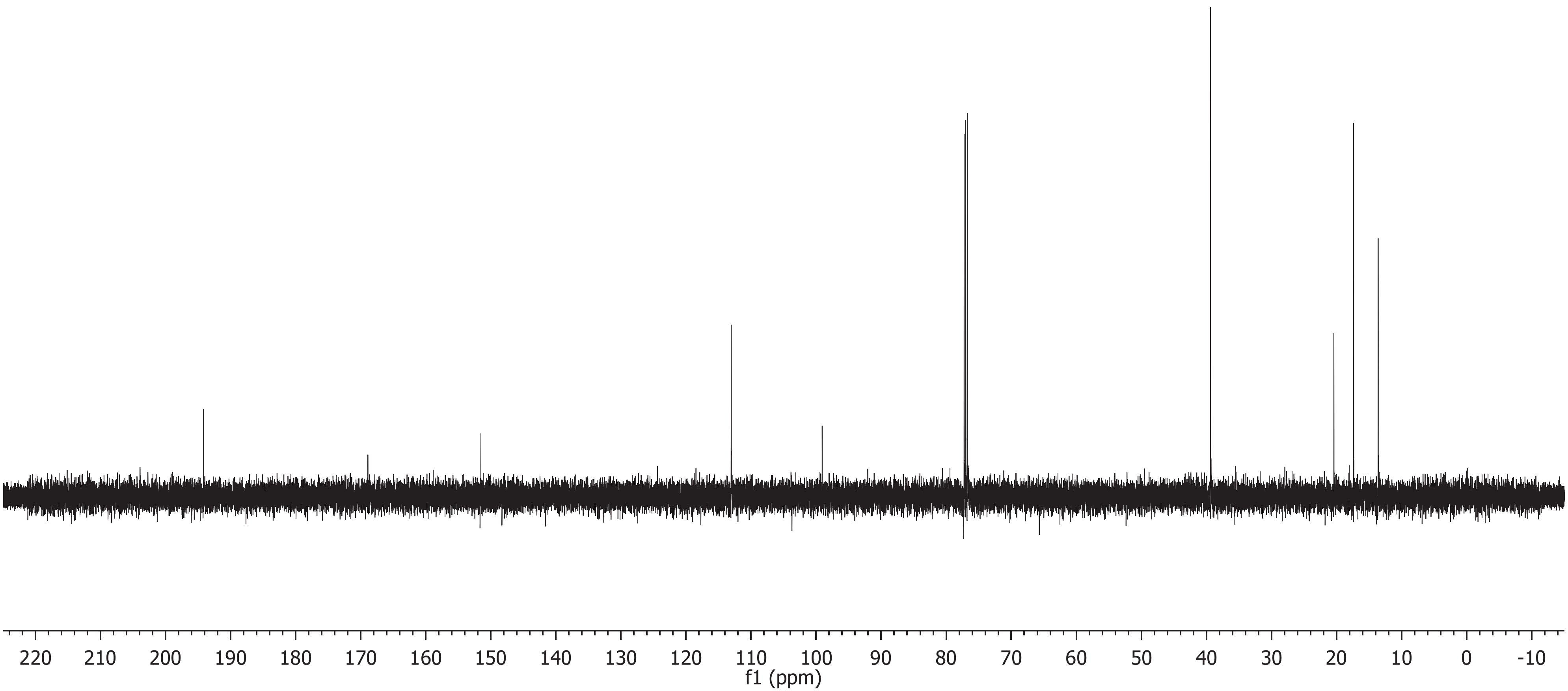

Data File Name	C:/ Users/ zhanglab1/ Desktop/ NMR/ jikg/ product/ jkg-III-71-1-H.fid/ fid
Title	jkg-III-71-1-H
Solvent	CDCl3
Acquisition Date	2012-05-15T21:02:27
Spectrometer Frequency	499.86

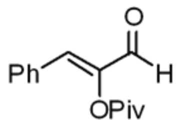

5c

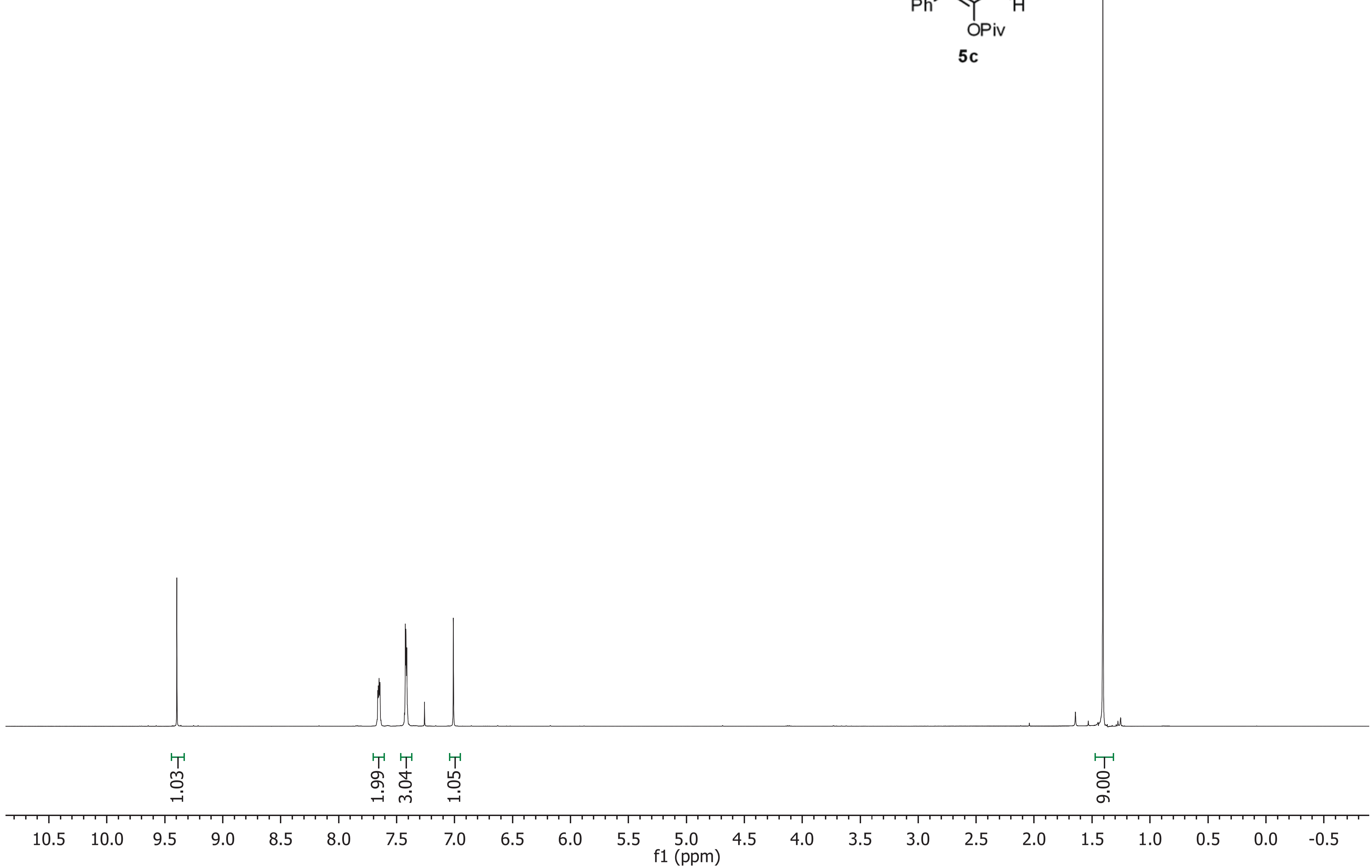

Data File Name	C:/ Users/ zhanglab1/ Desktop/ NMR/ jikg/ product/ jkg-III-71-1-C.fid/ fid
Title	jkg-III-71-1-C
Solvent	CDCl3
Acquisition Date	2012-05-15T21:04:55
Spectrometer Frequency	125.70

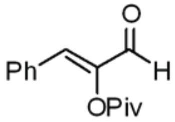

5c

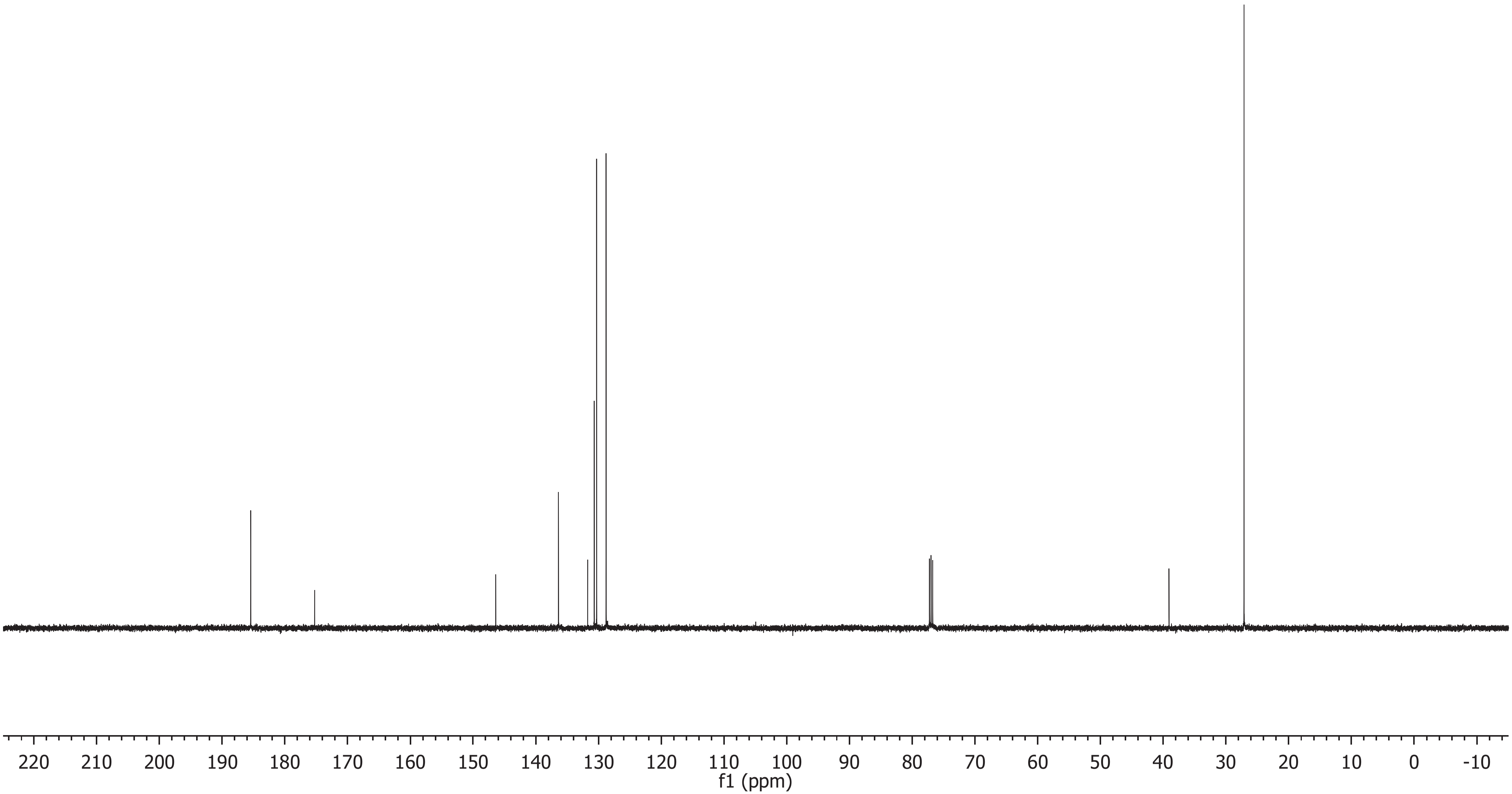

Data File Name	C:/ Users/ zhanglab1/ Desktop/ NMR/ jikg/ product/ jkg-II-234A-P-H.fid/ fid
Title	jkg-II-234A-P-H
Solvent	CDCl3
Acquisition Date	2012-03-01T13:31:45
Spectrometer Frequency	499.86

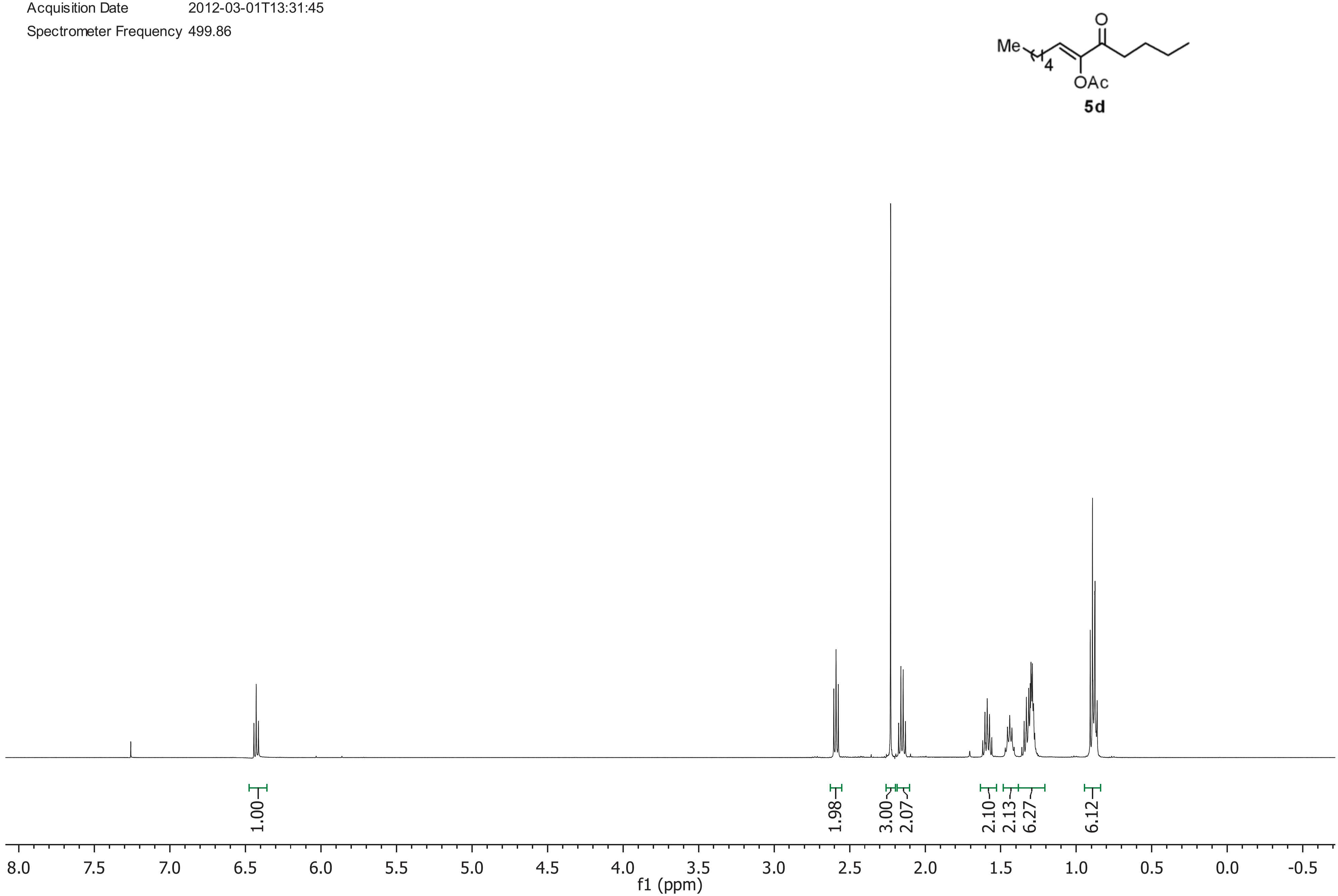

Data File Name	C:/ Users/ zhanglab1/ Desktop/ NMR/ jikg/ product/ jkg-II-234A-P-C.fid/ fid
Title	jkg-II-234A-P-C
Solvent	CDCl3
Acquisition Date	2012-03-01T13:35:01
Spectrometer Frequency	125.70

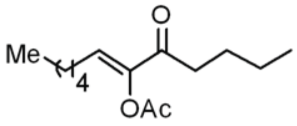

5d

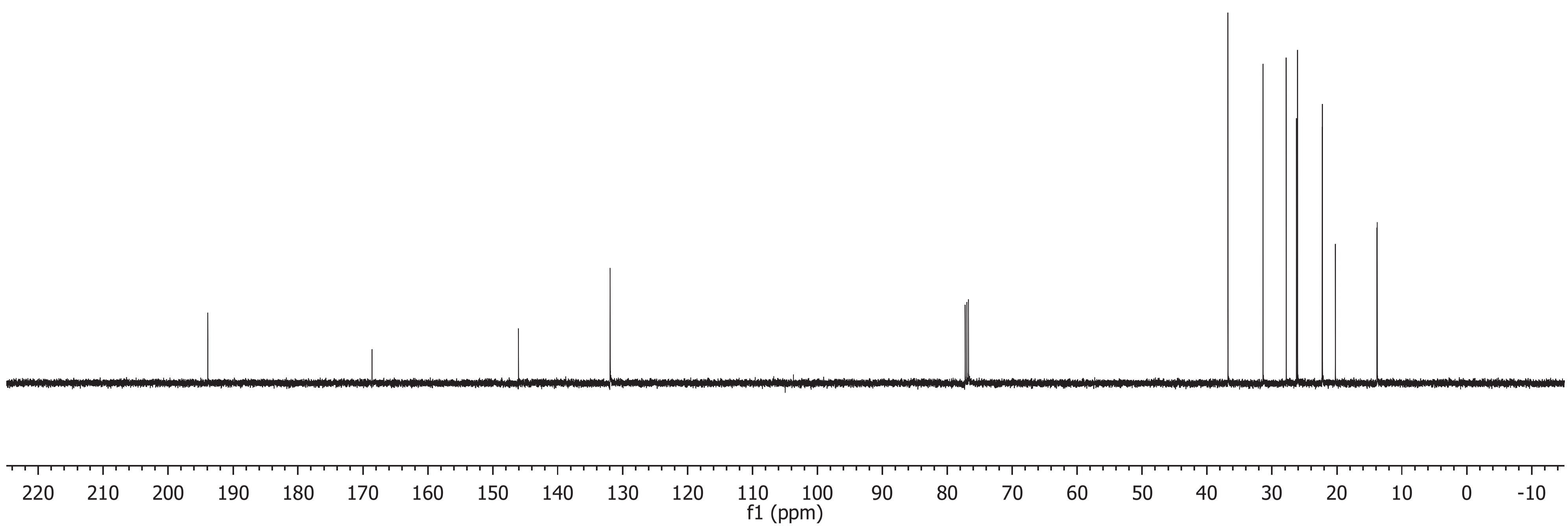

Data File Name	C:/ Users/ zhanglab1/ Desktop/ NMR/ jikg/ product/ jkg-II-243A-P-H.fid/ fid
Title	jkg-II-243A-P-H
Solvent	CDCl3
Acquisition Date	2012-03-06T16:10:11
Spectrometer Frequency	499.86

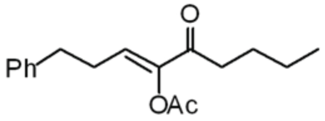

5e

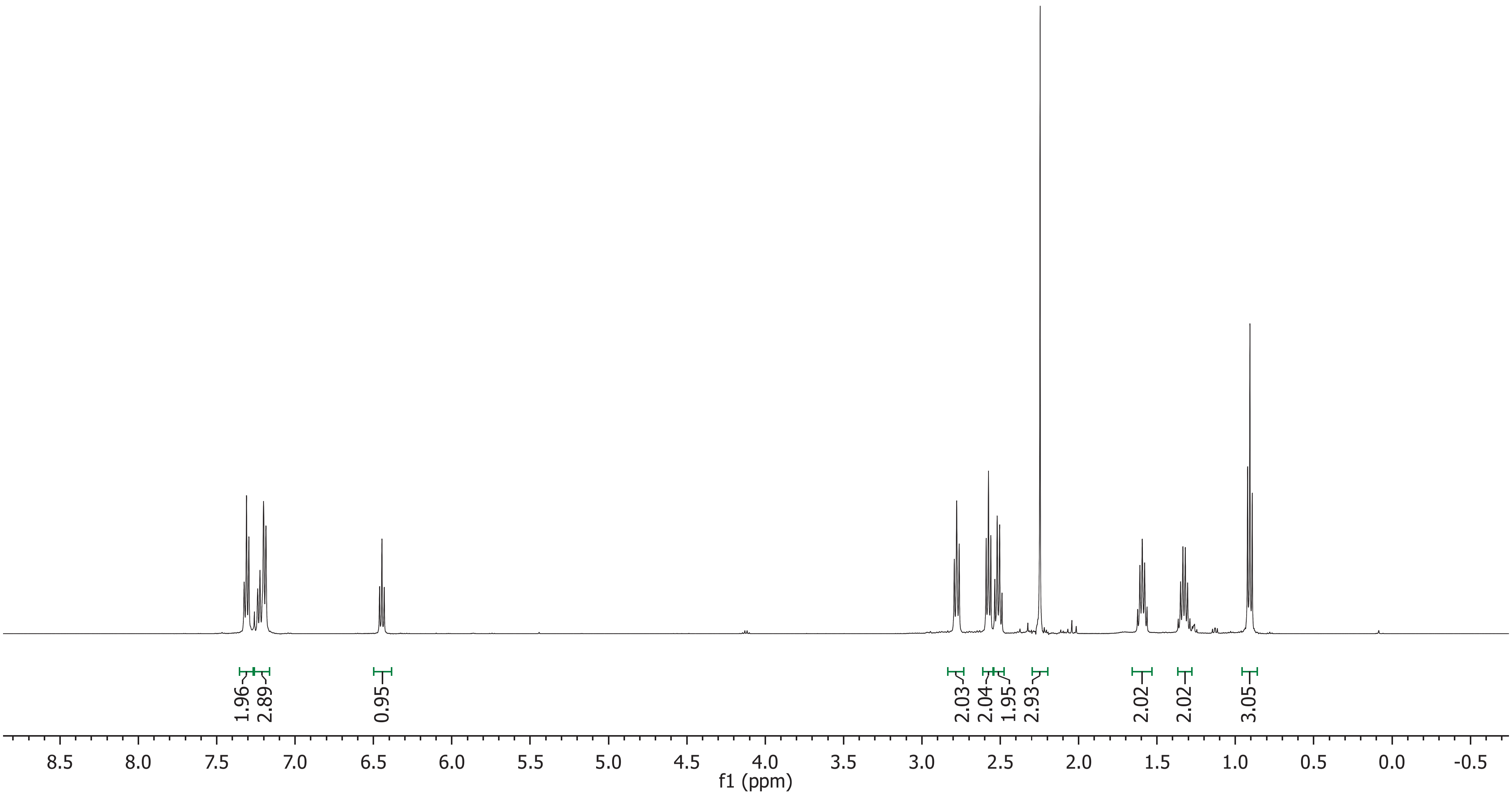

Data File Name	C:/ Users/ zhanglab1/ Desktop/ NMR/ jikg/ product/ jkg-II-243A-P-C.fid/ fid
Title	jkg-II-243A-P-C
Solvent	CDCl3
Acquisition Date	2012-03-06T16:12:37
Spectrometer Frequency	125.70

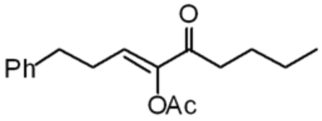

5e

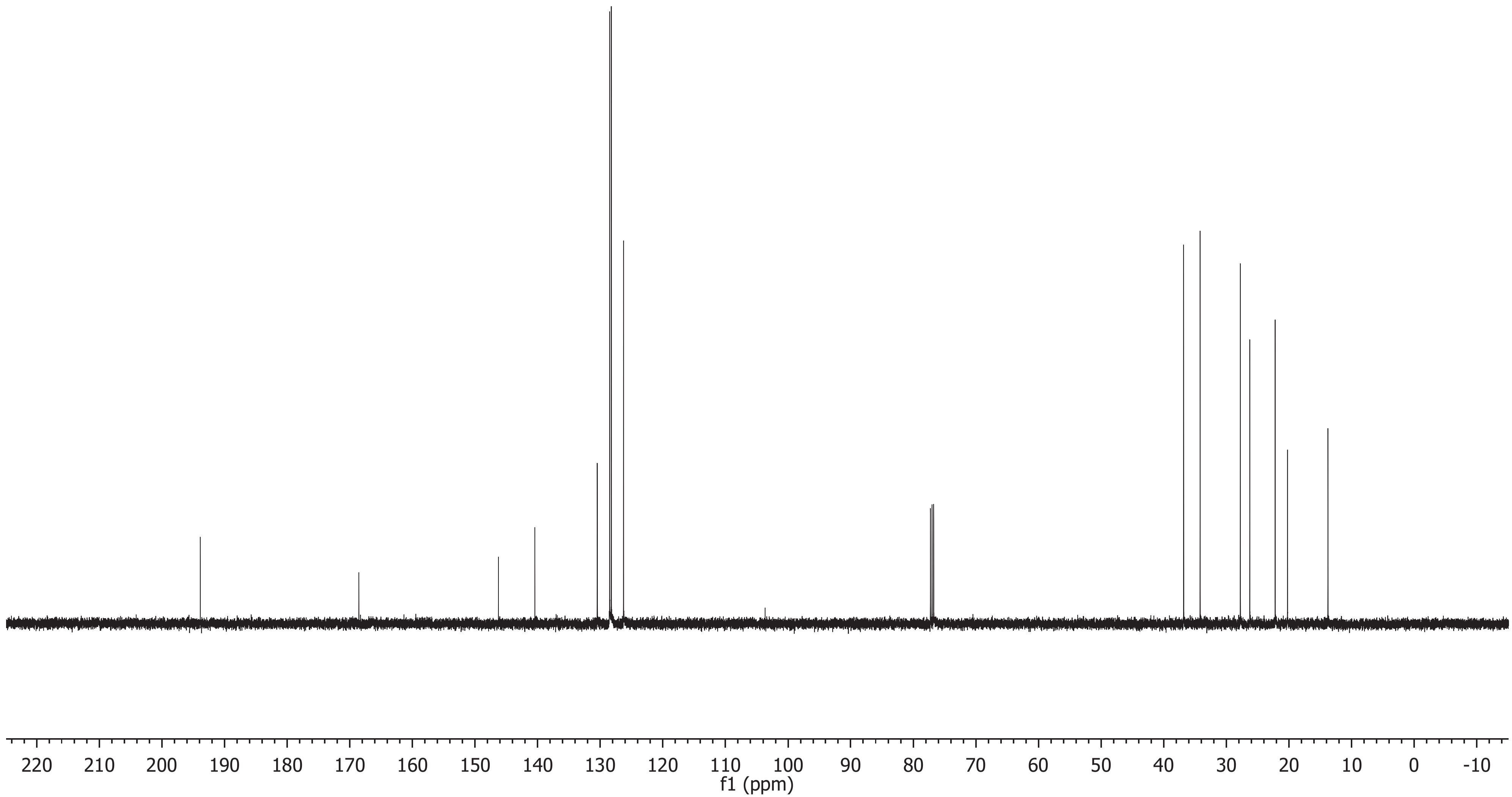

Data File Name	C:/ Users/ zhanglab1/ Desktop/ NMR/ jikg/ product/ jkg-II-244-P-H.fid/ fid
Title	jkg-II-244-P-H
Solvent	CDCl3
Acquisition Date	2012-03-06T16:23:19
Spectrometer Frequency	499.86

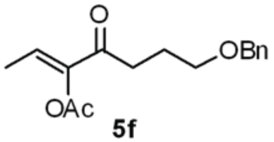

5f

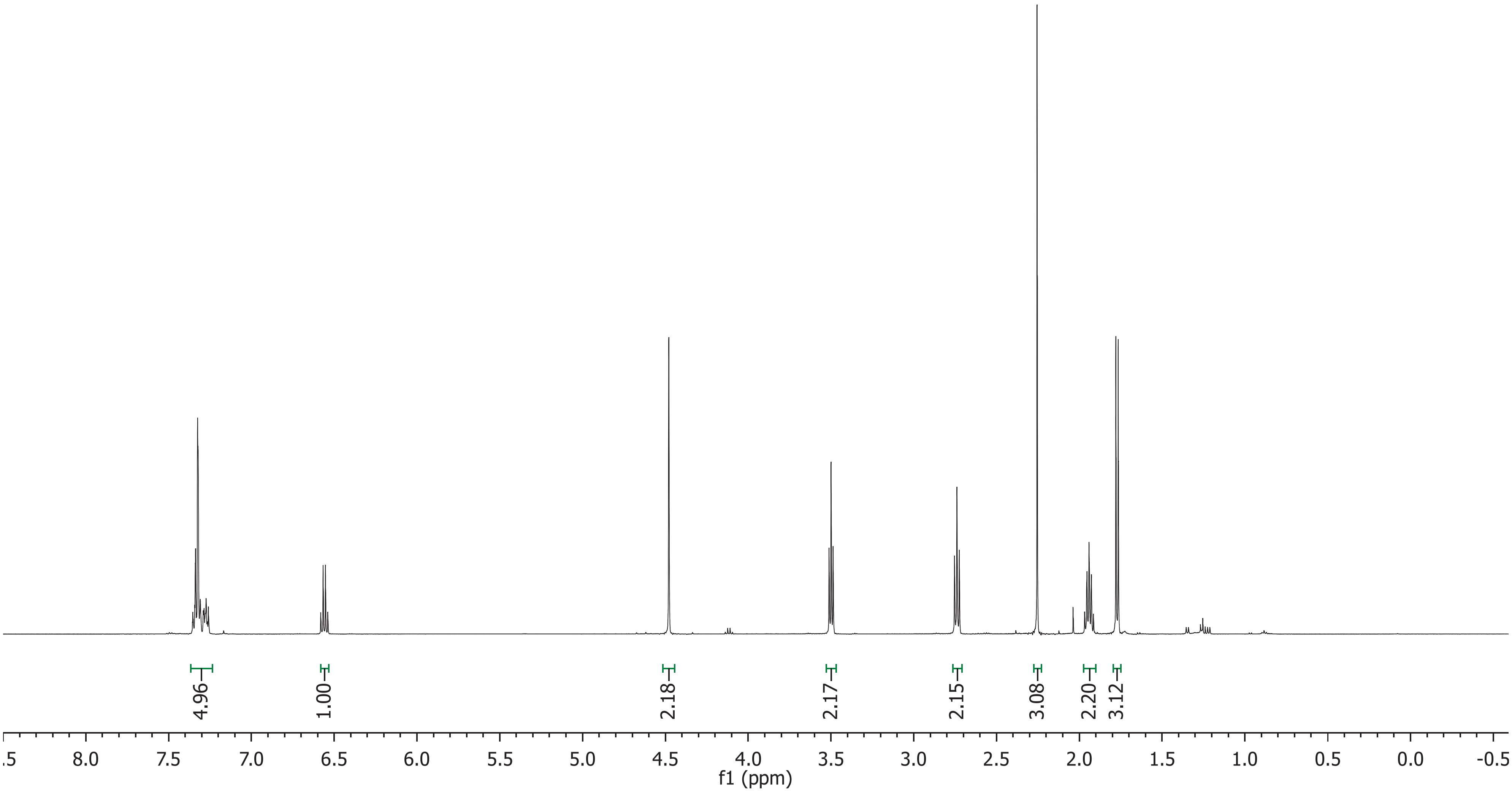

Data File Name	C:/ Users/ zhanglab1/ Desktop/ NMR/ jikg/ product/ jkg-II-244-P-C.fid/ fid
Title	jkg-II-244-P-C
Solvent	CDCl3
Acquisition Date	2012-03-06T16:25:38
Spectrometer Frequency	125.70

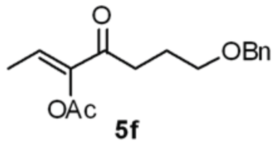

5f

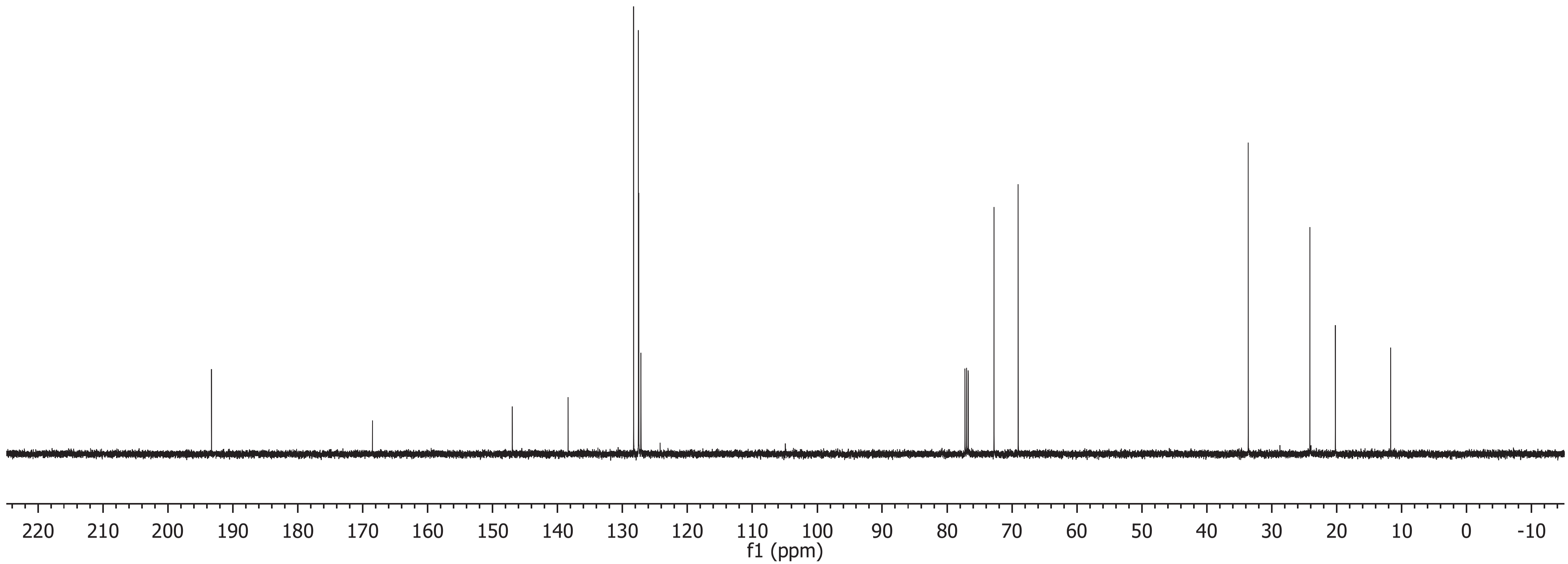

Data File Name	C:/ Users/ zhanglab1/ Desktop/ NMR/ jikg/ product/ jkg-II-234B-P-H.fid/ fid
Title	jkg-II-234B-P-H
Solvent	CDCl3
Acquisition Date	2012-03-02T16:12:42
Spectrometer Frequency	499.86

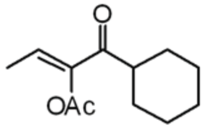

5g

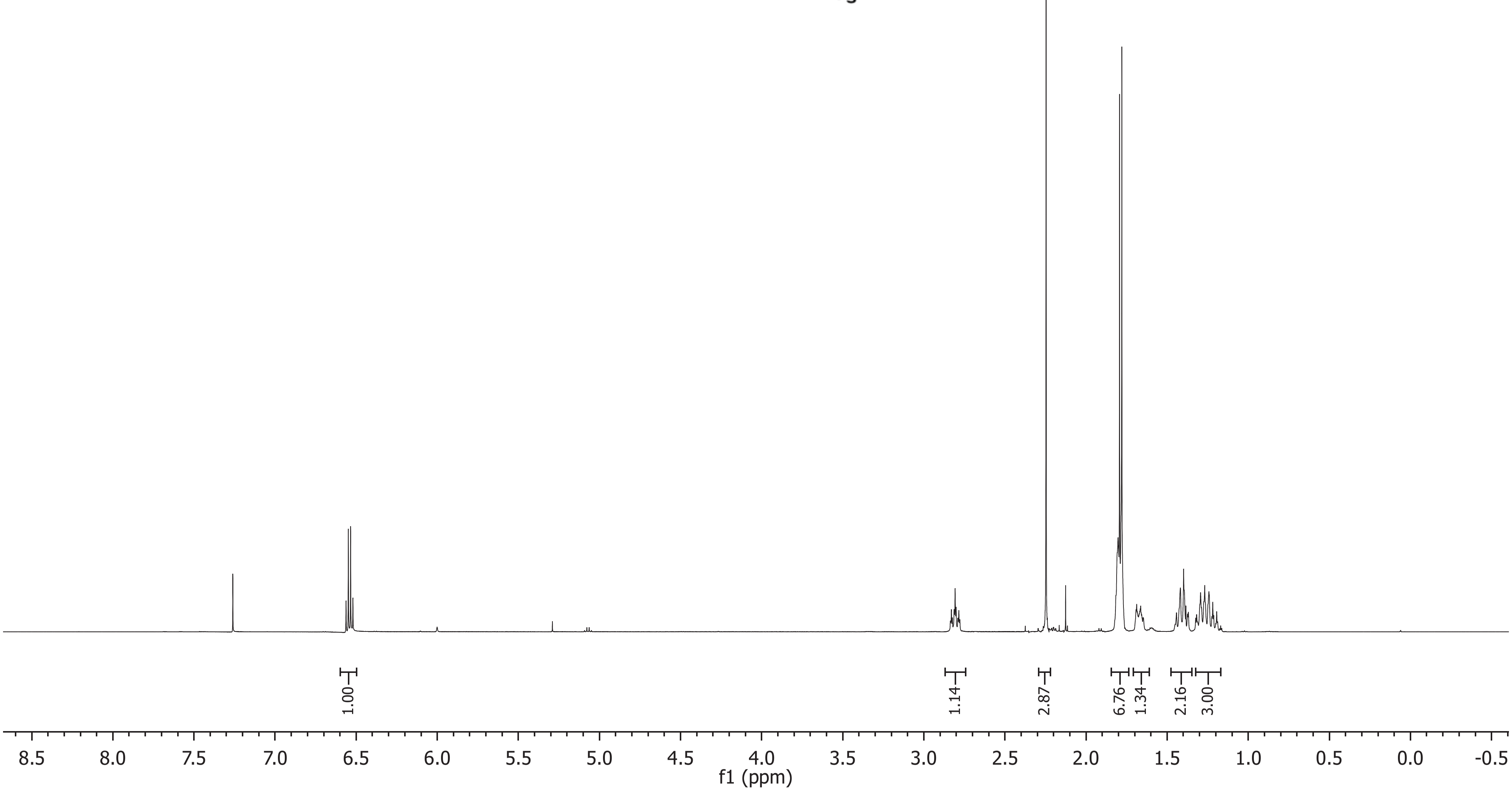

Data File Name	C:/ Users/ zhanglab1/ Desktop/ NMR/ jikg/ product/ jkg-II-234B-P-C.fid/ fid
Title	jkg-II-234B-P-C
Solvent	CDCl3
Acquisition Date	2012-03-02T16:15:56
Spectrometer Frequency	125.70

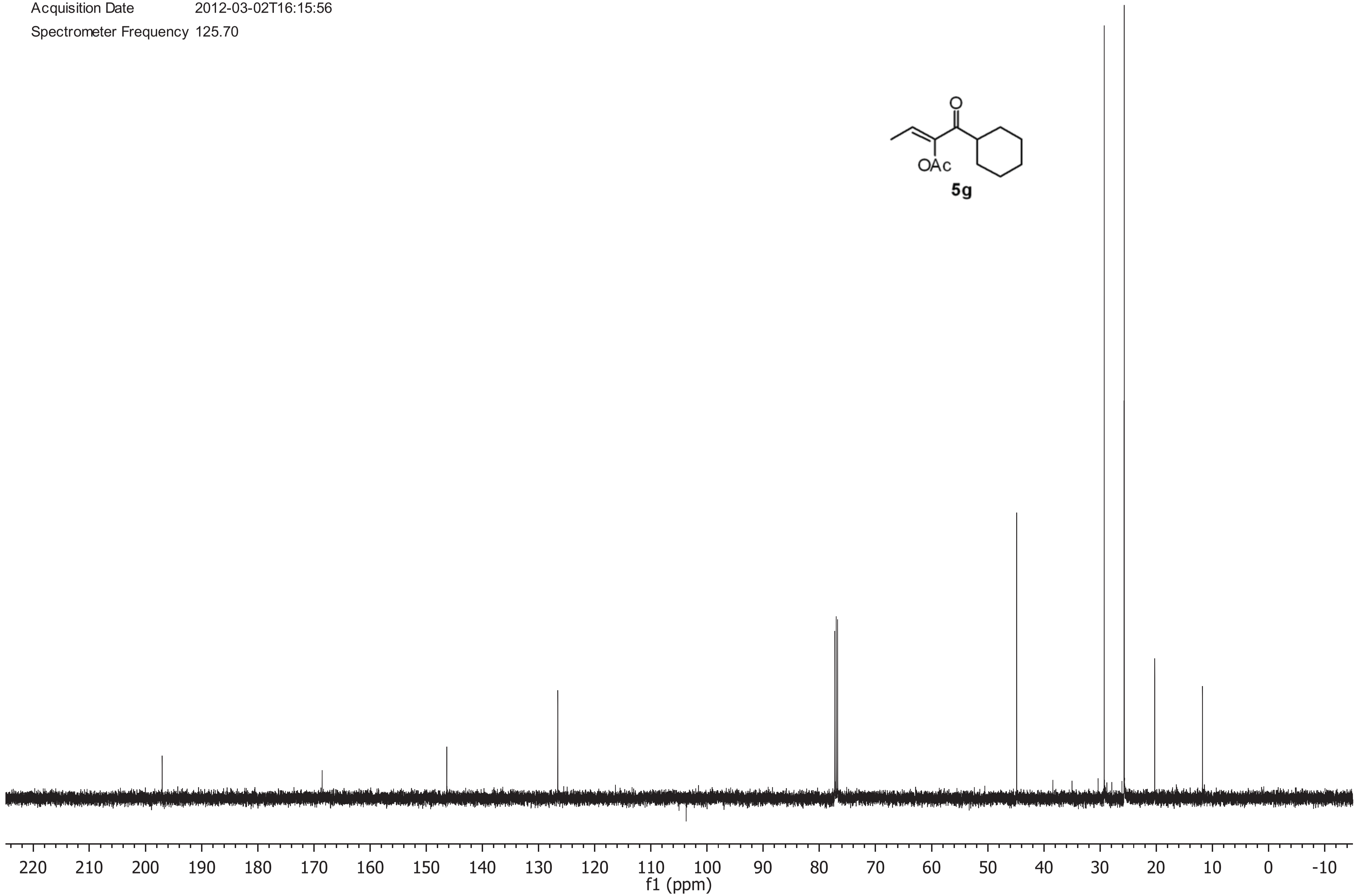

Data File Name	C:/ Users/ zhanglab1/ Desktop/ NMR/ jikg/ product/ jkg-III-59B-1-H.fid/ fid
Title	jkg-III-59B-1-H
Solvent	CDCl3
Acquisition Date	2012-05-06T21:32:38
Spectrometer Frequency	499.86

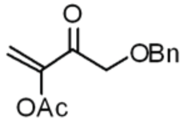

5h

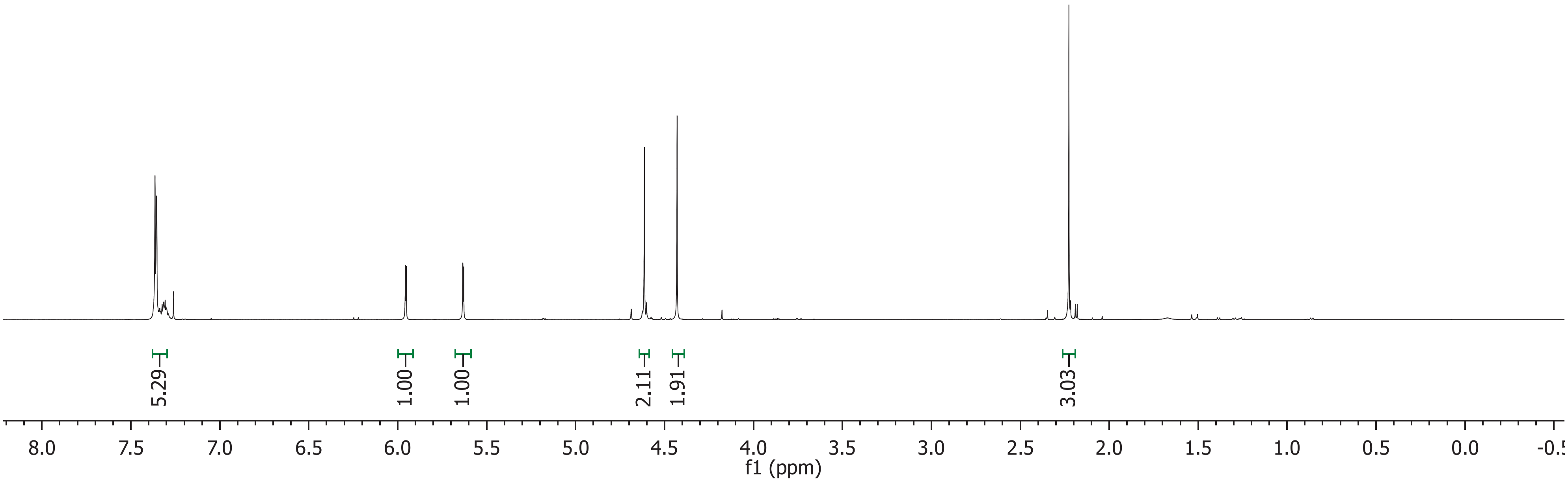

Data File Name	C:/ Users/ zhanglab1/ Desktop/ NMR/ jikg/ product/ jkg-III-59B-1-C.fid/ fid
Title	jkg-III-59B-1-C
Solvent	CDCl3
Acquisition Date	2012-05-06T21:35:46
Spectrometer Frequency	125.70

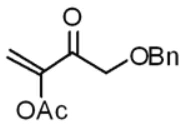

5h

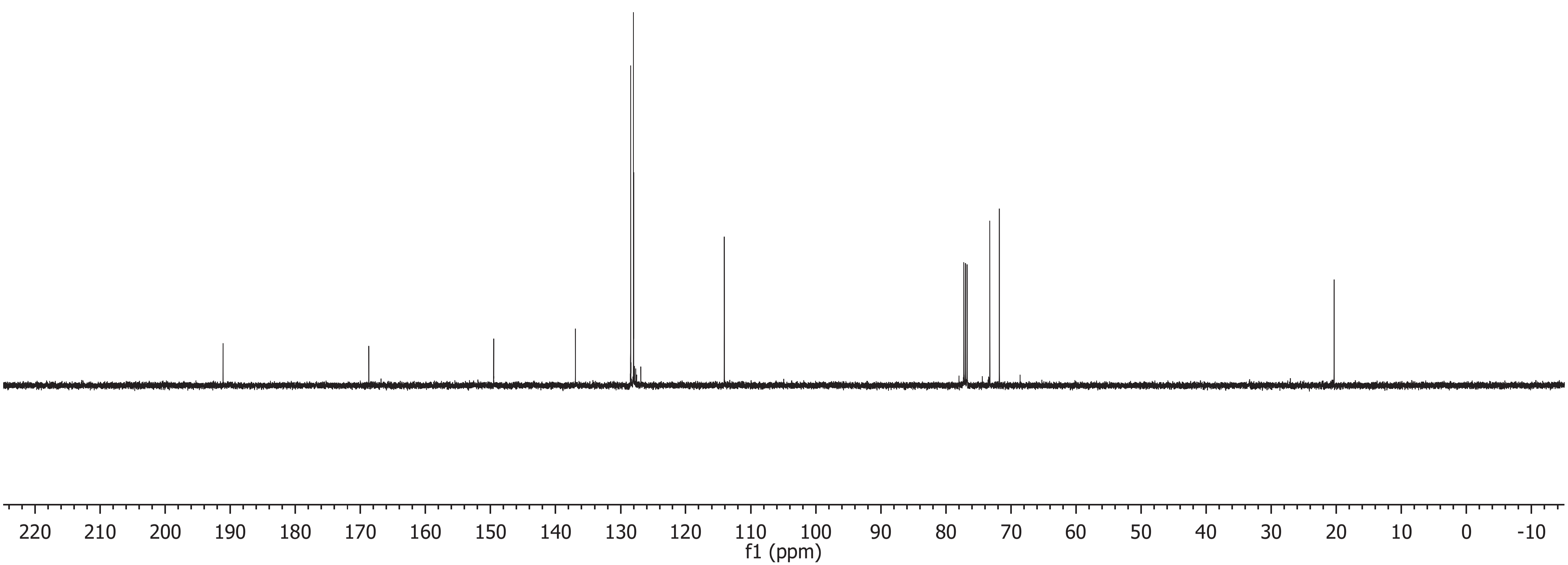

Data File Name	C:/ Users/ zhanglab1/ Desktop/ NMR/ jikg/ product/ jkg-II-251-P-H.fid/ fid
Title	jkg-II-251-P-H
Solvent	cdcl3
Acquisition Date	2012-03-09T22:56:40
Spectrometer Frequency	599.63

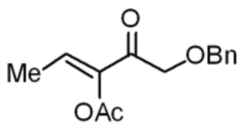

5i

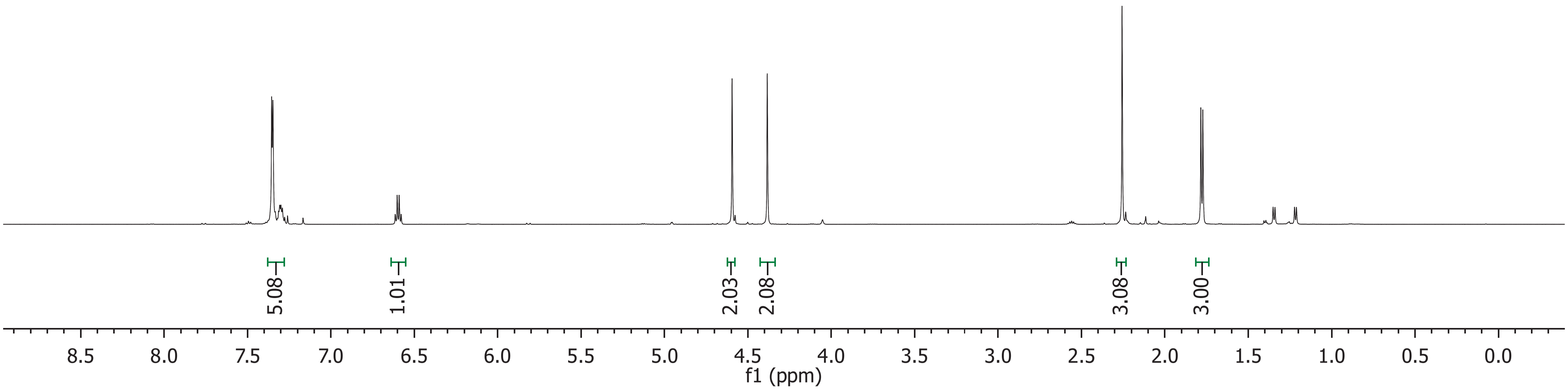

Data File Name	C:/ Users/ zhanglab1/ Desktop/ NMR/ jikg/ product/ jkg-II-251-P-C.fid/ fid
Title	jkg-II-251-P-C
Solvent	cdcl3
Acquisition Date	2012-03-11T17:09:20
Spectrometer Frequency	150.79

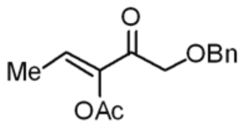

5i

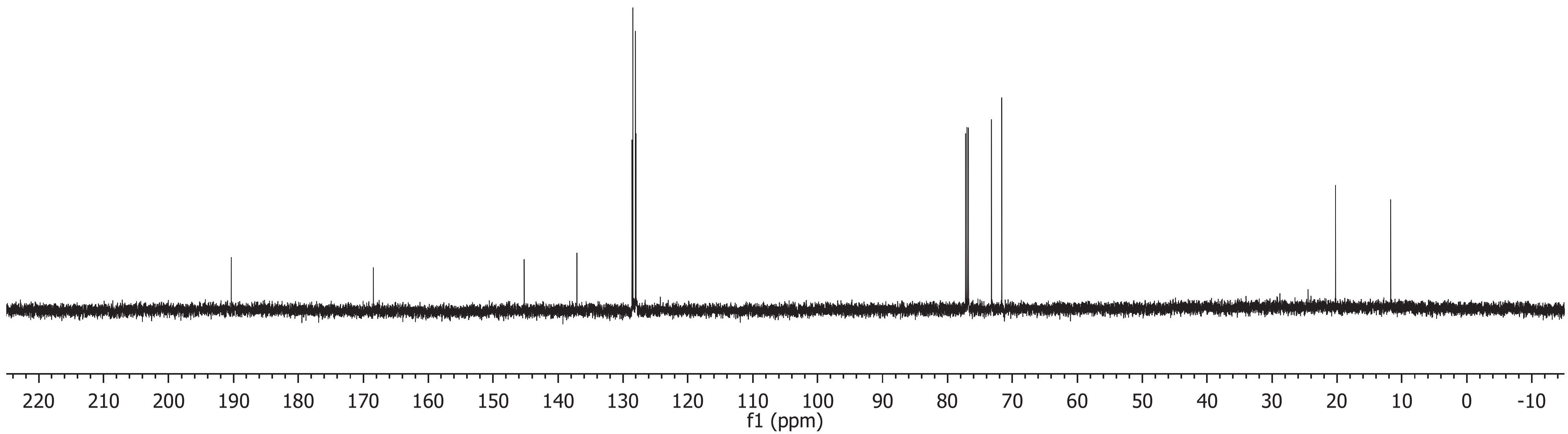

Data File Name	C:/ Users/ zhanglab1/ Desktop/ NMR/ jikg/ product/ jkg-II-248A-P-H.fid/ fid
Title	jkg-II-248A-P-H
Solvent	cdcl3
Acquisition Date	2012-03-08T16:19:55
Spectrometer Frequency	599.63

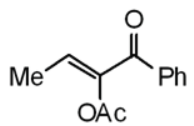

5j

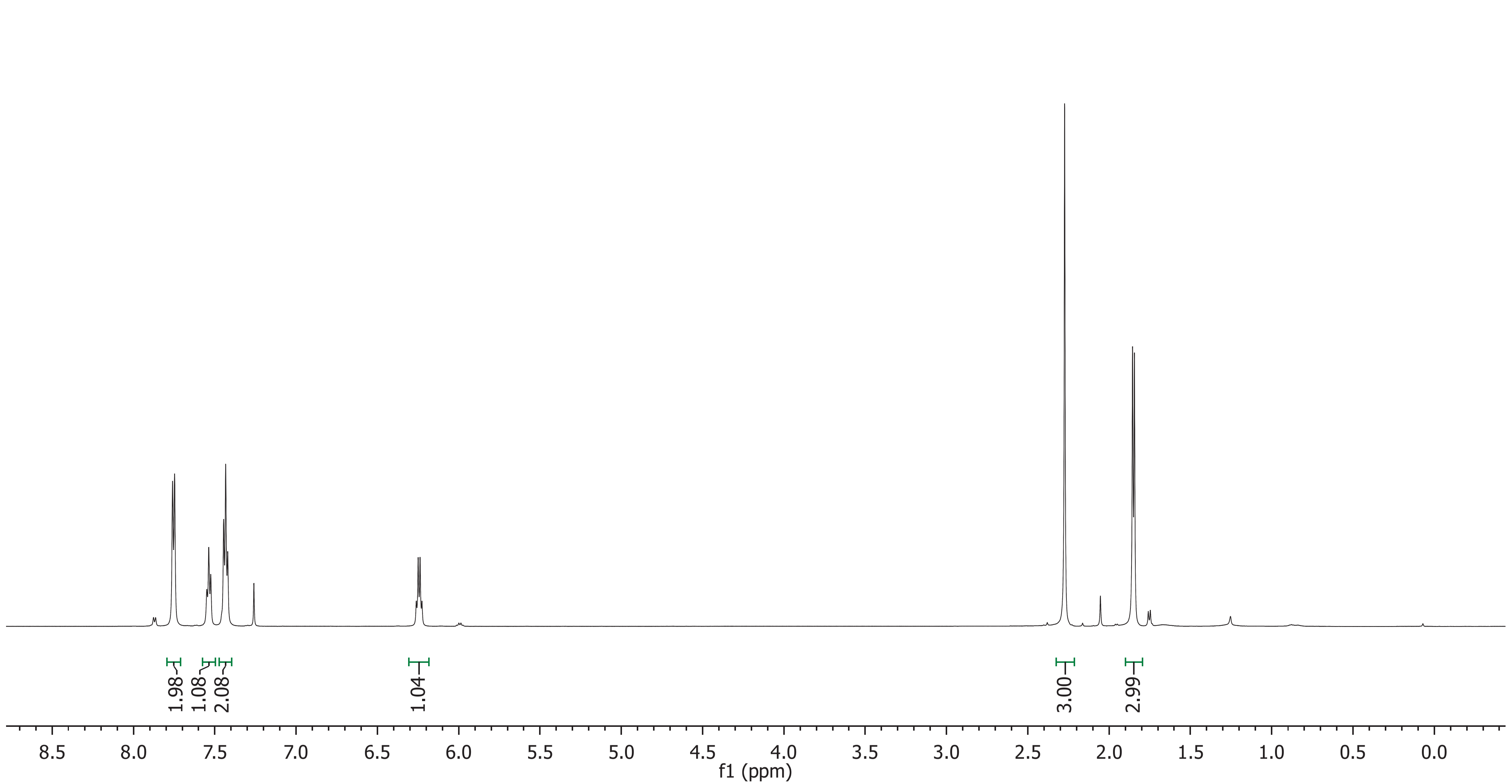

Data File Name	C:/ Users/ zhanglab1/ Desktop/ NMR/ jikg/ product/ jkg-II-248A-P-C1.fid/ fid
Title	jkg-II-248A-P-C1
Solvent	cdcl3
Acquisition Date	2012-03-08T15:05:54
Spectrometer Frequency	150.79

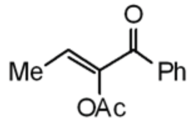

5j

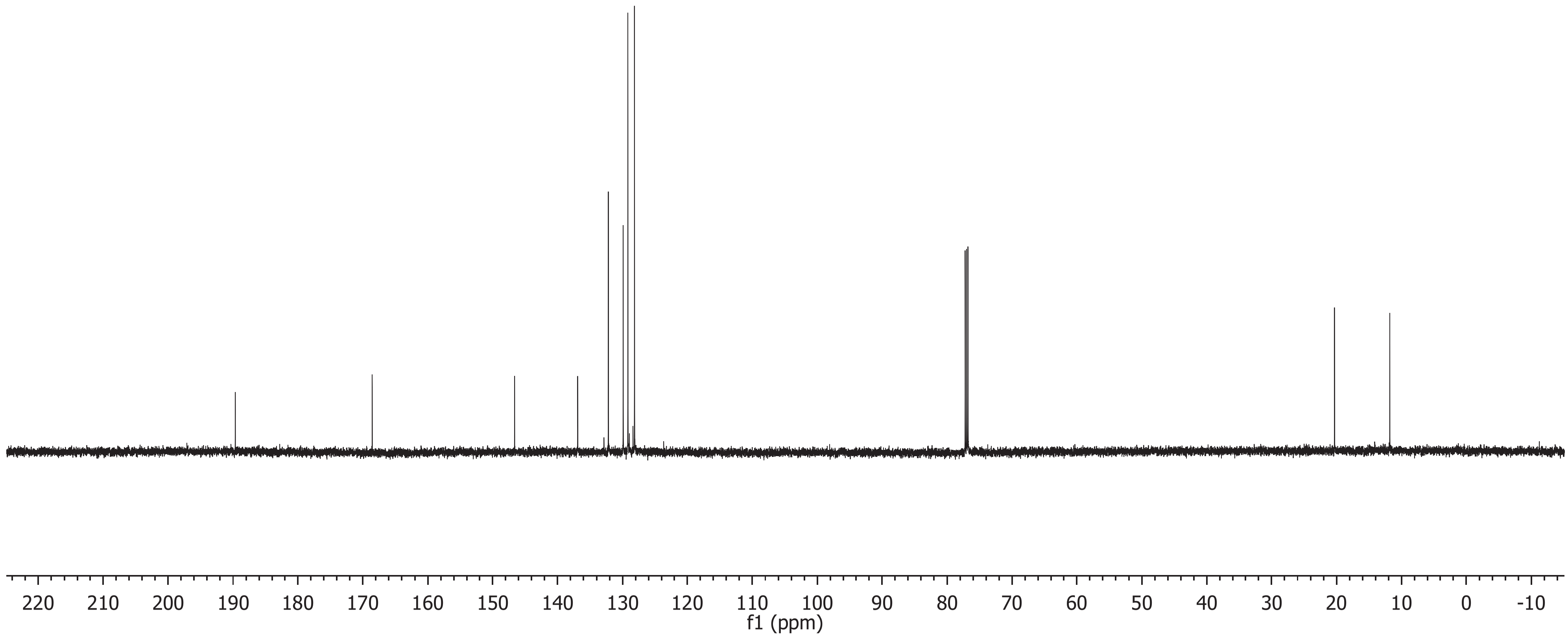

Data File Name	C:/ Users/ zhanglab1/ Desktop/ NMR/ jikg/ product/ jkg-II-233A-P-H.fid/ fid
Title	jkg-II-233A-P-H
Solvent	CDCl3
Acquisition Date	2012-03-01T09:39:02
Spectrometer Frequency	499.86

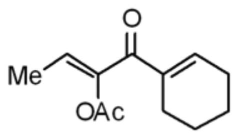

5k

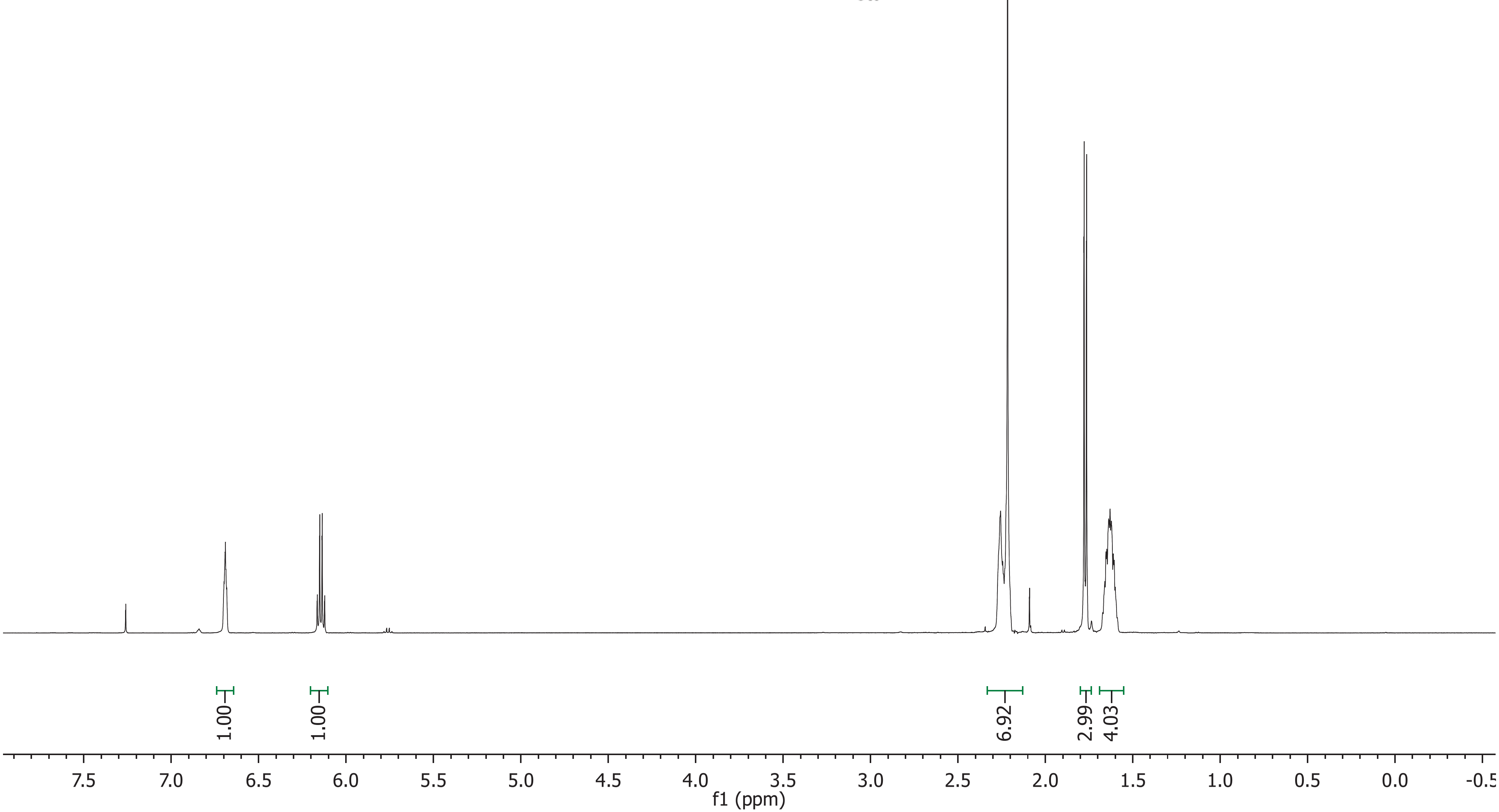

Data File Name	C:/ Users/ zhanglab1/ Desktop/ NMR/ jikg/ product/ jkg-II-233A-P-C.fid/ fid
Title	jkg-II-233A-P-C
Solvent	CDCl3
Acquisition Date	2012-03-01T09:42:03
Spectrometer Frequency	125.70

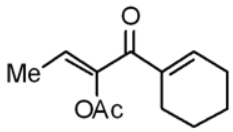

5k

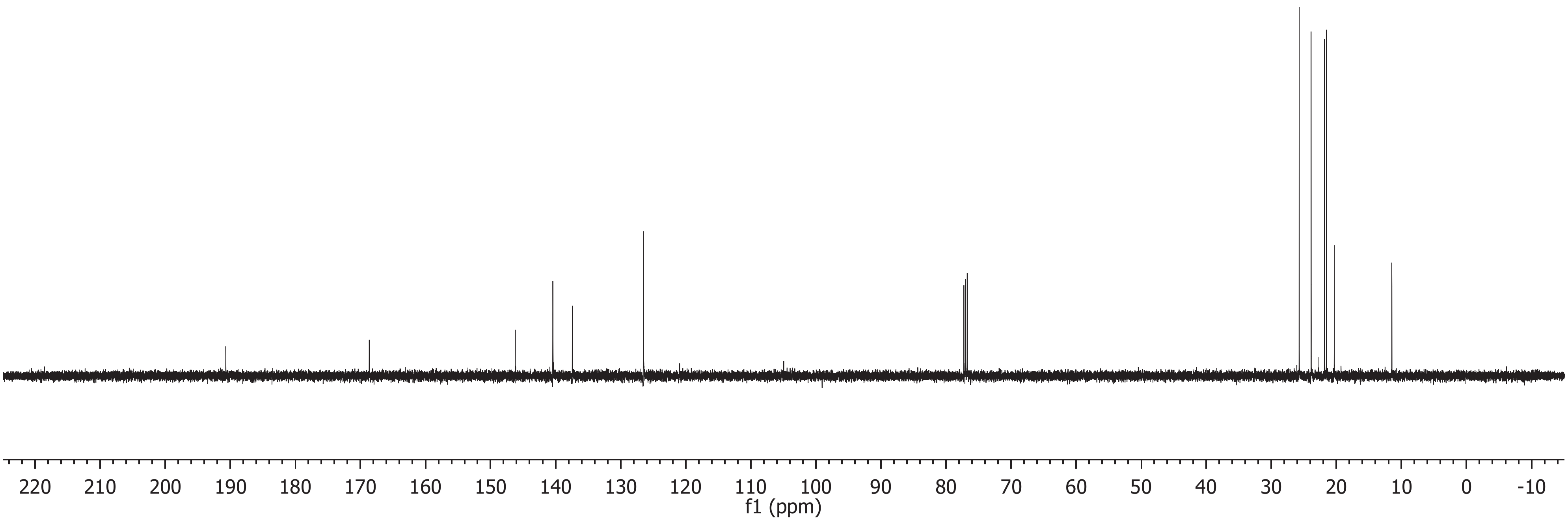

Data File Name	C:/ Users/ zhanglab1/ Desktop/ NMR/ jikg/ product/ jkg-II-246B-P-H.fid/ fid
Title	jkg-II-246B-P-H
Solvent	CDCl3
Acquisition Date	2012-03-06T22:19:47
Spectrometer Frequency	499.86

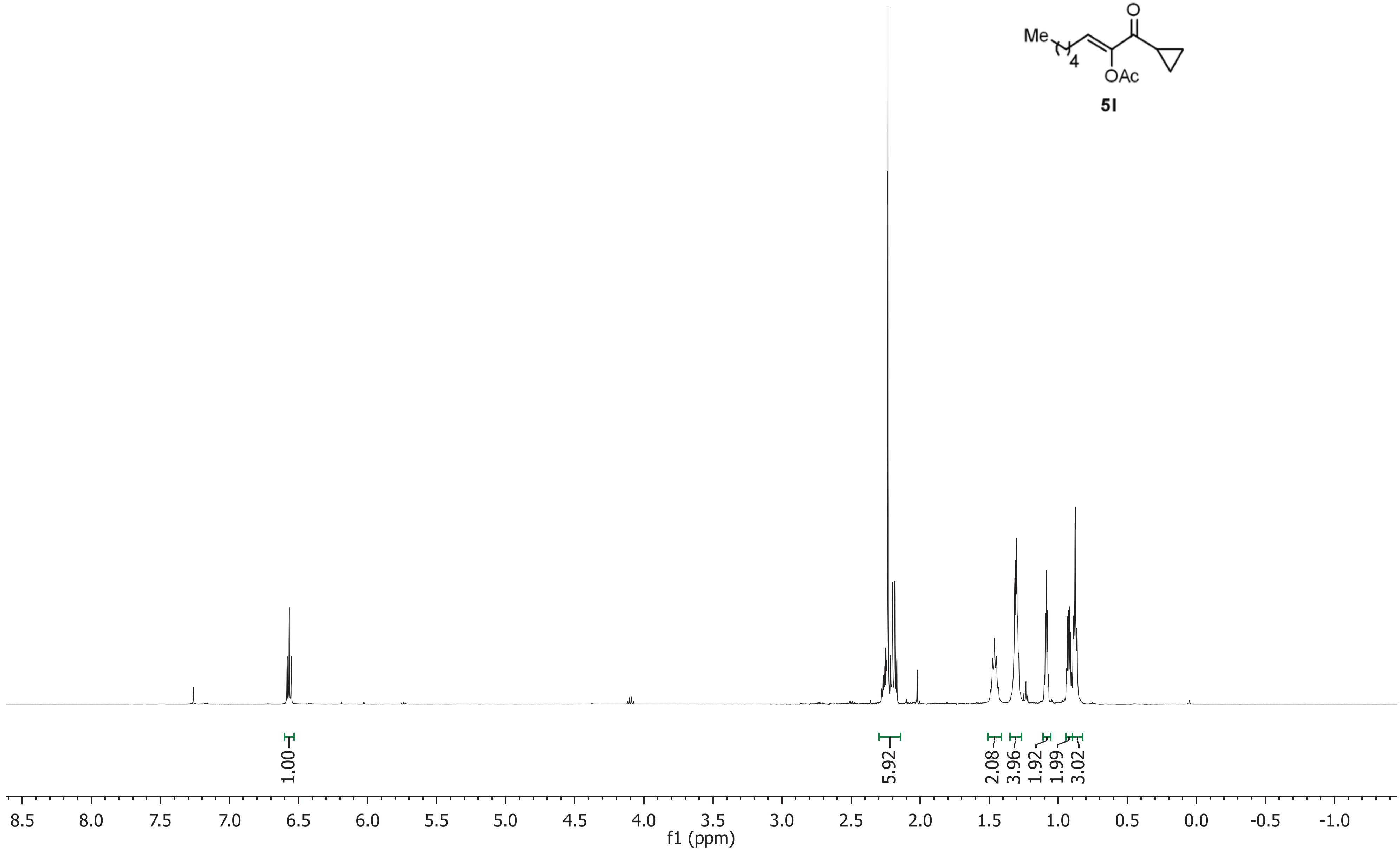

Data File Name	C:/ Users/ zhanglab1/ Desktop/ NMR/ jikg/ product/ jkg-II-246B-P-C.fid/ fid
Title	jkg-II-246B-P-C
Solvent	CDCl3
Acquisition Date	2012-03-06T22:22:48
Spectrometer Frequency	125.70

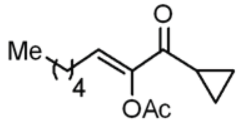

5l

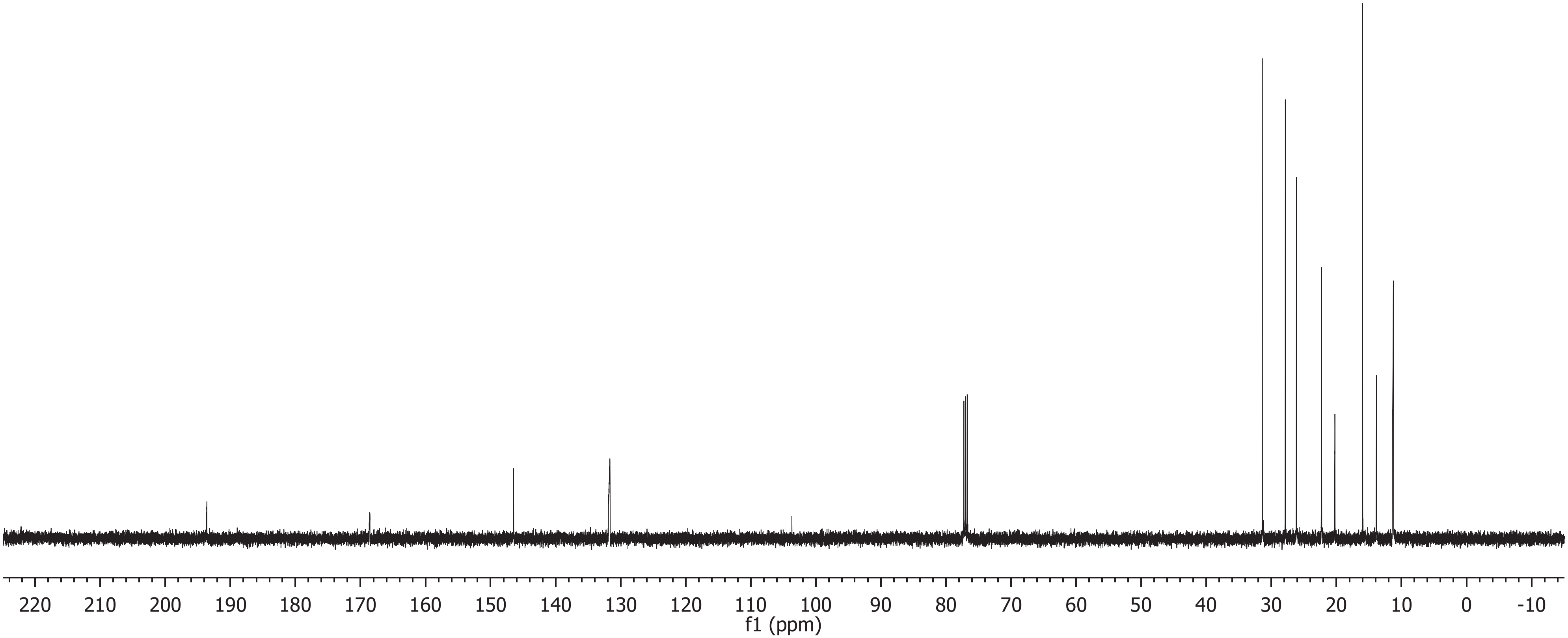

Data File Name	C:/ Users/ zhanglab1/ Desktop/ NMR/ jikg/ product/ jkg-II-240B-P-H.fid/ fid
Title	jkg-II-240B-P-H
Solvent	cdcl3
Acquisition Date	2012-03-05T21:01:39
Spectrometer Frequency	599.63

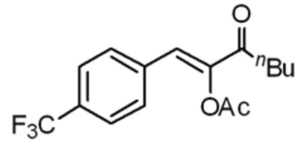

5m

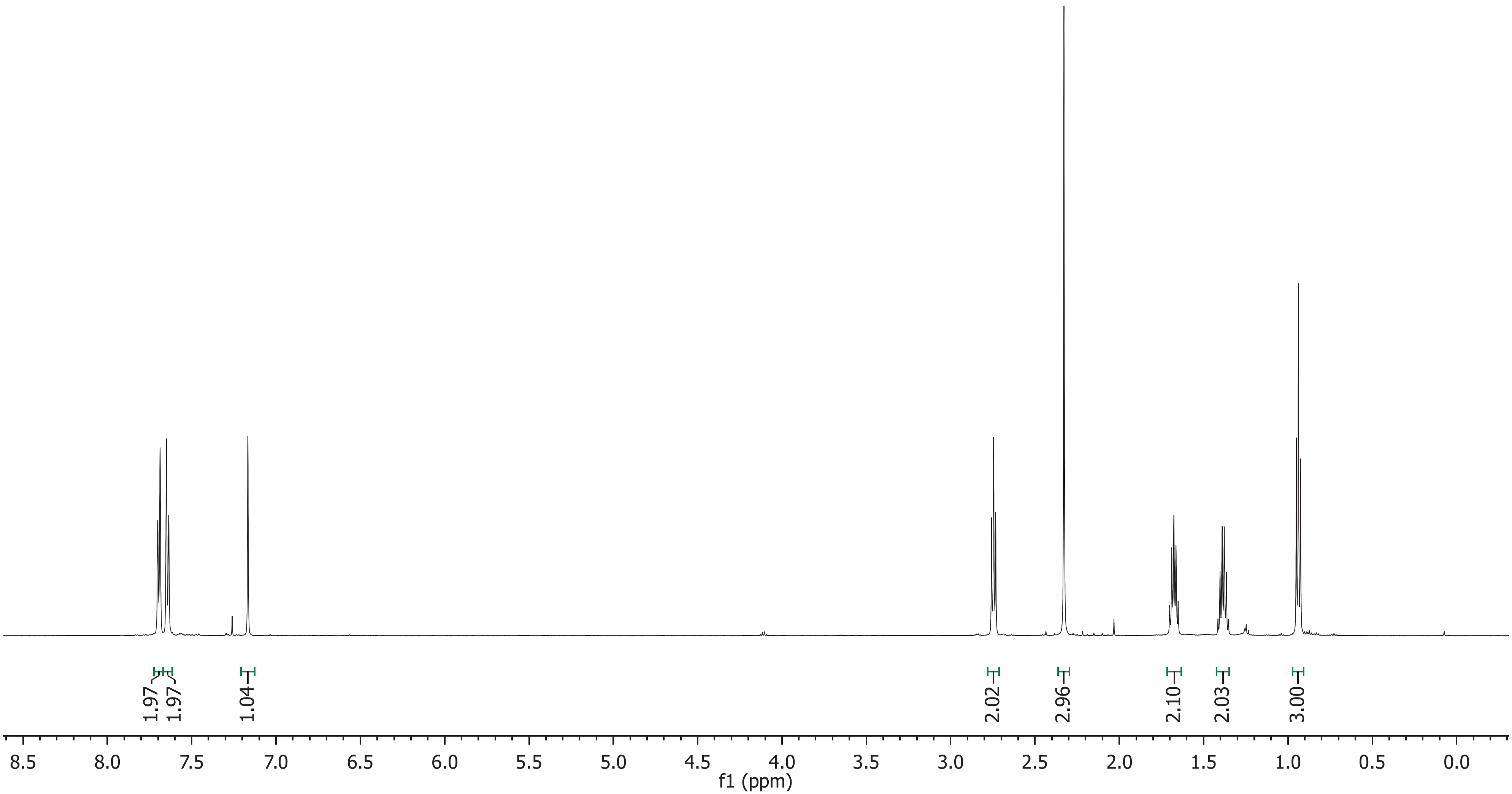

Data File Name	C:/ Users/ zhanglab1/ Desktop/ NMR/ jikg/ product/ jkg-II-240B-P-C.fid/ fid
Title	jkg-II-240B-P-C
Solvent	cdcl3
Acquisition Date	2012-03-05T21:04:17
Spectrometer Frequency	150.79

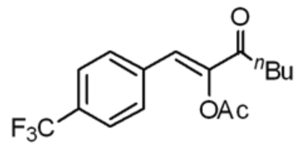

5m

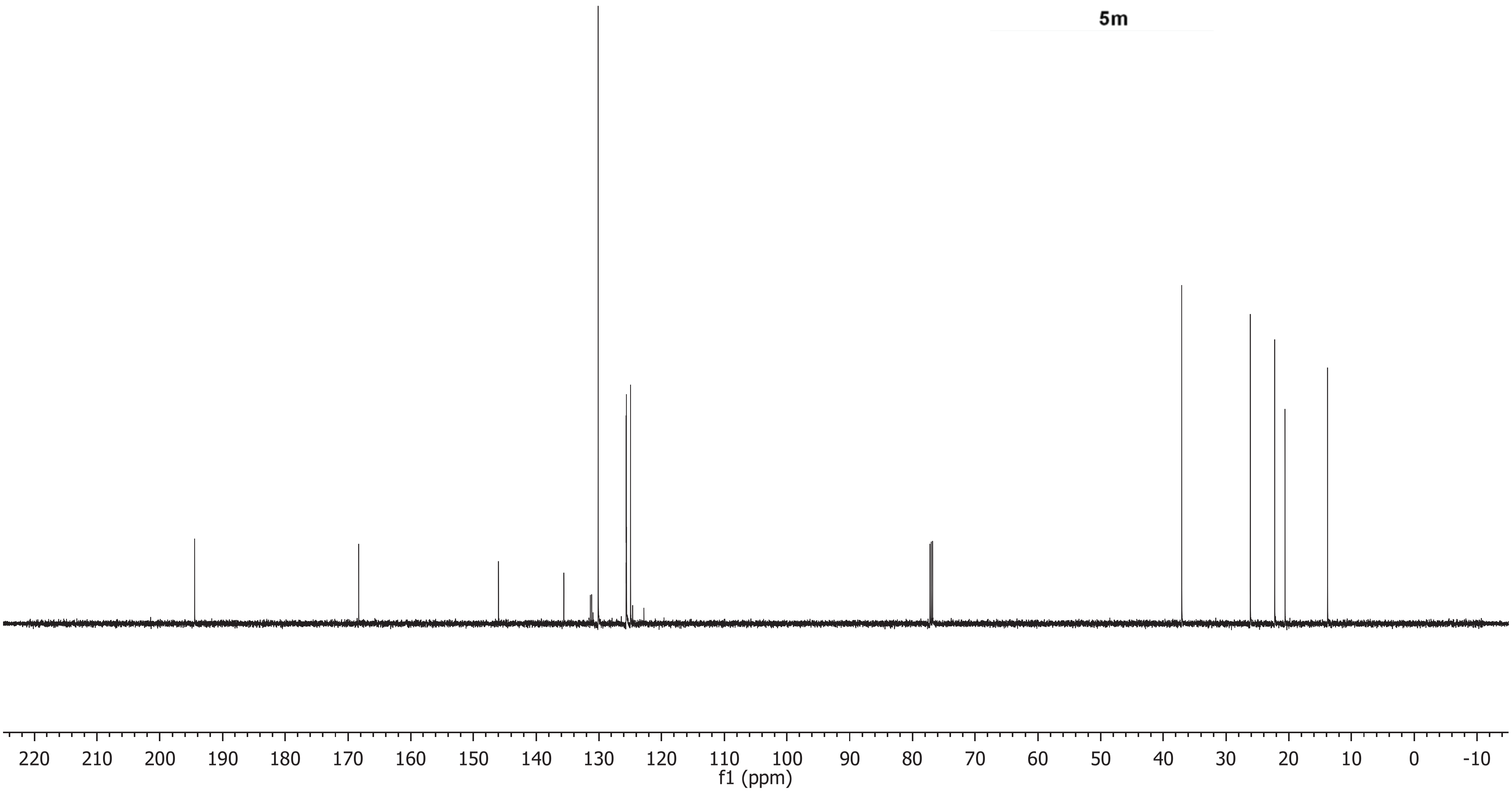

Supplement: File 1 — Experimental procedure, compound characterization, and NMR spectra. [file Beilstein_J_Org_Chem-09-1925-s001.pdf]
